# Ethnobotanical study of Hakka traditional medicine in Ganzhou, China and their antibacterial, antifungal, and cytotoxic assessments

**DOI:** 10.1186/s12906-022-03712-z

**Published:** 2022-09-19

**Authors:** Haibo Hu, Yanfang Yang, Abdallah Aissa, Volkan Tekin, Jialin Li, Sujogya Kumar Panda, Hao Huang, Walter Luyten

**Affiliations:** 1 National Engineering Research Center for Modernization of Traditional Chinese Medicine - Hakka Medical Resources Branch, School of Pharmacy, Gannan Medical University, Ganzhou, 341000 China; 2 Animal Physiology and Neurobiology Section, Department of Biology, KU Leuven, 3000 Leuven, Belgium; 3 Centre de Recherche Scientifique et Technique en Analyses Physicochimiques (CRAPC), BP384, Bou-Ismail, 42004 Tipaza, RP Algeria; 4 Department of Zoology, Utkal University, Vani Vihar, Bhubaneswar, Odisha 751004 India

**Keywords:** Hakka herbs, Hakka traditional medicine, Antibacterial activity, Antifungal activity, Cytotoxicity, Ganzhou, Gannan

## Abstract

**Background:**

Traditional herbs played a crucial role in the health care of the Hakka people. However, studies to identify these traditional herbs are few. Here we document and assess the potential of these plants for treating microbial infections. Many herbs used by the Hakka people could potentially be a novel medicinal resource.

**Methods:**

Local herb markets were surveyed via semi-structured interviews, complemented by direct observations to obtain information on herbal usage. For each herb selected for this study, extracts in four different solvents were prepared, and tested for activity against 20 microorganisms, as well as cancerous and noncancerous cells. All data were subjected to cluster analysis to discover relationships among herbs, plant types, administration forms, solvents, microorganisms, cells, etc., with the aim to discern promising herbs for medicine.

**Results:**

Ninety-seven Hakka herbs in Ganzhou were documented from 93 plants in 62 families; most are used for bathing (97%), or as food, such as tea (32%), soup (12%), etc. Compared with the Chinese Pharmacopoeia and Chinese Materia Medica, 24 Hakka medicines use different plant parts, and 5 plants are recorded here for the first time as traditional medicines. The plant parts used were closely related with the life cycle: annual and perennial herbs were normally used as a whole plant, and woody plants as (tender) stem and leaf, indicating a trend to use the parts that are easily collected. Encouragingly, 311 extracts (94%) were active against one or more microorganisms. Most herbs were active against Gram-positive bacteria, such as *Staphylococcus aureus* (67%), *Listeria innocua* (64%), etc. Cytotoxicity was often observed against a tumor cell, but rarely against normal cells. Considering both antimicrobial activity and cytotoxicity, many herbs reported in this study show promise as medicine.

**Conclusion:**

Hakka people commonly use easily-collected plant parts (aerial parts or entire herb) as medicine. External use of decoctions dominated, and may help combating microbial infections. The results offer promising perspectives for further research since little phytopharmacology and phytochemistry has been published to date.

**Graphical Abstract:**

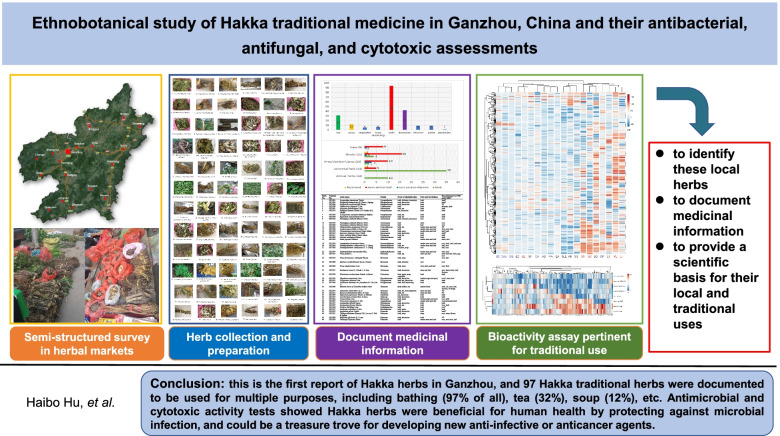

**Supplementary Information:**

The online version contains supplementary material available at 10.1186/s12906-022-03712-z.

## Background

As the primary source of natural products, plants have provided abundant health benefits to man from prehistoric times, especially as food and medicine [1]. In the twenty-first century, with the increased understanding of the pharmacological effects, herbal medicine has been considered as promising for healthcare [2], and around 80% of the world’s population is estimated to use traditional medicine according to the WHO [3]. Different cultures throughout the world have relied heavily on plants for their preventive, curative, health-restoring and -boosting abilities, such as traditional Chinese medicine (TCM), Ayurveda, Siddha, Unani, Kampo, Jamu, Thai herbal medicines, etc., among which TCM knowledge has been recorded for nearly 5000 years [4–8]. Since the last century, China’s policies and development have brought about a hitherto unprecedented development of TCM. Its holistic and systematic development has resulted in an increase in the number of approved TCMs. The Chinese Pharmacopoeia (2020 version) lists 2711 TCMs and their preparations, in which 177 more items were recorded than in the 2015 version, because more and more folk herbs were included due to their increasing development and usage [9, 10].

**Fig. 1:**
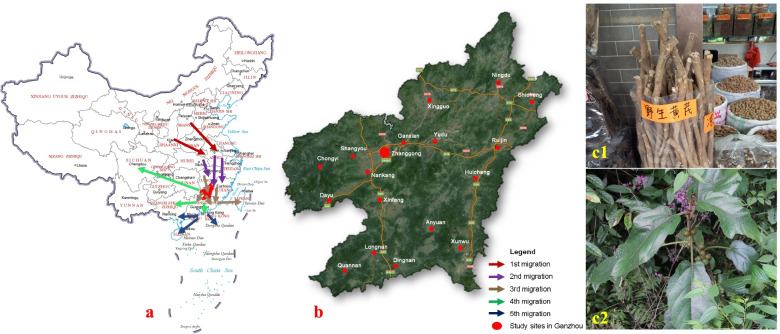
The major migration routes of Hakka (**a**), study sites in Ganzhou (**b**) and a HTM example of TCM replacement in the Hakka area (**c**). *Ficus hirta* Vahl. (**c2**) was locally named ‘Wu zhi mao tao’, ‘Tu huang qi’ or ‘Nan qi’ as an alternative of northern distributed *Astragali Radix* or ‘Huang qi’ (**c1**). Figure 1-**a** and 1-**b** are based on the public resources (No. GS(2019)1655, produced by Ministry of Natural Resources of the People’s Republic of China, and Baidu map https://map.baidu.com, respectively), while **c1–2** were photographed by one of the authors (Haibo Hu)

Hakka traditional medicine or herbs (HTM), as a kind of folk medicine, originated from the migration of the Hakka people, who belong to a branch of the Han nationality, and are therefore not an ethnic minority. The Hakkas moved South from the Central Plains (North of China) over more than 1000 km to settle down in South China; this migration occurred in various stages (Fig. 1-a) [11–13]. After these southward migrations, they could not easily access many TCM resources used in the North for disease prevention and treatment, such as *Glycyrrhizae radix et rhizoma*, *Rhei radix et rhizoma*, *Astragali radix* (Fig. 1-c1), *Angelicae sinensis radix*, *Ginseng radix et rhizoma*, etc. Therefore, the Hakkas turned to developing and using local herbal resources, and gradually formed a Hakka medical model with regional characteristics [14–16]. In China, Hakka areas mainly include southern Jiangxi (Ganzhou), southwestern Fujian, northern Guangdong, etc. However, so far, HTM investigation and research have been conducted in the Guangdong and Fujian areas. There were hundreds of HTM plant species reported, a considerable proportion of which were used both for food and medicinal purposes. For example, the soup made of a medicinal herb was used to prevent disease and promote health, because these wild edible and medicinal plants were considered as an important part of the traditional diets, and can also contribute to nutrition and food security [17–26]. The sharp distinction between food and medicine typically made in the West is foreign to Chinese thinking.

As the cradle area of Hakkas, Ganzhou (also called Gannan) has housed one of the largest Hakka populations, accounting for nearly 10 million, with southern Jiangxi as the largest administrative area. After several centuries of migrations, more and more local herbs were utilized by Hakkas in Ganzhou, and a tradition of trading HTMs has developed, with very prosperous local herb markets. Especially when the season changes between spring and summer, herb markets are held, and many Hakka people collect herbs from the wild mountains to sell them in those markets [27]. Most Hakkas are used to boil the herbs for internal and external use, such as bathing in these decoctions to prevent and cure some infections. In addition, herbal cuisine uses the herbs to enhance the taste of foods and produce therapeutic effects. However, reports on HTMs in Ganzhou are still scarce, especially ethnobotanical studies, and the use of HTMs by Ganzhou Hakkas largely relied on oral traditions being passed between generations. Most of this knowledge has not been published or documented. Thus, this paper focuses on HTMs in Ganzhou, aiming to record valuable knowledge concerning HTMs in danger of being lost. Moreover, most HTMs in this study are used for bathing purposes to protect skin or cure skin diseases, mainly caused by microbial infections. It indicates these herbs should be active against different human pathogens. Hence, assessing their antimicrobial activities is necessary to confirm their therapeutic applications. Also, due to the common use of HTMs as food, it is necessary to evaluate their toxicity. In sum, this study is to record HTMs in Ganzhou scientifically and assess their bioactivities to validate their use in treating infectious diseases.

## Materials and methods

### Study area

As the largest Hakka area in the world [28], Ganzhou has a humid subtropical monsoon climate, with hot, wet summers and mild, dry winters. Ganzhou is located (113°54′ E to 116°38′ E longitude and 24°29′ N to 27°09′ N latitude) between the Wuyi-, Lingnan- and Luoxiao mountains, at the southern margin of a subtropical zone (Fig. 1-a). Several rivers cut across these mountains and form a low-lying terrain where hilly areas dominate with an average altitude of 300 ~ 500 m. The average daily temperature highs of Ganzhou in January and July are 11 and 34 °C, respectively. With almost 80% forest coverage, this area is green through all four seasons. Frost is rare, but may happen a few days each winter. The mean annual rainfall is around 1600 mm. The unique location on the northern side of the Lingnan Mountains, and the edge of a subtropical zone, along with the humid climate and high forest coverage, make this reserve a treasure of biodiversity with rare, endangered, or threatened plants and animals [29]. In this region, medicinal plants play an important social and cultural role, and sometimes may be the only alternative available to treat health problems for this population. Eighteen administrative areas maintain similar Hakka traditions (Fig. 1-b): 3 districts (Zhanggong, Ganxian, and Nankang), and 15 counties (Ningdu, Xingguo, Shicheng, Ruijin, Yudu, Huichang, Shangyou, Chongyi, Dayu, Xinfeng, Longnan, Quannan, Dingnan, Anyuan and Xunwu). Most people in Ganzhou are Hakkas, reaching over 95% [30, 31]. Hakka people developed their unique culture, distinct from the traditional culture of southern Hans, including differences in dialects, customs, lifestyles, and habits [32]. The local people still depend to some extent on the indigenous system of Hakka traditional medicine.

### Market investigation and herb collection

The study team was composed of pharmacognosts, ethnopharmacologists, botanists, and TCM students from the School of Pharmacy, Gannan Medical University. The eighteen counties or districts of Ganzhou were chosen as the study sites to investigate the local herb markets during the Dragon Boat Festival [33] from 2016 to 2018 (Fig. 1-b). Information was obtained from semi-structured interviews, personal conversations with practitioners, and direct observation by generally acceptable methods (Supplementary material 1), and by reviewing previous studies of Hakka herbs or medicines in the scientific literature (SciFinder, Wanfang database, Cnki database.) [34, 35].

A total of 97 Hakka herbs were collected from herbal medicinal hawkers in 2018, and all the herb data were confirmed by more than 3 hawkers or herb buyers, including the local use, the preparation, forms of administration, etc. In this paper, we focused on the administration forms. The herbs were identified by Prof. Haibo Hu and Prof. Jialin Li based on the “Flora of China”, or their morphological, microscopic, physical or chemical characteristics by following standard authentication methods of TCM [36]. Voucher specimens are stored in the Herbarium of Chinese Medicine of Gannan Medical University. The plant information with their herbarium numbers, local use and plant part(s), is listed according to the Cronquist system of classification in Table 1.

**Table 1:** HTMs collected from the local herbal markets in Ganzhou, China

Herb No.	Voucher No.	Plant name	Family	Form of administration	Part used by Hakkas	Recorded part in CPH and CMM ^*^	Application
1	GZ1801	*Lycopodium japonicum* Thunb.	Lycopodiaceae	bath, infusion, decoction	herb	herb^a^	rheumatism syndrome, skin infection
2	GZ1885	*Selaginella tamariscina* (P. Beauv.) Spring	Selaginellaceae	bath, decoction	herb	herb^a^	foot itching, blooding, traumatic injury
3	GZ1886	*Selaginella moellendorffii* Hieron.	Selaginellaceae	bath, decoction	herb	NT	detoxication, diuresis
4	GZ1828	*Odontosoria chinensis* J. Sm.	Lindsaeaceae	bath	herb	rhizome, herb	detoxication, rheumatism syndrome
5	GZ1881	*Adiantum flabellulatum* Linn.	Adiantaceae	bath, tea	herb	herb	detoxicating, tongue sore
6	GZ1875	*Selliguea hastata* (Thunb.) H. Ohashi & K. Ohashi	Polypodiaceae	tea, decoction	herb	herb	detoxication, diuresis, stranguria
7	GZ1836	*Loxogramme salicifolia* (Makino) Makino	Polypodiaceae	bath, tea	herb	herb	detoxication, cough
8	GZ1840	*Equisetum ramosissimum* Desf.	Equisetaceae	bath, tea	herb	herb	skin itching, improving eyesight, hepatitis
9	GZ1818	*Fissistigma oldhamii* (Hemsl.) Merr.	Annonaceae	bath, infusion, decoction	root	root	rheumatism syndrome, promoting blood circulation, body pain
10	GZ1819	*Fissistigma oldhamii* (Hemsl.) Merr.	Annonaceae	bath	stem	root	rheumatism syndrome, skin infection
11	GZ1820	*Fissistigma oldhamii* (Hemsl.) Merr.	Annonaceae	bath	leaf	root	rheumatism syndrome, skin infection
12	GZ1805	*Chimonanthus grammatus* M.C.Liu	Calycanthaceae	bath, tea	tender stem and leaf	NT	cold, infection, insecticide
13	GZ1850	*Lindera glauca* (Sieb. et Zucc.) Bl.	Lauraceae	bath	tender stem and leaf	fruit, root, leaf	body pain, cold, infection, skin disease
14	GZ1878	*Cinnamomum jensenianum* Hand.-Mazz.	Lauraceae	bath, soup	tender stem and leaf	leaf, bark	cold, infections, skin disease
15	GZ1843	*Saururus chinensis* (Lour.) Baill.	Saururaceae	bath, decoction	herb	herb^a^	eczema, urinary tract infection
16	GZ1811	*Piper wallichii* (Miq.) Hand.-Mazz.	Piperaceae	bath, tea	herb	herb	rheumatism syndrome, tonifying kidney
17	GZ1890	*Asarum caudigerum* Hance	Aristolochiaceae	bath	herb	herb	insecticide, foot itching
18	GZ1834	*Sabia japonica* Maxim.	Sabiaceae	bath	tender stem and leaf	stem, root	dermatophytosis, rheumatism syndrome, detoxicating
19	GZ1810	*Liquidambar formosana* Hance	Hamamelidaceae	bath	tender stem and leaf	root, bark, leaf, and resin	rheumatism syndrome, skin itching
20	GZ1862	*Semiliquidambar cathayensis* H. T. Chang	Hamamelidaceae	bath, tea	tender stem and leaf	leaf, root	rheumatism syndrome, arthritis
21	GZ1883	*Semiliquidambar cathayensis* H. T. Chang	Hamamelidaceae	infusion, soup	root	leaf, root	rheumatism syndrome, body pain, traumatic injury
22	GZ1824	*Daphniphyllum macropodum* Miq.	Daphniphyllaceae	bath	tender stem and leaf	leaf, seed	sore ulcer, rheumatism syndrome
23	GZ1813	*Ficus pumila* L.	Moraceae	bath, tea	stem and leaf	stem and leaf, fruit, sap, root	dysentery, menstruation disorder, rheumatism syndrome
24	GZ1816	*Ficus formosana* f. *shimadai* Hayata	Moraceae	bath, soup	herb	root, leaf	enrich milk secretion and the blood, dysentery
25	GZ1880	*Maclura cochinchinensis* (Lour.) Corner	Moraceae	bath, infusion	root	root	skin infection, rheumatism syndrome, detoxication
26	GZ1869	*Ficus simplicissima* Lour.	Moraceae	bath, soup	root, stem, and leaf	root, fruit	rheumatism syndrome, enrich milk secretion
27	GZ1851	*Boehmeria nivea* (L.) Hook. f. et Arn.	Urticaceae	bath, decoction	stem and leaf	root, stem, bark, leaf, flower	detoxication, diuresis
28	GZ1838	*Elatostema involucratum* Franch. et Savat.	Urticaceae	bath, paste, soup, vegetable	herb	herb	detoxication, diuresis, edema
29	GZ1854	*Phytolacca americana* Linn.	Phytolaccaceae	bath, decoction	herb(overground part)	root^a^, seed, leaf	detoxication, diuresis
30	GZ1812	*Polygonum chinense* Linn.	Polygonaceae	bath, tea, decoction	herb	herb	detoxication, dysentery, cold
31	GZ1846	*Persicaria chinensis* var. *paradoxa* (H. Lév.) Bo Li	Polygonaceae	bath, food seasoning	herb	herb	detoxication, dysentery, diarrhea
32	GZ1806	*infected leaves of Camellia oleifera* Abel	Theaceae	fresh edible, tea	infected leaf	seed, oil, root, leaf, flowers, oil dreg	dysentery
33	GZ1814	*Adinandra nitida* Merr. ex Li	Theaceae	bath, tea, food pigments	stem and leaf	NT	infection, detoxication
34	GZ1815	*Eurya acuminatissima* Merr. et Chun	Theaceae	bath, tea	stem and leaf	NT	infection, detoxication
35	GZ1894	*Hypericum japonicum* Thunb. ex Murray	Guttiferae	tea, decoction	herb	herb	detoxication, hepatitis
36	GZ1844	*Corchoropsis crenata* Sieb. et Zucc.	Tiliaceae	bath, decoction	herb	herb	indigestion, detumescence, skin infection
37	GZ1864	*Urena lobata* Linn.	Malvaceae	bath, paste	herb(overground part)	root, herb	cold, dysentery, rheumatism syndrome,
38	GZ1867	*Pterocarya stenoptera* C. DC.	Juglandaceae	bath	tender stem and leaf	root, bark, stem, fruit	insecticide, foot itching
39	GZ1837	*Lysimachia alfredii* Hance	Primulaceae	bath, decoction	herb	herb	diuresis, stranguria
40	GZ1857	*Lysimachia fortunei* Maxim.	Primulaceae	bath, tea	herb	root, herb	dysentery, detoxication, sore throat
41	GZ1832	*Dichroa febrifuga* Lour.	Hydrangeaceae	bath	herb	root^a^	detoxication, insecticide
42	GZ1847	*Saxifraga stolonifera* Curtis	Saxifragaceae	bath, paste	herb	herb	detoxication, infection, inflammation
43	GZ1855	*Agrimonia pilosa* Ledeb.	Rosaceae	bath, decoction	herb	herb^a^	insecticide, detoxication, astringent
44	GZ1821	*Callerya dielsiana* (Harms) P.K. Loc ex Z. Wei & Pedley	Fabaceae	bath, soup	stem	root, stem, flower	rheumatism syndrome, arthralgia, menstruation disorder
45	GZ1889	*Melilotus officinalis* (L.) Lam.	Fabaceae	bath, decoction	herb	NT	detoxication, dysentery
46	GZ1859	*Dalbergia hupeana* Hance	Fabaceae	bath	tender stem and leaf	root, root bark, leaf	detoxication, rheumatism syndrome, detumescence
47	GZ1891	*Gonocarpus micranthus* Thunb.	Haloragaceae	bath, decoction	herb	herb	dysentery, cough
48	GZ1863	*Melastoma dodecandrum* Lour.	Melastomataceae	bath, decoction, paste	herb	root, herb, fruit	skin diseases, rheumatism syndrome, gastrointestinal infection, snake bite
49	GZ1833	*Scurrula parasitica* Linn.	Loranthaceae	bath, infusion, decoction, soup	tender stem and leaf	stem and leaf	rheumatism syndrome, body pain, skin diseases
50	GZ1831	*Buxus sinica* (Rehd. et Wils.) M. Cheng	Buxaceae	bath, decoction	tender stem and leaf	stem, leaf, root	furunculosis, tooth pain
51	GZ1866	*Phyllanthus glaucus* Wall. ex Muell. Arg.	Euphorbiacea	bath, decoction, paste	tender herb	root	bacillary dysentery, rheumatism syndrome
52	GZ1804	*Nekemias grossedentata* (Hand.-Mazz.) J. Wen & Z. L. Nie	Vitaceae	bath, tea	stem and leaf	stem, leaf, root, root bark	rheumatism syndrome, body pain
53	GZ1802	*Picrasma quassioides* (D. Don) Benn. *Picrasma quassioides* (D. Don) Benn.	Simaroubaceae	bath, decoction	woody stem	stem and leaf^a^, bark, root	skin infection, eczema, enteritidis
54	GZ1803			bath	leaf	stem and leaf^b^, bark, root	skin infection, eczema
55	GZ1887	*Polygala japonica* Houtt.	Polygalaceae	bath, infusion, paste	herb	herb^a^, root	skin disease, cough, insecticide
56	GZ1888	*Polygala angustifolia* (Chodat) R.N. Banerjee	Polygalaceae	bath	herb	NT	skin disease, cough, insecticide
57	GZ1809	*Turpinia arguta* Seem.	Staphyleaceae	bath, decoction, paste	leaf	leaf^a^, root	traumatic injury, skin ulcer, pyogenic infection
58	GZ1845	*Zanthoxylum simulans* Hance	Rutaceae	bath, food seasoning	tender stem and leaf	leaf, fruit, bark	foot skin disease, insecticide, rheumatism syndrome
59	GZ1827	*Aralia elata* (Miq.) Seem.	Araliaceae	bath, decoction	stem	bark, leaf, bud	rheumatism syndrome, dysentery, diarrhea
60	GZ1865	*Heptapleurum heptaphyllum* (L.) Y. F. Deng	Araliaceae	bath, decoction	tender stem and leaf	root, bark, leaf	blooding, rheumatism syndrome, dysentery, body pain
61	GZ1856	*Fatsia japonica* (Thunb.) Decne. et Planch.	Araliaceae	bath, decoction	leaf	root bark, leaf	rheumatism syndrome, traumatic injury
62	GZ1896	*Trachelospermum jasminoides* (Lindl.) Lem.	Apocynaceae	bath, decoction	tender stem and leaf	stem and leaf^a^	skin itching, rheumatism syndrome, body pain
63	GZ1870	*Cynanchum stauntonii* (Decne.) Schltr. ex H.Lév.	Apocynaceae	bath, decoction	herb	rhizome and roots^a^	skin disease, cough, traumatic injury
64	GZ1842	*Physalis angulata* Linn.	Solanaceae	bath, soup, tea, vegetable	herb	herb	dysentery, trachitis, skin infection
65	GZ1882	*Dichondra micrantha* Urb.	Convolvulaceae	bath, tea	herb	herb	dysentery, hepatitis
66	GZ1841	*Evolvulus alsinoides* (Linn.) Linn.	Convolvulaceae	bath, decoction	herb	herb	dysentery, icterus
67	GZ1873	*Verbena officinalis* Linn.	Verbenaceae	bath, paste, decoction	herb	herb^a^	dysentery, body pain, edema
68	GZ1871	*Vitex negundo* var. *cannabifolia* (Sieb. et Zucc.) Hand.-Mazz.	Verbenaceae	bath, food preservatives, decoction	tender stem and leaf	leaf^a^, root, stem, fruit	dysentery, gastroenteritis, influenza
69	GZ1839	*Origanum vulgare* Linn.	Lamiaceae	bath, soup, tea	herb	herb	skin infection, heatstroke, influenza
70	GZ1852	*Salvia prionitis* Hance	Lamiaceae	bath, soup	herb, root	herb	influenza, fever, dysentery, diarrhea
71	GZ1895	*Caryopteris incana* (Thunb.) Miq.	Lamiaceae	bath, infusion, tea	herb	herb	upper respiratory infection, body pain, traumatic injury
72	GZ1893	*Mosla scabra* (Thunb.) C. Y. Wu et H. W. Li	Lamiaceae	bath, tea	herb	herb	influenza, headache, heatstroke, gastroenteritis
73	GZ1817	*Buddleja lindleyana* Fortune	Buddlejaceae	bath, pesticides	fruit	stem and leaf, flower, root	dermatophytosis, insecticide
74	GZ1892	*Siphonostegia chinensis* Benth.	Scrophulariaceae	bath, tea, decoction	herb	herb^a^	dysentery, icterus, hepatitis
75	GZ1807	*Strobilanthes cusia* (Nees) J.B.Imlay	Acanthaceae	bath, tea, food pigments	herb	rhizome and root^a^, leaf	measles, influenza, headache, icterus
76	GZ1808	*Mussaenda pubescens* Dryand.	Rubiaceae	bath, tea	stem and leaf	stem and leaf	detoxication, influenza, pharyngitis
77	GZ1823	*Paederia foetida* Linn.	Rubiaceae	bath, decoction	herb	herb, fruit	eczema, skin infection, rheumatism syndrome, body pain
78	GZ1835	*Hedyotis mellii* Tutch.	Rubiaceae	bath, tea	herb	NT	detoxication, influenza, fever
79	GZ1830	*Uncaria rhynchophylla* (Miq.) Miq. ex Havil.	Rubiaceae	bath, decoction	hook-like stem and leaf	hook-like stem^a^, root	rheumatism syndrome, body pain
80	GZ1884	*Serissa japonica* (Thunb.) Thunb.	Rubiaceae	bath, tea, decoction	herb	herb	influenza, cough, tooth pain
81	GZ1861	*Lonicera japonica* Thunb.	Caprifoliaceae	bath, tea, decoction	stem and leaf	stem, flower bud^a^	detoxication, influenza, rheumatism syndrome,
82	GZ1872	*Ixeris polycephala* Cass. ex DC.	Asteraceae	bath, soup, tea, vegetable	herb	herb	detoxication, skin infection, furuncle
83	GZ1897	*Eclipta prostrata* (Linn.) Linn.	Asteraceae	bath, soup, vegetable, paste	herb	herb	dysentery, bleeding, furuncle
84	GZ1860	*Solidago decurrens* Lour.	Asteraceae	Bath, decoction	herb	herb^a^, root	skin disease, pharyngitis, pneumonia
85	GZ1848	*Aster pekinensis* (Hance) Kitag.	Asteraceae	Bath, decoction	herb	herb	fever, cough
86	GZ1849	*Crassocephalum crepidioides* (Benth.) S. Moore	Asteraceae	bath, soup, vegetable	herb	herb	detoxication, skin infection, indigestion
87	GZ1829	*Bidens pilosa* Linn.	Asteraceae	bath, tea	herb	herb	dysentery, hepatitis
88	GZ1822	*Duhaldea cappa* (Buch.-Ham. ex DC.) Anderb.	Asteraceae	bath, decoction	herb	NT	cold, cough, rheumatism syndrome
89	GZ1877	*Acorus gramineus* Sol. ex Aiton	Acoraceae	bath, decoction, tea	herb	rhizome^a^	skin itching, naupathia, dysentery
90	GZ1853	*Bromus japonicus* Thunb. ex Murr.	Poaceae	bath	herb	herb, seed	hidroschesis of kids
91	GZ1879	*Lophatherum gracile* Brongn.	Poaceae	bath, tea	leaf with stem	leaf with stem^a^	diuresis, heat, throat pain
92	GZ1874	*Zingiber officinale* Rosc.	Zingiberaceae	bath, tea	herb(overground part)	leaf and stem	skin disease, naupathia, indigestion
93	GZ1858	*Alpinia zerumbet* (Pers.) Burtt. et Smith	Zingiberaceae	bath, decoction	stem and leaf	rhizome, fruit	dysentery, indigestion
94	GZ1825	*Alpinia japonica* (Thunb.) Miq.	Zingiberaceae	bath	herb	rhizome, fruit	skin disease, rheumatism syndrome
95	GZ1826	*Alpinia japonica* (Thunb.) Miq.		bath, infusion, food seasoning	fruit	rhizome, fruit	skin disease, rheumatism syndrome, indigestion, tooth pain
96	GZ1876	*Smilax riparia* A. DC.	Smilacaceae	bath, infusion, decoction	root with rhizome	root with rhizome	skin itching, rheumatism syndrome, traumatic injury
97	GZ1868	*Smilax glabra* Roxb.	Smilacaceae	bath, infusion, decoction	rhizome	rhizome^a^	insecticide, syphilis, scabies

^*^ The herbal part in the Chinese Pharmacopoeia (CPH) was marked with “^a^”, while the others not marked are from the Chinese Materia Medica (CMM) and NT means no record in CPH nor CMM

### Chemical reagents

HPLC-grade n-hexane, ethyl acetate, methanol, and dimethyl sulfoxide (DMSO, molecular biology grade) were purchased from Sigma–Aldrich Co. (MO, USA). Sterile deionized water was produced by a Milli–Q Reagent Water System (MA, USA). Bacto™ peptone and yeast extract were from Lab M Ltd. (Lancashire, UK). Ciprofloxacin (LOT: 105M4195V, antibacterial control), chloramphenicol (LOT: 015 K0562, antibacterial control only for EF due to their resistance to ciprofloxacin) [37], (±)- miconazole nitrate salt (LOT: 085M4092V, antifungal control) and gossypol (LOT: 024M4030V, cytotoxic control) were all purchased from Sigma–Aldrich (MO, USA). For cell culture materials, fetal bovine serum (FBS), Dulbecco-Modified Eagle’s Medium-high glucose (DMEM), Hanks’s Balanced Salt Solution (HBSS), Phosphate-Buffered Saline (PBS), and penicillin-streptomycin solution (P/S) were from Sigma–Aldrich (MO, USA).

### Preparation of the extracts and antimicrobial assay

Hakka herbs were obtained directly from the aforesaid local markets, where most herbs were collected from the wild by local Hakkas and were in a fresh state, viz. whole plant, root, stem, bark, leaf, etc. Depending on the water content of the plant parts, they were dried at 40 °C for 12 to 60 h in an electro-thermostatic blast oven (Beijing Ever Bright Medical Treatment Instrument Co. Ltd., China), in an attempt to maintain volatile components if present. Then they were milled into a fine powder by a high-speed multifunctional grinder. All the materials were kept as dry as possible to prevent the growth of microorganisms. For each herb, at least 50 g dry medicinal crude preparation was purchased. One gram of each powder was extracted with pure water, methanol, ethyl acetate, and hexane, and after evaporation of the solvent, a working stock with a final concentration of 20 mg/mL was prepared in DMSO (solvent extracts) or water (water extracts) for antimicrobial tests against 20 pathogens as described previously [38, 39]. An herbal extract was defined as active if it inhibited microbial growth by more than 50%. Those extracts were selected for microdilution tests to determine their IC_50_ (half maximal inhibitory concentration for antimicrobial activity) [40].

### Cytotoxicity test

A resazurin-based cell viability assay with some modifications was used to investigate the cytotoxicity on human lung epithelial tumor cells (A549, obtained from Animal Physiology and Neurobiology Section, KU Leuven, Belgium), and non-tumoural human lung fibroblast cells (WI-26 VA4 from the European Collection of Authenticated Cell Cultures, Sigma Aldrich) [41]. All cell cultures were maintained in DMEM supplemented with 10% FBS and 100 I.U./mL antibiotic P/S solution. For the cytotoxicity test, 2 × 10^4^ cells were plated in each well of a multiwell-96 plate; calculation of the cell number was performed by a NucleoCounter (Chemometec, Denmark) [42]. Then, the stocks of Hakka herb extracts (20 mg/mL in DMSO or water) and a toxic reference compound (10 mM gossypol in DMSO) were diluted in HBSS and transferred to the 96-well plates at a final concentration of 0.5% DMSO. After 48 h, 10 μL resazurin solution (0.15 mg/mL in PBS) was added to each well; then the plate was incubated for 4 h while covered in aluminum foil. The absorbance was measured with a 550 nm excitation filter and a 590 nm emission filter in an automated multi-well fluorescence reader (FlexStation II, Molecular Devices, USA). The cytotoxicity was expressed as cell viability inhibition (%), which was calculated as 100% – (treated cells – background controls) / (DMSO controls – background controls) × 100%. An extract was defined as cytotoxic if it showed inhibition values against the tested cells of more than 50%. Those extracts were selected for a serial dilution test to determine their CC_50_ (cytotoxic concentration with 50% adverse effect).

### Validation and statistical analysis

All the tests described in this study were performed repeatedly to ensure reproducibility. The IC_50_ and CC_50_ values were calculated by GraphPad Prism 7.0 Software (San Diego, CA) [39]. The data of all 97 Hakka herbs were analyzed using the webtool ClustVis (https://biit.cs.ut.ee/clustvis/) to obtain hierarchical clustering heat maps [43]. To produce the heat maps, the parameters were set as follows: data import: upload file, detect delimiter, detect column and row annotations, no quotes, NA; pre-processing options: no transformation, maximum percentage for rows and columns, row centering, no scaling, Nipals PCA; PCA (no need); heat map (adjusted as the desired shape of heat map). To display the spectrum of each Hakka herbal extract, all the active ones were graphed in a radar plot using Excel, combining the antimicrobial and cytotoxic results.

## Results

The therapeutic effect of TCM against infectious diseases has drawn attention from international scientific research, and triggered a surge of publications on Chinese herbal medicine, also in top journals [44–48]. The multi-components, multi-targets, and extensive pharmacological activities of TCM [49] enable them to inhibit or kill microorganisms in a variety of ways, with less toxic - and side effects, and limited risk of developing drug resistance [50]. Following the TCM example, we chose to conduct ethnobotanical surveys of Hakka herbs which are considered locally as anti-infectious. From those plants, we then prepared extracts in different solvents, and tested these for antibacterial and antifungal activity, as well as cytotoxicity.

### Hakka traditional herbs and their medical application

**Fig. 2:**
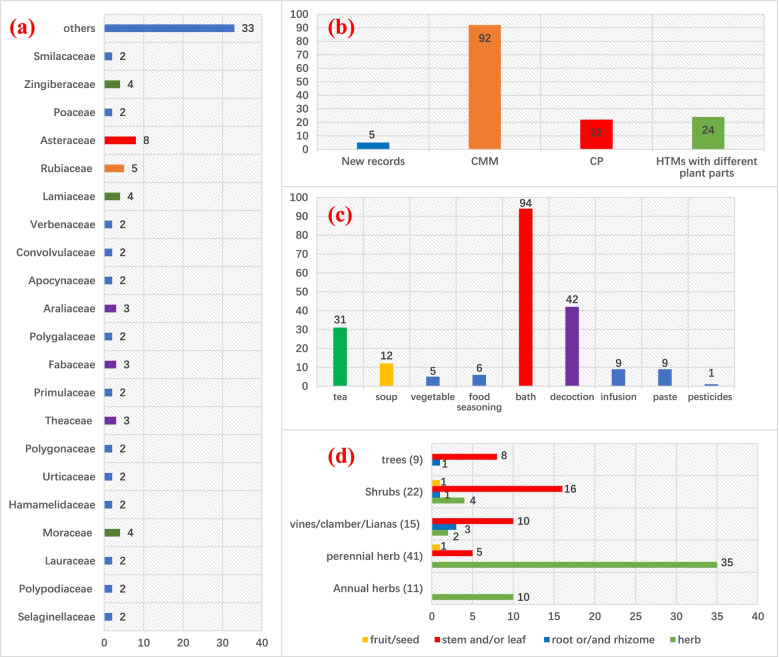
Analysis of HTM information. **a** Number of plant species per taxonomic family; **b** Record number in herbal books and HTM (CMM: Chinese Materia Medica, CPH: Chinese Pharmacopoeia 2020); **c** Administration modes of HTM; **d** Relationship between plant types and plant parts

After conducting the ethnobotanical survey of local markets, 97 herbs were selected and collected from these herbal markets (Table 1, Supplementary material 2). They originated from 93 plants comprising 84 genera in 62 families, of which 8 species are Pteridophytes and 85 are Angiosperms according to the Cronquist classification. In the latter group, there are 5 families with more than 4 members: Asteraceae (8), Rubiaceae (5), Moraceae (4), Lamiaceae (4), and Zingiberaceae (4) (Fig. 2a). Only 22 Hakka herbs can be found as TCM according to the Chinese Pharmacopoeia (CPH, the most authoritative source of TCM), like *Lycopodium japonicum*, *Picrasma quassioides*, *Lonicera japonica*, *Smilax glabra*, *Lophatherum gracile*, *Acorus gramineus*, *Solidago decurrens*, *Uncaria rhynchophylla*, etc. However, most of them are used differently by Hakkas (Table 1). Compared with the CPH and the most exhaustive book of Chinese medicinal herbs: Chinese Materia Medica (CMM, with 8980 species, including TCM and folk medicine), 5 Hakka plants are recorded here for the first time as traditional medicine (Fig. 2b): *Chimonanthus grammatus*, *Adinandra nitida*, *Eurya acuminatissima*, *Polygala angustifolia*, and *Hedyotis mellii*. *Adinandra nitida* for example is widely used, not only in Hakka medicine, but more for Hakka food coloring, e.g., to give cake made from rice flour- and appetite-inducing yellow color, called “Huangyuan Miguo”. Moreover, it prolongs food shelf life, indicating it probably has antimicrobial activity.

Given some uncertainty about the exact medical uses based on personal conversations, we focused first on the administration forms of Hakka plants, such as use in bathing, soup, tea, food additions, decoction, infusion, etc. The most common application was bathing (94 herbs), such as foot soak, body bathing, water fumigation, and washing (the herb is boiled in water, then used to fumigate the body, foot, or other body parts.). Most bathing plants were used for treating or preventing skin diseases (skin infection, itching, eczema, etc.), rheumatism syndrome, or against inflammation, such as *Lycopodium japonicum*, *Fissistigma oldhamii*, *Piper wallichii*, etc.

The second common use is for food, including tea (31 herbs), soup (12 herbs), vegetables (5 herbs), and food additives (6 herbs, e.g., food seasoning, food coloring, and food preservation). Hakka people were used to developing local plants for medicine/food purposes, which could help them fight hunger and disease at the same time. For instance, the stem of *Callerya dielsiana* was used to prepare soups not only because of the good taste, but also to prevent or treat diseases (blood deficiency, weak, irregular menstruation, etc.). The leaves of *Nekemias grossedentata* are used to prepare a locally famous tea, “Hakka Bai Cha”, which was widely used to increase body strength and cold resistance. With people yearning for a sense of nature and health, medicinal food became increasingly popular in China. HTM is a treasure trove for developing new types of medicinal food.

Herbal plaster or paste (9 herbs) for external use, a healing, cleansing application with antiseptic action, was also mainly used for skin diseases, such as *Elatostema involucratum* (skin ulcer, abscess, bleeding, viper bite) and *Saxifraga stolonifera* (eczema, inflammation, abscess, bleeding, pruritus). Decoctions and infusions are also widely used by Hakkas for medicinal purposes, for 42 and 9 herbs, respectively. They are used for a range of indications, like hepatitis, detumescence, pain, rheumatism syndrome, common cold, flu, etc. Figure 2c shows the different uses, with bathing dominating in all the medicinal applications.

### The plant parts used by Hakkas

For many Hakka herbs, there were discrepancies between the data we collected and published information, e.g., regarding the therapeutic application, administration forms, etc. Especially for the medicinal plant parts, there were 24 herbs used locally with different parts compared to previous documents (Fig. 2b). *Fissistigma oldhamii*, for instance, is recorded in TCM to only use its roots with the effect on rheumatism syndrome, promoting blood circulation, and relieving pain; Hakka people on the other hand use the leaves and stems for treating gynecology inflammation, in addition to rheumatism syndrome [16]. Also, the infected leaf of *Camellia oleifera*, locally called “Cha Er”, is used by Hakkas for food and drink (tea), while people normally use its seed, oil, root, leaf, flowers, or even oil-dregs. For *Lindera glauca*, the root, leaf, and fruit are documented as three different medicines in CMM, but the tender stem and leaves are typically used by Hakkas. There are also many medicinal plants, whose root and/or rhizome are recorded as their medical parts in CMM and CPH, but Hakkas use their aerial part instead, such as *Lindera glauca*, *Lindera glauca*, *Sabia japonica*, *Liquidambar formosana*, *Ficus formosana* f. *shimadai*, *Ficus simplicissima*, *Phytolacca americana*, *Urena lobata*, *Dalbergia hupeana*, *Phyllanthus glaucus*, *Cynanchum stauntonii*, *Alpinia zerumbet*, *Alpinia japonica*.

The plant parts used are closely related to the life cycle. Perennial herbs and shrubs form the largest percentage of Hakka herbs (Fig. 2d), accounting for 41 and 22 species, respectively. Regarding the parts used, annual and perennial herbs were generally used in their entirety (100 and 85%, respectively), while for woody plants, the (tender) stems and/or leaves dominated in HTM, including for vines (67%), shrubs (73%) and trees (89%). This suggests preferential use of the most easily obtained aerial parts (stems and/or leaves) rather than subterranean parts (root or rhizomes, requiring digging) or reproductive organs (flower, fruit, and seed, which are available only during limited periods). Hence, the aerial parts of Hakka plants were much more abundant in the local marketplaces. Out of a total of 97 plants, 90 had easily collected parts (stem, leaf, herb), while only other 7 plants were sold as root, rhizome, fruit, or seed, indicating that Hakka people prefer to use easily collected plant parts.

### Antimicrobial activity of Hakka herbs

**Fig. 3:**
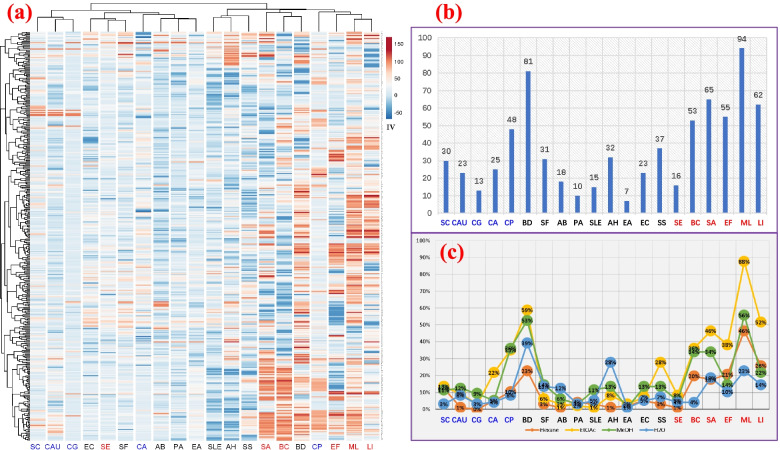
Antimicrobial activities of HTMs. **a** Heat map of inhibition values (IV) against 20 microbes (vertical axis are 388 extracts of 97 herbs; horizontal axis are the abbreviations of 20 human pathogens); **b** Number of active herbs (IV ≥ 50%); **c** Percentages of active extracts by solvent (hexane, EtOAc, MeOH, H_2_O). The abbreviations of microbes are marked in blue, black, and red for fungi, G^−^ bacteria, and G^+^ bacteria, respectively; Fungi: *Candida albicans* (CA), *Candida parapsilosis* (CP), *Candida auris* (CAU), *Candida glabrata* (CG) and *Saccharomyces cerevisiae* (SC); G^−^ bacteria: *Escherichia coli* (EC), *Pseudomonas aeruginosa* (PA), *Shigella sonnei* (SS), *Acinetobacter baumannii* (AB), *Enterobacter aerogenes* (EA), *Brevundimonas diminuta* (BD), *Shigella flexneri* (SF), *Salmonella enterica* subsp. *enterica* (SLE) and *Aeromonas hydrophila* (AH); G^+^ bacteria: *Staphylococcus aureus* (SA), *Staphylococcus epidermidis* (SE), *Micrococcus luteus* (ML), *Listeria innocua* (LI), *Enterococcus faecalis* (EF) and *Bacillus cereus* (BC)

According to the above results, most Hakka herbs are employed for infectious diseases via bathing and as medicine-food. Hence, we tested their extracts against a range of human pathogens, including 5 fungi, 9 Gram-positive, and 6 Gram-negative bacteria. Crude extracts were prepared with four solvents of different polarity, viz. hexane, ethyl acetate, methanol, and water. The bioactivity was detected by a broth microdilution assay, and the inhibition values are presented in Fig. 3a and Supplementary material 3. Encouragingly, 331extracts were active (IV ≥ 50%) against one or more microorganisms, accounting for 94% of all the tested extracts (4 extracts of 97 HTM plants each), suggesting that bathing with Hakka traditional herbs could be beneficial for protecting against infections. Active HTM extracts were selected for determining their IC_50_ (Supplementary material 4). All tests were repeated at least once, and IC_50_ was calculated by Prism 7. Most extracts had IC_50_ values between 200 and 1000 μg/mL, which is moderately active for crude plant extracts. Some HTMs showed stronger inhibition with IC_50_ values below 200 μg/mL (Table 2)*, *rendering them attractive for bioassay-guided purification and other further studies, such as *Selaginella tamariscina*, *Selaginella moellendorffii*, *Adiantum flabellulatum*, *Equisetum ramosissimum*, *Fissistigma oldhamii*’s root, *Lindera glauca*, *Cinnamomum jensenianum*, *Liquidambar formosana*, *Semiliquidambar cathayensis*’s root, *Maclura cochinchinensis*, *Ficus simplicissima*, *Persicaria chinensis* var. *paradoxa*, infected leaf of *Camellia oleifera*, *Adinandra nitida*, *Eurya acuminatissima*, *Hypericum japonicum*, *Lysimachia alfredii*, *Melilotus officinalis*, *Buxus sinica*, *Nekemias grossedentata*, *Turpinia arguta*, *Physalis angulata*, *Verbena officinalis*, *Origanum vulgare*, *Salvia prionitis*, *Buddleja lindleyana*, *Strobilanthes cusia*, *Crassocephalum crepidioides*, *Acorus gramineus*, *Alpinia japonica*’s fruit, and *Smilax glabra*.

**Table 2:** IC_50_ values (μg*/mL*) against 20 *microorganisms* of the most active extracts

Herb No. & Solvent	Plant name	Fungi					Gram-negative bacteria									Gram-positive bacteria					
		SC	CAU	CG	CA	CP	BD	SF	AB	PA	SLE	AH	EA	EC	SS	SE	BC	SA	EF	ML	LI
2–2	*S. tamariscina*	–	853	–	–	–	843	–	–	–	–	–	–	–	–	–	939	**88**	–	413	476
3–2	*S. moellendorffii*	–	797	–	–	–	597	–	–	–	–	–	–	–	–	–	992	374	–	**30**	**73**
5–4	*A. flabellulatum*	–	–	–	–	–	–	826	–	–	–	–	–	–	–	–	–	**38**	–	–	–
6–2	*S. hastata*	–	–	–	–	–	–	–	–	–	–	–	–	–	–	–	733	**135**	–	–	–
8–2	*E. ramosissimum*	–	–	–	–	–	978	–	–	–	–	929	–	–	–	–	–	244	974	**106**	431
8–3		–	–	–	–	–	–	–	–	–	–	853	–	–	–	–	–	**182**	–	345	395
9–3	*F. oldhamii's root*	–	–	–	864	–	**143**	309	–	492	763	826	398	530	299	975	–	982	–	**138**	–
13–3	*L. glauca*	–	–	–	894	–	264	–	–	–	–	–	–	383	–	–	–	**144**	–	984	–
14–2	*C. jensenianum*	308	865	–	–	797	995	–	–	–	–	–	–	–	950	–	892	**150**	242	284	–
19–2	*L. formosana*	–	–	–	–	–	–	–	–	–	–	–	–	–	–	–	–	278	**191**	310	847
21–3	*S. cathayensis' root*	–	504	603	–	927	–	–	–	–	–	–	–	–	–	–	403	403	**104**	**103**	–
25–1	*M. cochinchinensis*	–	–	–	–	–	–	–	–	–	–	–	–	–	–	–	435	**166**	**138**	975	927
25–2		–	–	972	–	–	**144**	595	967	–	–	–	879	241	–	634	**67**	**45**	**11**	947	980
25–3		924	–	–	–	947	976	–	–	–	–	–	–	–	–	–	–	**38**	–	964	–
25–4		–	–	–	–	–	–	–	–	–	–	–	–	–	–	–	–	**12**	–	–	–
26–4	*F. simplicissima*	–	–	–	–	–	982	–	–	–	–	–	–	–	–	–	–	–	**190**	974	946
31–2	*P. chinensis* var. *paradoxa*	–	–	–	–	–	–	–	–	–	–	–	–	–	–	–	–	–	–	**105**	835
32–2	*infected leaf of C.oleifera*	–	–	–	–	–	803	–	–	–	–	–	–	–	–	–	256	**71**	569	299	471
33–2	*A. nitida*	–	–	–	–	–	873	–	–	–	–	–	–	–	–	–	–	–	–	**106**	695
33–3		–	–	–	–	876	201	306	–	–	–	205	–	307	874	–	–	**110**	–	–	–
34–4	*E. acuminatissima*	–	–	–	998	–	–	880	–	–	–	975	–	–	460	–	–	–	**148**	870	786
35–2	*H. japonicum*	**121**	985	–	–	995	424	–	–	–	–	925	–	–	–	–	–	**92**	958	**191**	841
39–2	*L. alfredii*	–	–	–	–	–	355	–	–	–	–	–	–	–	–	–	–	–	240	**171**	396
45–2	*M. officinalis*	–	–	–	–	943	965	–	–	–	–	–	–	–	–	–	757	**136**	862	665	–
50–3	*B. sinica*	–	–	–	–	–	275	497	–	–	274	893	–	796	–	–	–	**185**	–	264	–
52–4	*N. grossedentata*	–	–	–	–	–	**104**	–	–	–	–	296	–	–	–	–	–	982	–	598	–
57–2	*T. arguta*	–	–	–	985	–	590	–	–	–	–	–	–	–	–	–	–	564	–	**164**	295
57–3		–	–	499	–	764	387	297	296	–	–	296	–	–	–	–	–	–	–	286	**185**
64–3	*P. angulata*	**102**	975	–	–	–	473	–	–	–	–	–	–	–	–	–	–	–	–	955	–
67–1	*V. officinalis*	–	–	–	–	–	938	–	–	–	–	–	–	–	–	–	**162**	–	–	–	–
69–2	*O. vulgare*	–	–	–	895	–	–	–	–	–	–	–	–	–	–	–	397	–	**109**	234	**136**
70–2	*S. prionitis*	–	–	–	736	985	**199**	–	–	–	–	–	–	467	498	369	–	–	499	**176**	**117**
72–4	*M. scabra*	–	–	–	–	–	355	–	–	–	–	–	–	–	–	–	–	–	**127**	422	565
73–2	*B. lindleyana*	973	–	–	885	–	974	–	–	–	–	–	–	–	–	–	–	985	–	**194**	399
75–2	*S. cusia*	–	–	–	895	987	**158**	–	–	–	–	–	–	–	764	–	–	–	298	376	–
86–1	*C. crepidioides*	–	–	–	–	–	792	–	–	–	–	–	–	–	–	–	–	–	–	834	–
86–3		–	–	–	–	995	982	–	–	–	–	–	–	–	–	–	–	–	–	264	**182**
86–4		–	–	–	–	–	786	786	–	–	509	992	–	–	753	–	–	–	539	**184**	530
89–1	*A. gramineus*	–	–	–	–	–	–	–	–	–	–	–	–	–	–	–	**185**	251	–	–	–
95–1	*A. japonica's fruit*	–	829	–	–	–	–	–	–	–	–	–	–	–	–	–	–	–	639	**136**	–
97–3	*S. glabra*	798	–	–	–	786	764	–	–	–	–	–	–	–	–	–	997	–	540	**181**	932
P	Positive control^a^	0.01	0.10	0.12	0.01	0.13	2.26	0.02	0.17	0.02	0.01	0.01	0.04	0.02	0.02	0.49	0.02	0.28	9.44	2.11	0.59

No.1 to 97: Hakka herbs listed in Table 1; Solvent (−1 to −4): hexane, ethyl acetate, methanol, water;

IC_50_ values less than 200 μg/mL were marked in bold;

^a^ Positive control: miconazole for fungi, chloramphenicol for EF, ciprofloxacin for other bacteria

The inhibition values of all 388 Hakka herbal extracts against human pathogens were clustered and presented in a heat map (Fig. 3a). Hakka herbs showed much more often activity against G^+^ bacteria than G^−^ bacteria and fungi. Figure 3b shows the number of active herbs against each microbe; few were active against *E. coli* (7 herbs), *Pseudomonas aeruginosa* (12), and *Candida glabrata* (15), implying that it is difficult to find activities against certain fungi and G^−^ bacteria, a pattern previously reported by others [51, 52]. The reason could be that the cells of G^−^ bacteria and some fungi have a multilayer outer membrane structure, preventing many antibiotics from passing through [53, 54]. The twenty microbes were ordered in clusters in the heat map, matched with their original categories for fungi, G^+^ and G^−^ bacteria, except for *Staphylococcus epidermidis*, *Brevundimonas diminuta*, *Candida albicans*, and *Candida parapsilosis.*

Most activities were detected against G^+^ bacteria, such as *Micrococcus luteus* (97% of herbs), *Staphylococcus aureus* (67%), *Listeria innocua* (64%), *Enterococcus faecalis* (57%), and *Bacillus cereus* (55%). However, far fewer extracts were active against *S. epidermidis* (16%), suggesting different mechanisms of action in the same *Staphylococcus* genus, and that the *S. epidermidis* used in this study (a biofilm-forming strain) has higher resistance against botanical extracts. On the other hand, most herbs did not show much activity against G^−^ bacteria, except *Brevundimonas diminuta* (84%), which is an emerging global opportunistic pathogen, that causes bloodstream infections [55, 56]. The other major activities were against *Shigella sonnei* (38%), *Aeromonas hydrophila* (33%), and *Shigella flexneri* (32%). For fungi, the herbs were most active against *C. parapsilosis* (49%), which increasingly causes infections since the turn of the century: fungemia, endocarditis, meningitis, skin infections, etc. [57–59]. HTMs also showed some inhibition of *Saccharomyces cerevisiae* (31%), *C. albicans* (26%), *C. auris* (24%), and *C. glabrata* (13%).

Furthermore, the bioactivity data of all 388 extracts from 97 HTMs indicate a relationship between extracting solvents and bioactivities; ethyl acetate and methanol extracts are more often active than hexane or water extracts. For *Micrococcus luteus* (Fig. 3c), 88% of ethyl acetate extracts showed good antimicrobial activity with IV ≥ 50%, as did 56% of methanol extracts, compared to 46% of hexane extracts and 23% of water extracts. It is clear that ethyl acetate and methanol extracts inhibited more microorganisms than hexane or water extracts. The reason may be the bigger extracting capacity of the first two solvents, which usually dissolve more types of chemicals than hexane or water, and thus have more chance to contain bioactive chemicals [60]. However, methanol extracts showed less antimicrobial activity in many cases than ethyl acetate extracts. The reason may be that methanol has higher power to dissolve materials and its extracts generally were more complex [61], therefore, contain at the same overall concentration, more chemicals, which will lead to a lower concentration of the active compounds.

Notably, the water extracts were more active than other solvent extracts for *Aeromonas hydrophila*, whose name suggests a link with water (*hydrophila* means water-loving). This bacterium can survive in both salt and fresh water, and it has been detected in many kinds of food. It causes various human infections, including wound infections, septicemia, pneumonia, meningitis, and gastroenteritis, due to the most potent virulence factors of Aeromonas species via type-II and the recently proposed type-III secretion system [62–65]. The reason why water extracts showed more activity against *A. hydrophila* may be that water-soluble chemicals (such as plant polyphenols, peptides or their derivates, etc.) prevent the toxin secretion or their reaction with host cells; this hypothesis is worth studying further [66–70].

### Cytotoxicity of Hakka herbs

**Fig. 4:**
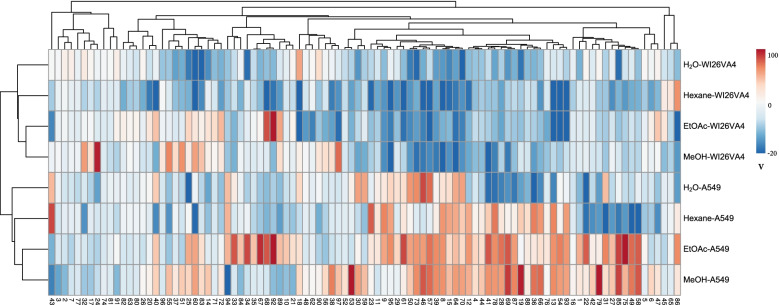
Heat map of cytotoxicity of different extracts of 97 HTMs against A549 and WI-26 VA4 cell lines. V: Percentage cell viability inhibition (%); Horizontal axis shows 97 Hakka herbs; the vertical axis shows extracts in 4 solvents

All 388 extracts of the 97 herbs were tested for cytotoxicity against a human lung epithelial cancerous cell line (A549) and a noncancerous lung cell line (WI-26 VA4). The data were analyzed by a heat map clustering (Fig. 4) and listed in Supplementary material 5. For the active extracts, CC_50_ tests were performed against cancer and non-cancer lung cells (Table 3). In total, there were 60 herbs active against A549 and WI-26 VA4 cells. Three HTMs (*Ficus formosana f. shimadai*, *Polygala japonica,* and *Eclipta prostrata*) only inhibited WI-26 VA4, 12 were active against both, and 45 only against A549. The last group, showing inhibition only for cancer cells but not against noncancerous cells, may have potential in oncology, and includes *Fissistigma oldhamii*, *Uncaria rhynchophylla*, *Strobilanthes cusia*, *Aster pekinensis*, *Picrasma quassioides*, *Liquidambar formosana*, *Verbena officinalis*, *Adinandra nitida*, *Eurya acuminatissima*, *Agrimonia pilosa*, etc. Remarkably, for *Fissistigma oldhamii*, the root and leaf showed strong inhibition of A549 cells, but not its stem, suggesting significant differences in the chemical composition of different plant parts [16]. On the other hand, for *Picrasma quassioides*, both the stem and leaf extracts inhibited A549 growth.

**Table 3:** Cytotoxicity (CC_50_ in μg/mL) of selected plant extracts

No.	Plant name	CC^50*^	
		A549	WI26-VA4
1	*L. japonicum*	67.2-E	–
8	*E. ramosissimum*	90.2-H, 82.1-E, 54.3-M, 97.5-W	–
9	*F. oldhamii*’s root	45.4-H, 54.4-E, 77.6-W	–
11	*F. oldhamii*’s leaf	88.6-H, 85.3-E, 94.1-W	–
12	*C. grammatus*	69.7-H, 65.9-E, 67.4-M	–
13	*L. glauca*	73.1-H, 93.6-E, 85.1-M	–
14	*C. jensenianum*	95.8-M	–
15	*S. chinensis*	83.9-H, 96.1-E, 74.8-M 84.9-W	–
18	*S. japonica*	98.1-E, 75.1-M 85.5-W	88.5-M, 63.8-W
19	*L. formosana*	94.6-H, 53.1-M	–
22	*D. macropodum*	83.9-E	–
23	*F. pumila*	59.7-H	–
24	*F. formosana* f. *shimadai*	–	57.3-M
25	*M. cochinchinensis*	89.5-H, 63.5-M	60.1-E, 76.9-M
28	*E. involucratum*	86.2-E, 95.1-M	–
30	*P. chinense*	84.2-M, 78.2-W	–
31	*P. chinensis* var. *paradoxa*	76.5-E, 99.8-W	–
33	*A. nitida*	87.2-E	–
34	*E. acuminatissima*	89.6-E	–
36	*C. crenata*	89.4-H, 87.5-M	–
38	*P. stenoptera*	94.9-M	–
39	*L. alfredii*	84.7-E, 62.7-M	–
40	*L. fortunei*	78.6-E, 74.1-M, 72.1-W	88.6-E
41	*D. febrifuga*	82.9-E, 35.3-M	–
43	*A. pilosa*	65.9-H	–
44	*C. dielsiana*	99.3-E, 76.4-M	–
46	*D. hupeana*	94.7-E, 78.5-M, 72.5-W	–
47	*H. micrantha*	35.8-M	80.4-E
49	*S. parasitica*	78.9-E, 69.7-M	–
50	*B. sinica*	74.5-E, 89.5-M, 85.1-W	–
51	*P. glaucus*	95.8-M	81.4-M
53	*P. quassioides*’s stem	45.7-M	–
54	*P. quassioides*’s leaf	70.5-E, 73.9-M	–
55	*P. japonica*	–	91.4-M
57	*T. arguta*	48.6-E, 74.2-M, 49.2-W	–
58	*Z. simulans*	89.4-E, 69.4-M	–
60	*H. heptaphyllum*	53.8-E, 58.1-M, 95.2-W	79.8-E, 95.6-M
61	*F. japonica*	95.3-E	–
64	*P. angulata*	91.3-H, 84.2-E, 97.8-M, 97.6-W	–
66	*E. alsinoides*	87.0-H, 86.3-M	–
67	*V. officinalis*	33.1-E	–
68	*V. negundo* var. *cannabifolia*	58.6-E	54.7-E
69	*O. vulgare*	88.9-E, 77.3-M	–
70	*S. prionitis*	86.7-H, 84.8-E, 74.9-M, 89.2-W	–
72	*M. scabra*	96.8-M	96.4-E, 91.5-M
73	*B. lindleyana*	38.1-E, 42.8-M, 49.6-W	–
75	*S. cusia*	48.6-E, 97.5-M	–
78	*H. mellii*	79.6-H, 89.6-E, 97.2-M	–
79	*U. rhynchophylla*	92.5-M	–
83	*E. prostrata*	–	98.1-M
85	*A. pekinensis*	60.8-E, 76.9-M	–
86	*C. crepidioides*	73.9-E	84.9-H
87	*B. pilosa*	95.5-E, 93.5-M	–
88	*D. cappa*	92.8-H	–
89	*A. gramineus*	98.5-E	85.9-E, 99.2-M
92	*Z. officinale*	47.5-E	47.6-E
93	*A. zerumbet*	87.4-H, 73.8-E, 65.9-M	–
94	*A. japonica*’s herb	74.7-H, 65.7-E, 89.4-W	–
95	*A. japonica*’s fruit	95.8-H, 89.3-E	–
97	*S. glabra*	92.4-M	88.5-M
P	gossypol	15.6	7.7

*P* Positive control (gossypol)

^* ^Extracted in different solvents: hexane (H), ethyl acetate (E), methanol (M), and water (W)

In Fig. 4, the activities were clearly clustered into two groups corresponding to the two cell lines, and showed also differences between the four solvents. For A549 cells, the percentages of active extracts (cell viability inhibition ≥50%) in hexane, ethyl acetate, methanol, and water were 20, 43, 40, and 16%, respectively, while only 1, 8, 10, and 1% were active against WI26VA4 cells. Thus, extracts were much more often active against canceraous than normal lung cells, and methanol, as well as ethyl acetate extracts, showed more often activity, similar to our antimicrobial tests. The aqueous extracts rarely show toxicity for normal cells, which was also seen in eight Indian plants [71], and this supports their safety for use in bathing or as tea.

Furthermore, the relationship between cytotoxicity and extract properties was analyzed. Distinguished by application, 83% of soup herbs showed cytotoxic activity, compared to 80% of vegetable herbs, 100% of food seasoning herbs, 64% of bath herbs, and 50% of tea and paste herbs. But the percentage of herbs active only against A549 was 83% of food seasoning herbs, 58% of soup herbs, 48% of bath herbs, 40% of vegetables, 38% of paste, and 37% of tea herbs. Furthermore, the relationship between plant type and cytotoxicity was analyzed; of all HTM, 64% of extracts from tree materials were active against A549 cancer cells, compared to 76% of shrubs, 47% of climbers, 54% of perennial herbs, and 56% of annual herbs, which can be summarized as 54% of herbaceous plants, and 63% for woody plants. A previous study of Brazilian plants in a semi-arid region showed there were no significant differences between woody and herbaceous plants [72], while another study in an arid ecosystem from America implied that extracts of perennial plants exhibited better in vitro activity against cancer cells than extracts from other types of plants [73]; both results differ somewhat from Hakka herbs. The reason may be that all Hakka herbs were from Ganzhou, which has a humid subtropical monsoon climate.

### The activity spectrum of Hakka herbs

**Fig. 5:**
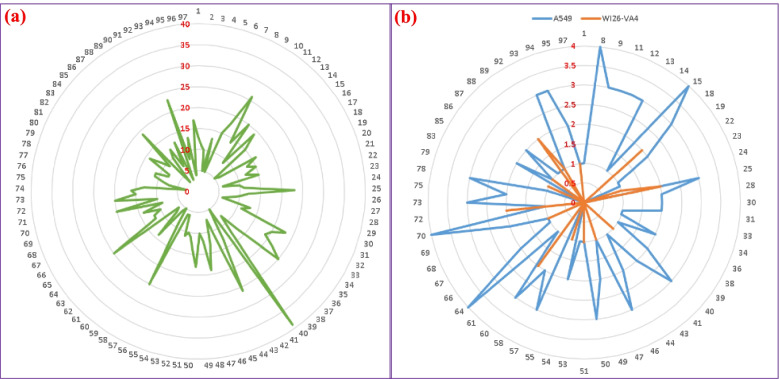
The number of active extracts (marked in red) of HTMs (herb number as in Table 1) against 20 microbes (**a**) and human cell lines (**b**)

Based on their antimicrobial and cytotoxic tests, all the active herbs were graphed on a radar plot (Fig. 5). Each of the 97 herbs showed at least 3 antimicrobial activities, such as *Mussaenda pubescens*, which inhibited the growth of *Micrococcus luteus* (hexane and ethyl acetate extract) and *Shigella sonnei* (ethyl acetate extract). The broadest spectrum (≥ 20 activities in each of 4 extracts over 20 microbes) was from 14 herbs: *Lysimachia fortunei*, *Fissistigma oldhamii's* root, *Agrimonia pilosa*, *Corchoropsis crenata*, *Turpinia arguta*, *Physalis angulata*, *Maclura cochinchinensis*, infected leaf of *Camellia oleifera*, *Alpinia zerumbet*, *Hypericum japonicum*, *Fissistigma oldhamii's* leaf, *Adinandra nitida*, *Salvia prionitis*, *Mosla scabra*.

On the other hand, the cytotoxic tests found 15 anticancer herbs with optimum activity (defined as ≥3 active extracts against tumor cell, and no activity against the noncancerous cell): *Equisetum ramosissimum*, *Saururus chinensis*, *Physalis angulata*, *Salvia prionitis*, *Fissistigma oldhamii* root, *Fissistigma oldhamii* leaf, *Chimonanthus grammatus*, *Lindera glauca*, *Dalbergia hupeana*, *Buxus sinica*, *Turpinia arguta*, *Buddleja lindleyana*, *Hedyotis mellii*, *Alpinia zerumbet*, and *Alpinia japonica* leaf.

As far as we know, most bioactivities for many herbs in this study are reported here for the first time. Based on the two kinds of tests (antimicrobial and cytotoxicity), the following plants were considered as the most promising for further study, with quite limited reports on their phytopharmacology and phytochemistry: *Lycopodium japonicum*, *Selliguea hastata*, *Loxogramme salicifolia*, leaf or stem of *Fissistigma oldhamii*, *Chimonanthus grammatus*, *Asarum caudigerum*, *Semiliquidambar cathayensis*, *Ficus formosana* f. *shimadai*, *Elatostema involucratum*, *Persicaria chinensis* var. *paradoxa*, *Eurya acuminatissima*, infected leaf of *Camellia oleifera*, *Corchoropsis crenata*, *Lysimachia alfredii*, *Haloragis micrantha*, *Polygala angustifolia*, *Hedyotis mellii*, etc.

## Discussion

The present survey is the first comprehensive report of Hakka traditional herbs in the cradle area, Ganzhou. It documented 97 Hakka herbs from 93 plants belonging to 84 genera in 62 families. Only 22 Hakka herbs were recorded as TCM according to the Chinese Pharmacopoeia 2020. Compared with the most comprehensive book on medicinal plants: the Chinese Materia Medica, 24 Hakka herbs were reported here as using different plant parts for medicine. Moreover, 5 Hakka herbs are recorded here for the first time as traditional medicine. Given the loss of traditional knowledge due to the fast progress of urbanization, this study contributes to the conservation of scientifically and culturally valuable knowledge of Hakka traditional herbs [17, 74].

Notably, during their identification and literature study, many herbal records could be easily missed because some Latin taxonomic names have been revised several times, such as *Duhaldea cappa* which was earlier classified as *Inula,* and many more research reports can be found under the former name *Inula cappa*; the same goes for *Callerya dielsiana*, *Nekemias grossedentata*, *Acorus gramineus*, etc. Hence, both current and earlier plant names were compared and checked systematically with flora of China, flora of Jiangxi, and the plant list database (http://www.theplantlist.org/).

For their medicinal usage, bathing herbs constituted the overwhelming majority (97%); they are used for foot soak, body bathing, water fumigation, and washing to treat or prevent skin diseases. The second common usage was for medicine-food purposes, including 31 tea herbs, 12 soup herbs, 5 vegetables, and 6 food additives. Hakkas are used to collecting the aerial plant parts as medicine, which play crucial roles in their daily life, especially during the period between spring and summer, when epidemic diseases easily break out. In south China, the temperature and humidity of the Hakka area keep increasing during these seasons, and microbes become more and more active, which causes more infections, including human skin diseases, respiratory disease, rheumatism syndrome, etc. [75–77]. Therefore, most Hakka herbs were employed for infectious diseases, which suggests their potential as anti-infectious agents. According to their antimicrobial tests, encouragingly, all 97 herbs are active (IV ≥ 50%) and can inhibit at least 3 different microbes, suggesting HTMs could protect against infections. Moreover, 57 herbs are active against a human tumor cell, whereas only 15 herbs show toxicity against non-tumor cells, which could be beneficial for oncology.

## Conclusion

This study is a starting point for the discovery of anti-infectious agents from traditional botanical medicine used by Hakkas in Ganzhou, China. The pharmacology and phytochemistry of many of these plants have not been reported so far, offering attractive perspectives for further research. Besides, this research provided a way to access and analyze the activity of traditional herbs, and can guide the selection of active ones. Further work is necessary to purify and identify the bioactive compounds, especially for the most promising or newly recorded Hakka herbs.

## Supplementary Information

**Additional file 1.**

**Additional file 2.**

**Additional file 3.**

**Additional file 4.**

**Additional file 5.**

## Data Availability

All data generated or analysed during this study are included in this published article and its supplementary information files.

## Abbreviations

AB: *Acinetobacter baumannii* (RUH134)
AH: *Aeromonas hydrophila* (ATCC 7966)
BC: *Bacillus cereus* (LMG9610)
BD: *Brevundimonas diminuta* (from Prof. Rob Lavigne at KU Leuven)
CA: *Candida albicans* (SC 5314)
CAU: *Candida auris* (OS299)
CC_50_: cytotoxic concentration with 50% adverse effect
CG: *Candida glabrata* (ATCC 2001)
CMM: Chinese Materia Medica
CP: *Candida parapsilosis* (ATCC 22019)
CPH: Chinese Pharmacopoeia
EA: *Enterobacter aerogenes* (ATCC 13048)
EC: *Escherichia coli* (ATCC 47076)
EF: *Enterococcus faecalis* (HC-1909-5)
G^+^: Gram-positive
G^−^: Gram-negative
HTM: Hakka traditional medicine
IC_50_: half-maximal inhibitory concentration for antimicrobial activity
LI: *Listeria innocua* (LMG 11387)
ML: *Micrococcus luteus* (DPMB 3)
PA: *Pseudomonas aeruginosa* (PAO1)
SA: *Staphylococcus aureus* (ATCC6538, Rosenbach)
SC: *Saccharomyces cerevisiae* (ATCC 7754)
SLE: *Salmonella enterica* subsp. *enterica* (ATCC 13076)
SE: *Staphylococcus epidermidis* (ATCC 1457)
SF: *Shigella flexneri* (LMG 10472)
SS: *Shigella sonnei* (LMG 10473)
TCM: Traditional Chinese medicine
WHO: The World Health Organization
