# Supplementary material for: Ethnobotanical study of Hakka traditional medicine in Ganzhou, China and their antibacterial, antifungal, and cytotoxic assessments
Source: BMC Complement Med Ther. 2022 Sep 19;22:244. doi: 10.1186/s12906-022-03712-z (PMC9484230; doi:10.1186/s12906-022-03712-z)
Supplement: Supplementary file 1 — Additional file 1. [file 12906_2022_3712_MOESM1_ESM.pdf]

Supplementary Material 1 - Survey form of data and sample collection for local herbal markets in Ganzhou, China

1. Sample ID:

2. Place (District, Village) :

3. Local name

4. Scientific name and type of original plant:

5. Medicinal parts used, and method of collection:

6. Forms of administration:

7. Medicinal use:

8. Pictures of the collected plant (including the main characteristics for species identification):

9. Type of the respondents: ☐ Herb seller
☐ Herb buyer
☐ Traditional indigenous doctor
☐ Others

Reporter:

Name:

Date:
