# Supplementary material for: Ethnobotanical study of Hakka traditional medicine in Ganzhou, China and their antibacterial, antifungal, and cytotoxic assessments
Source: BMC Complement Med Ther. 2022 Sep 19;22:244. doi: 10.1186/s12906-022-03712-z (PMC9484230; doi:10.1186/s12906-022-03712-z)
Supplement: Supplementary file 2 — Additional file 2. [file 12906_2022_3712_MOESM2_ESM.pdf]

Supplementary Material 2 - HTMs collected from the local herbal markets in Ganzhou, China

(Photoed by authors)

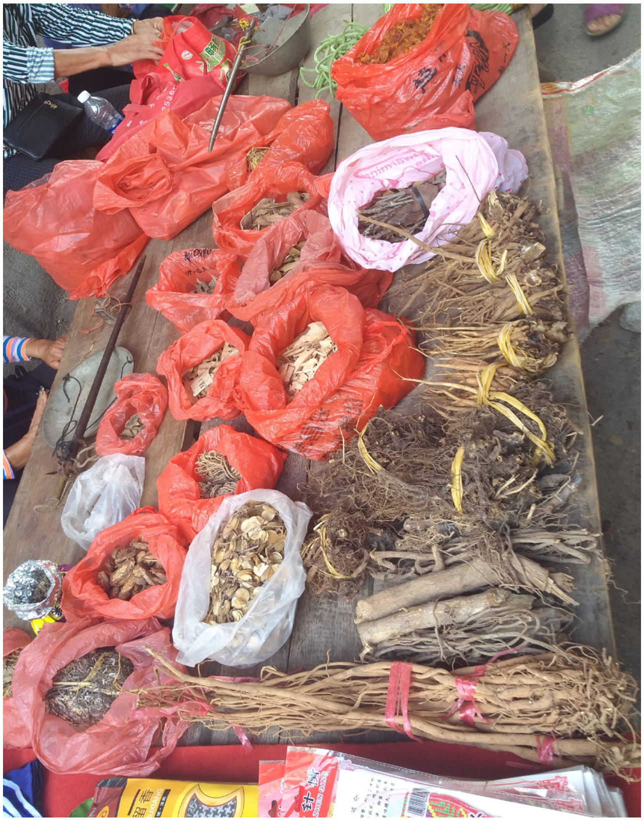

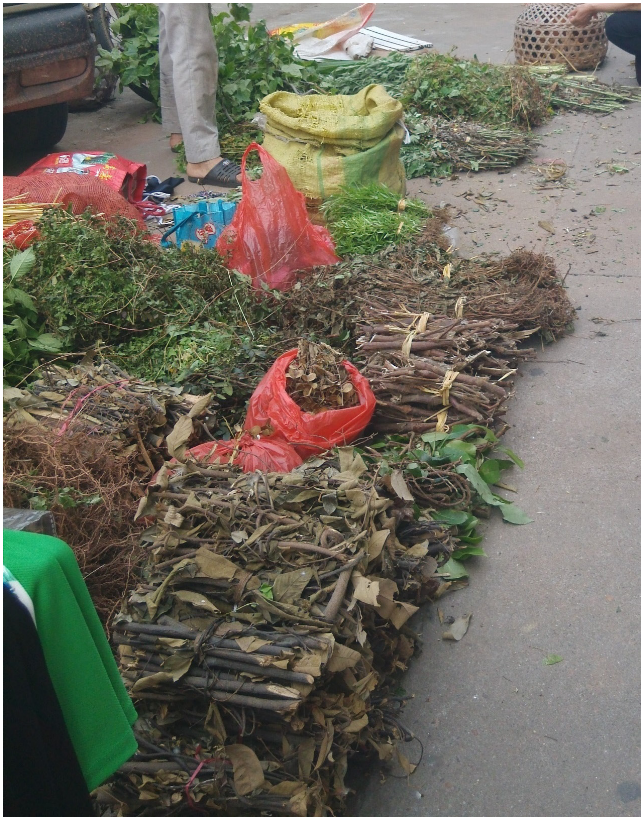

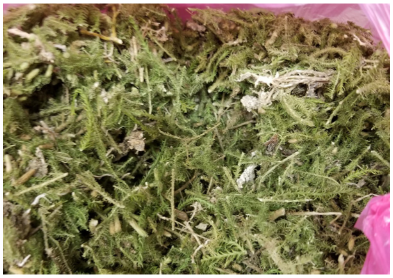

1. *Lycopodium japonicum.*

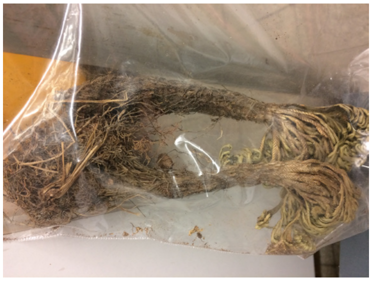

2. *Selaginella tamariscina*

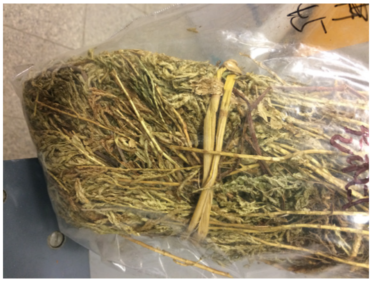

3. *Selaginella moellendorffii*

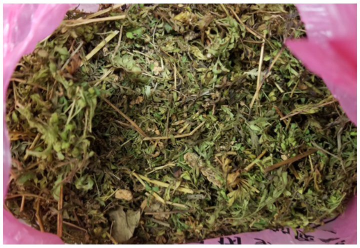

4. *Odontosoria chinensis*

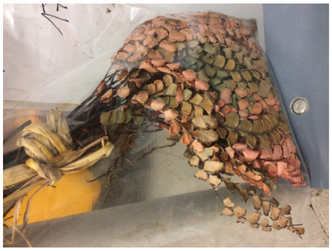

5. *Adiantum flabellulatum*

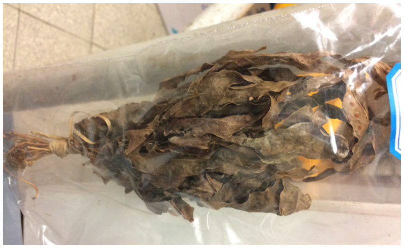

6. *Selliguea hastata*

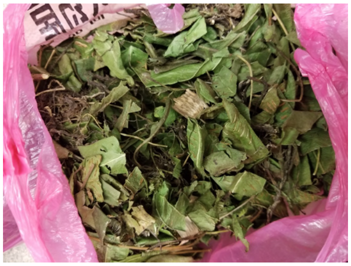

7. *Loxogramme salicifolia*

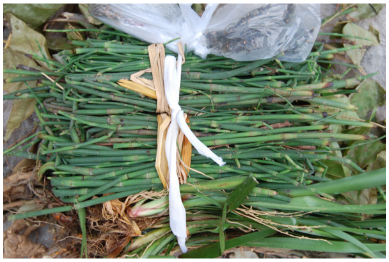

8. *Equisetum ramosissimum*

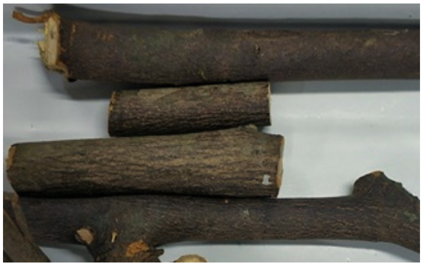

9. Root of *Fissistigma oldhamii*

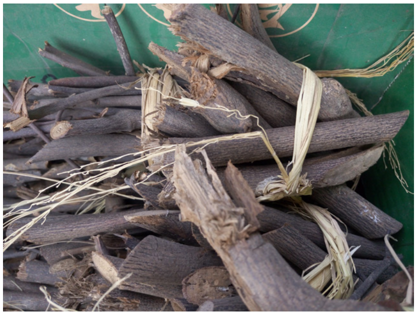

10. Stem of *Fissistigma oldhamii*

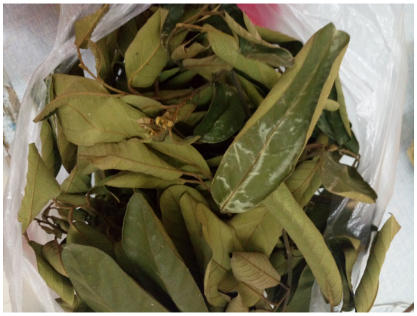

11. Leaf of *Fissistigma oldhamii*

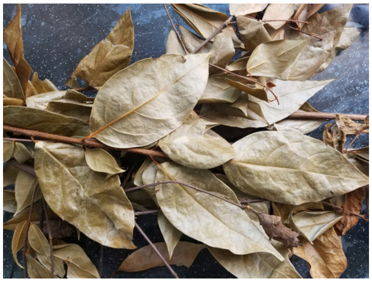

12. *Chimonanthus grammatus*

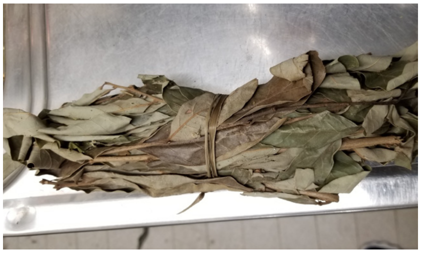

13. *Lindera glauca*

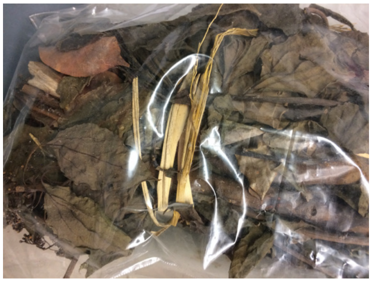

14. *Cinnamomum jensenianum*

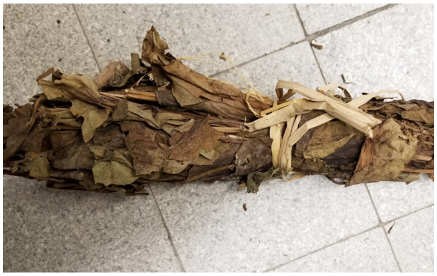

15. *Saururus chinensis*

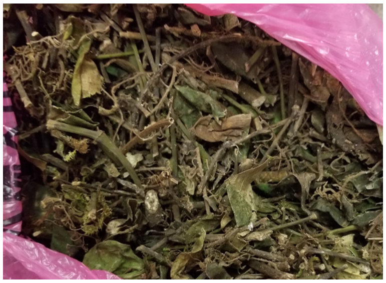

16. *Piper wallichii*

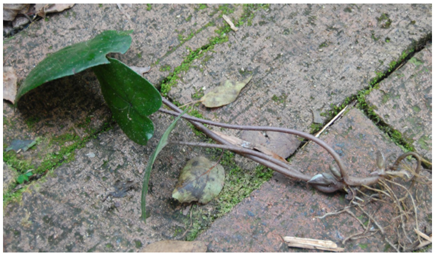

17. *Asarum caudigerum*

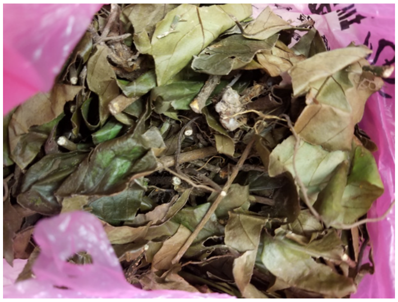

18. *Sabia japonica*

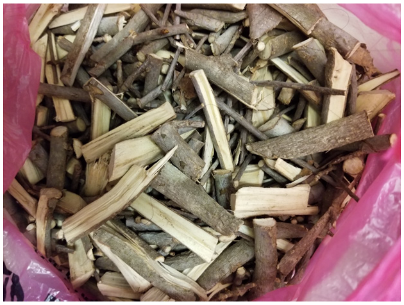

19. *Liquidambar formosana*

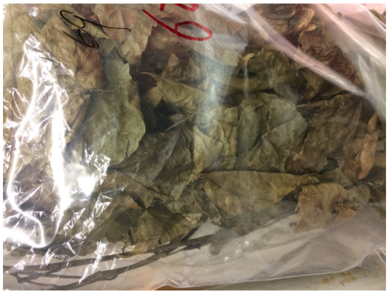

20. Tender stem and leaf of *Semiliquidambar cathayensis*

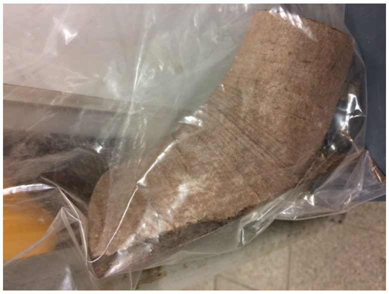

21. Root of *Semiliquidambar cathayensis*

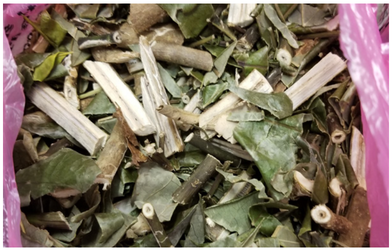

22. *Daphniphyllum macropodum*

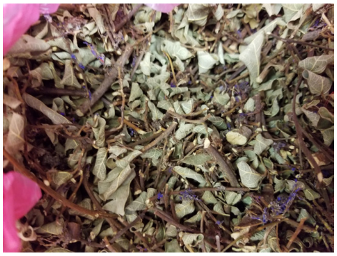

23. *Ficus pumila*

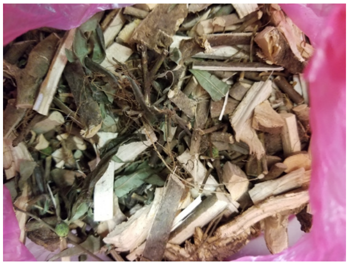

24. *Ficus formosana* f. *shimadai*

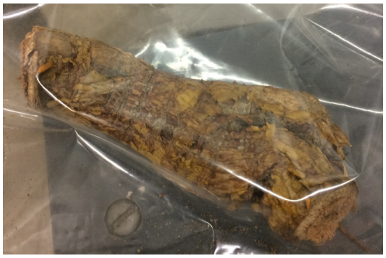

25. *Maclura cochinchinensis*

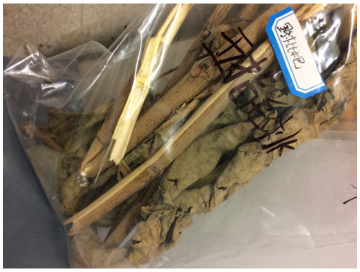

26. *Ficus simplicissima*

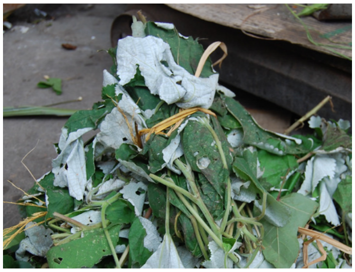

27. *Boehmeria nivea*

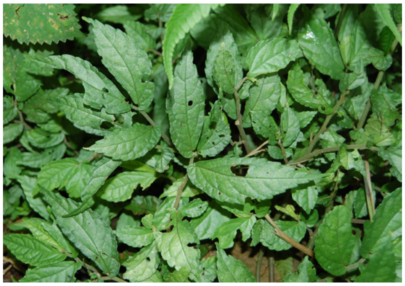

28. *Elatostema involucratum*

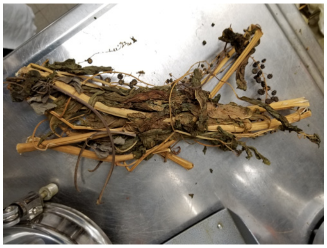

29. *Phytolacca americana*

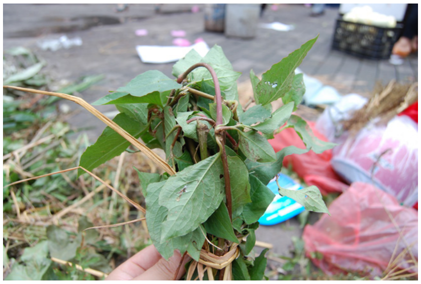

30. *Odontosoria chinensis*

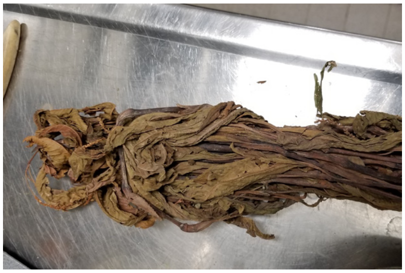

31. *Persicaria chinensis var. paradoxa*

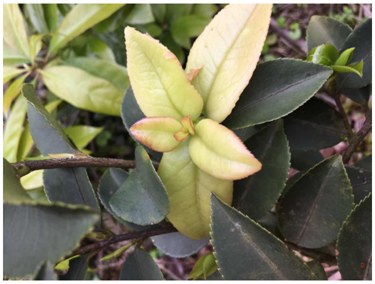

32. infected leaf of *Camellia oleifera*

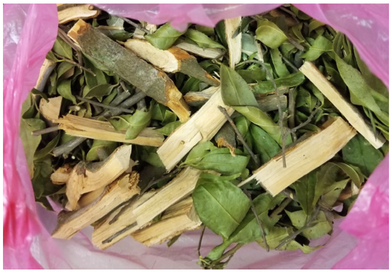

33. *Adinandra nitida*

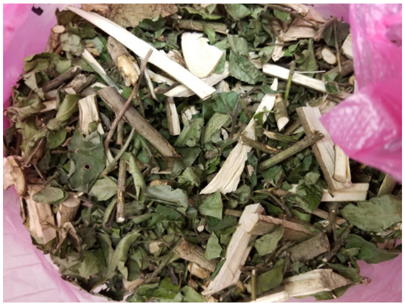

34. *Eurya acuminatissima*

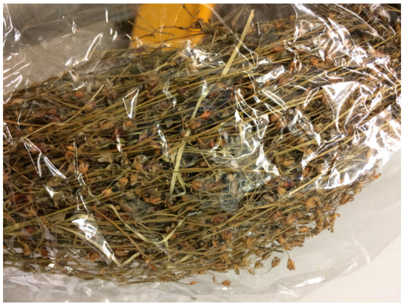

35. *Hypericum japonicum*

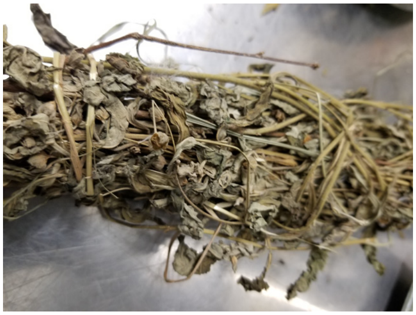

36. *Corchoropsis crenata*

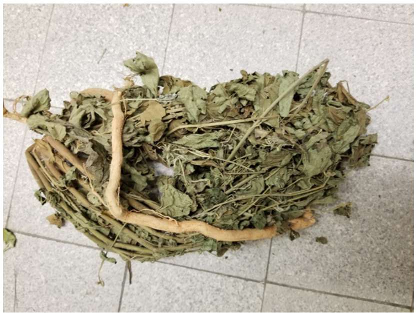

37. *Urena lobata*

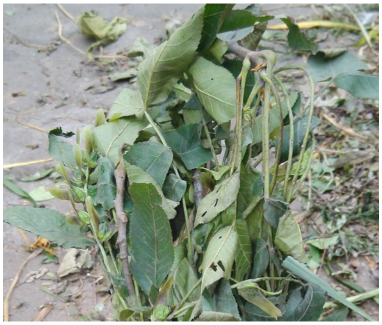

38. *Pterocarya stenoptera*

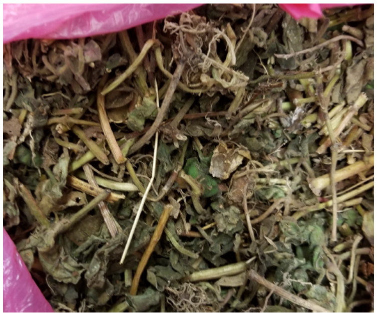

39. *Lysimachia alfredii*

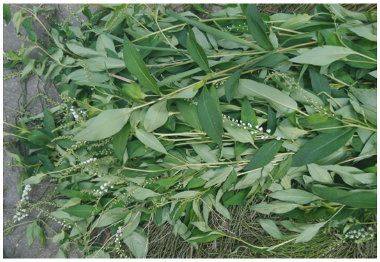

40. *Lysimachia fortunei*

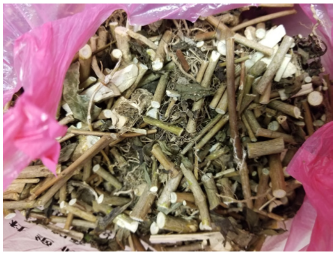

41. *Dichroa febrifuga*

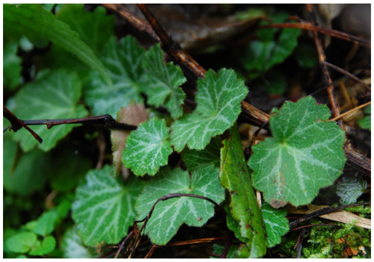

42. *Saxifraga stolonifera*

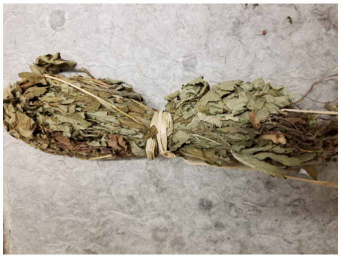

43. *Agrimonia pilosa*

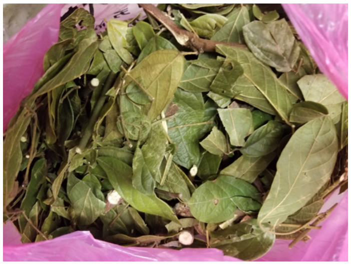

44. *Callerya dielsiana*

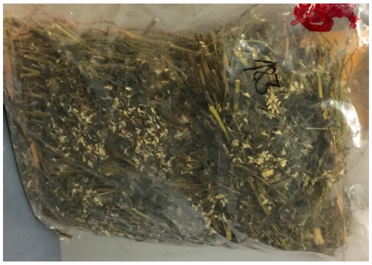

45. *Melilotus officinalis*

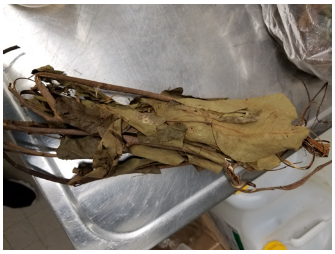

46. *Dalbergia hupeana*

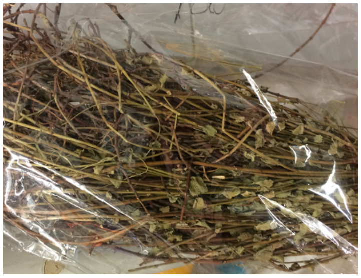

47. *Gonocarpus micranthus*

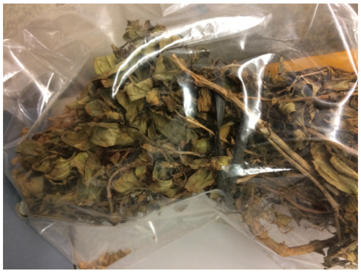

48. *Melastoma dodecandrum*

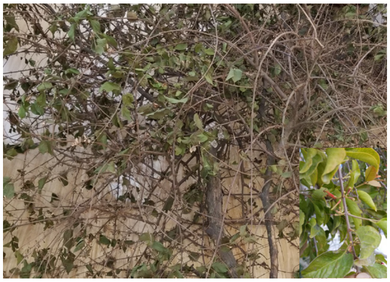

49. *Scurrula parasitica*

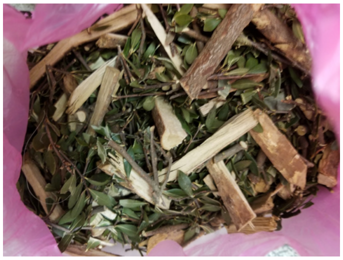

50. *Buxus sinica*

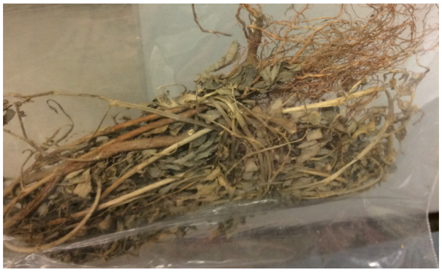

51. *Phyllanthus glaucus*

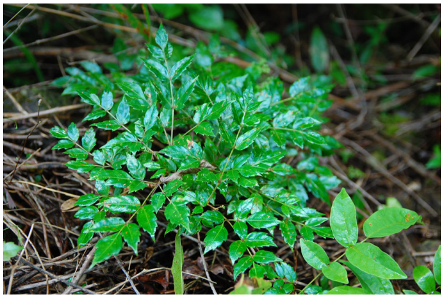

52. *Nekemias grossedentata*

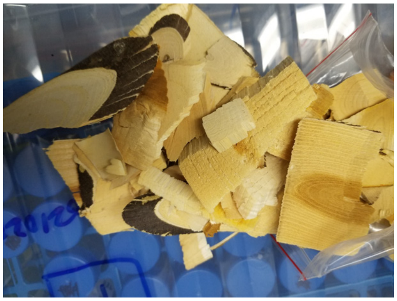

53. Stem of *Picrasma quassioides*

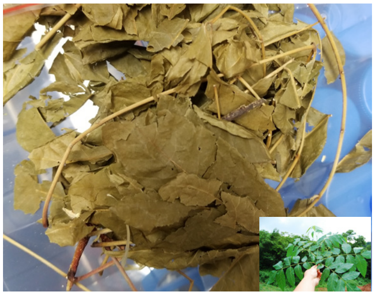

54. Leaf of *Picrasma quassioides*

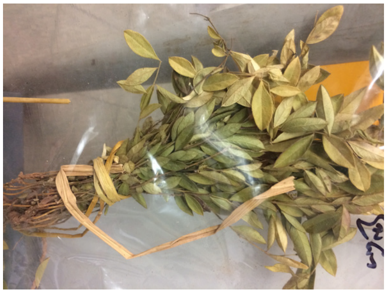

55. *Polygala japonica*

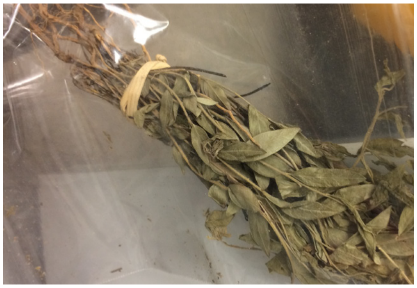

56. *Polygala angustifolia*

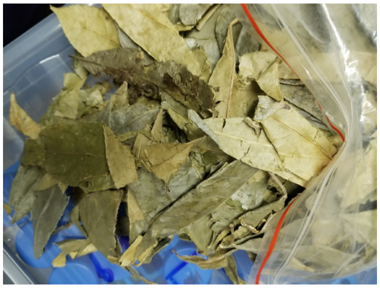

57. *Turpinia arguta*

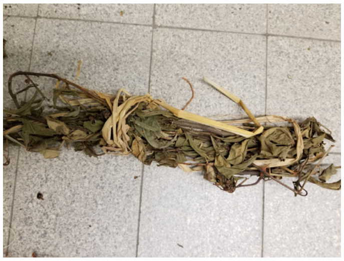

58. *Zanthoxylum simulans*

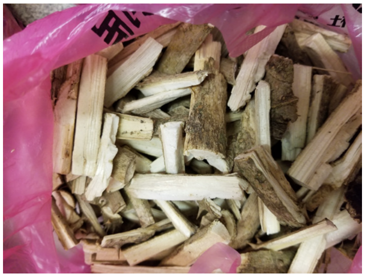

59. *Aralia elata*

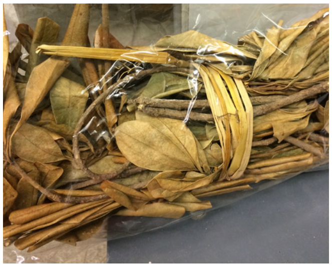

60. *Heptapleurum heptaphyllum*

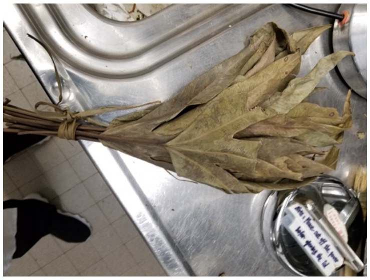

61. *Fatsia japonica*

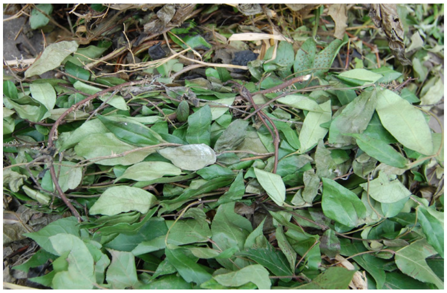

62. *Trachelospermum jasminoides*

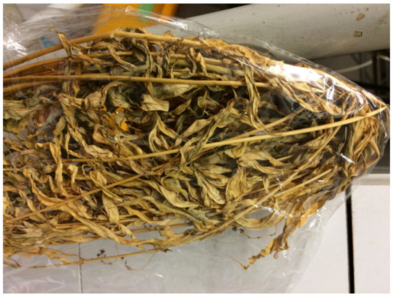

63. *Cynanchum stauntonii*

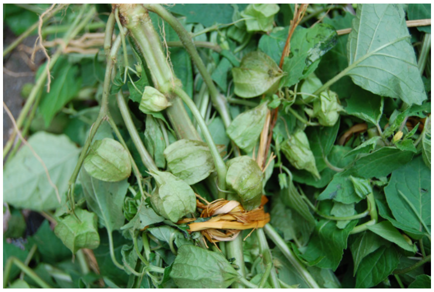

64. *Physalis angulata*

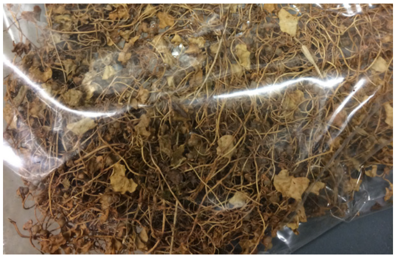

65. *Dichondra micrantha*

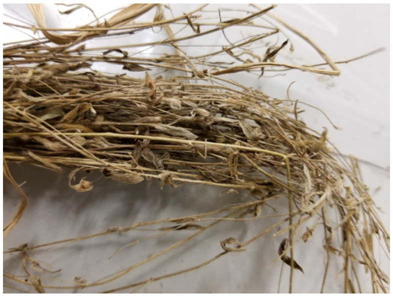

66. *Evolvulus alsinoides*

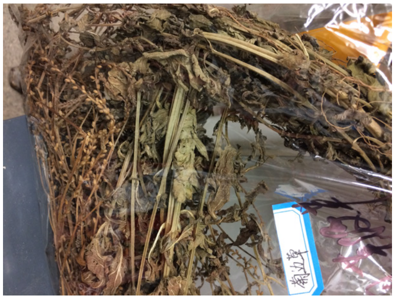

67. *Verbena officinalis*

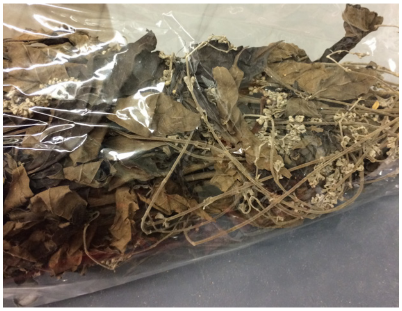

68. *Vitex negundo var. cannabifolia*

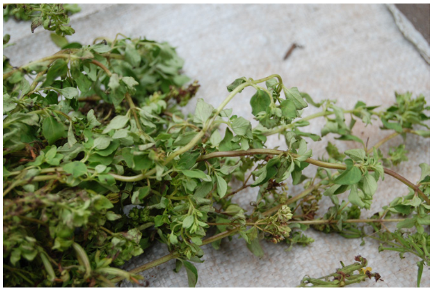

69. *Origanum vulgare*

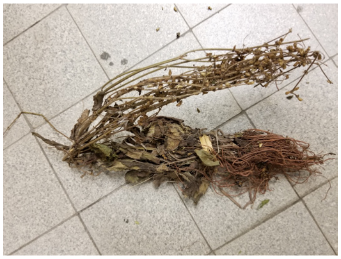

70. *Salvia prionitis*

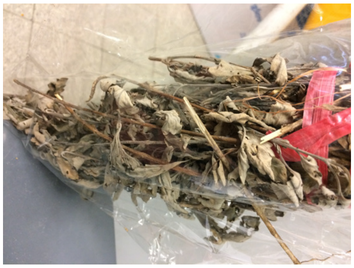

71. *Caryopteris incana*

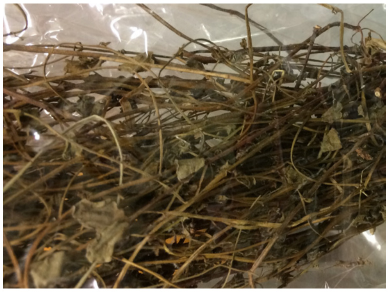

72. *Mosla scabra*

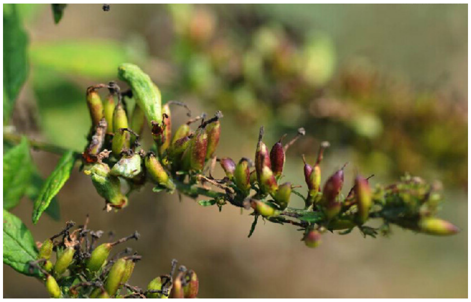

73. *Buddleja lindleyana*

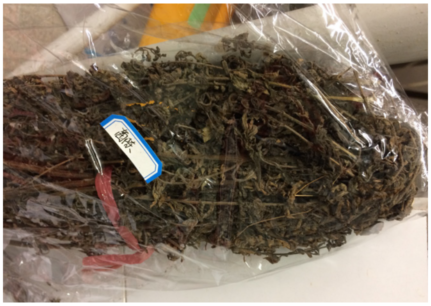

74. *Siphonostegia chinensis*
Local name:"(Tu) Yin Chen"

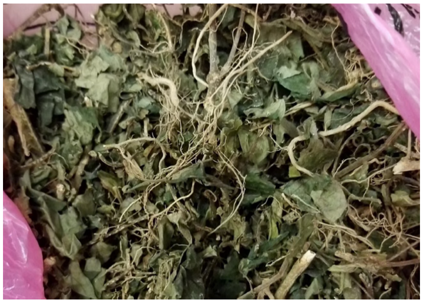

75. *Strobilanthes cusia*

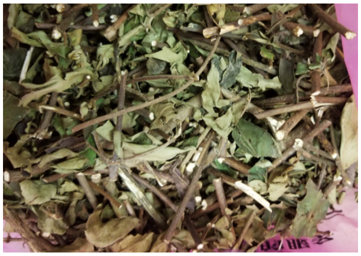

76. *Mussaenda pubescens*

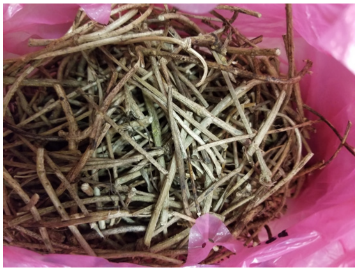

77. *Paederia foetida*

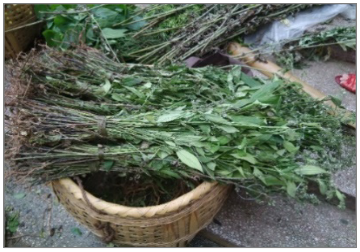

78. *Hedyotis mellii*

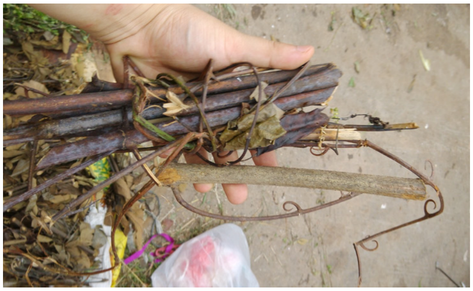

79. *Lycopodium anceps*

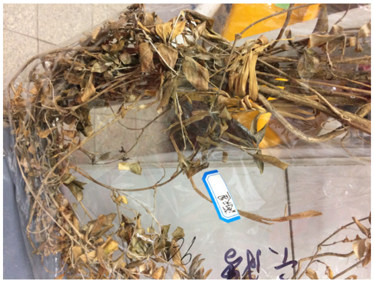

80. *Serissa japonica*

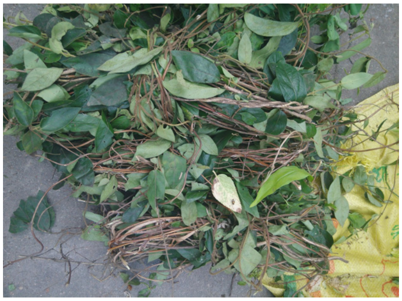

81. *Lonicera japonica*

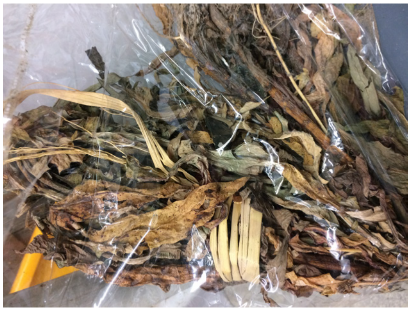

82. *Ixeris polycephala*

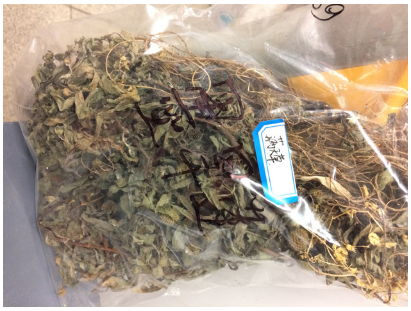

83. *Eclipta prostrata*

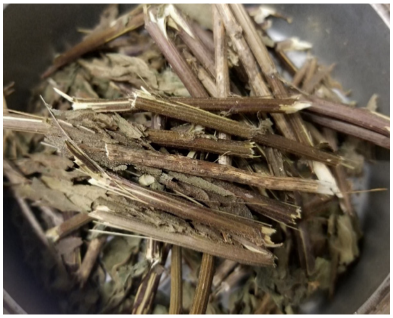

84. *Solidago decurrens*

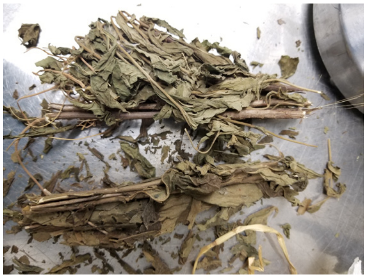

85. *Aster pekinensis*

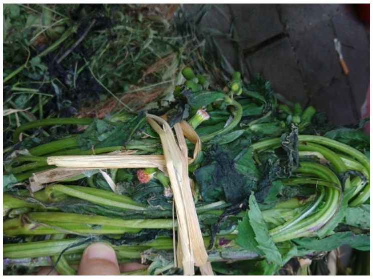

86. *Crassocephalum crepidioides*

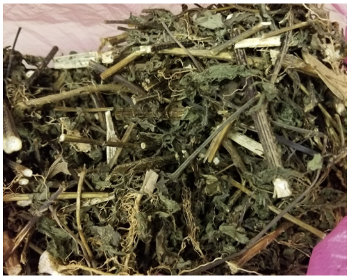

87. *Bidens pilosa*

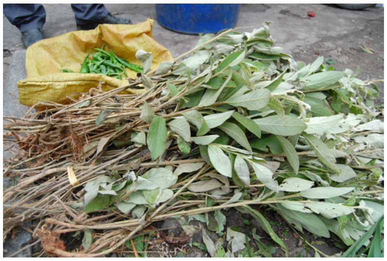

88. *Duhaldea cappa*

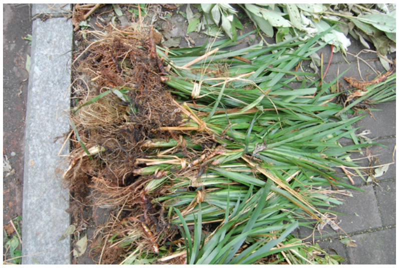

89. *Acorus gramineus*

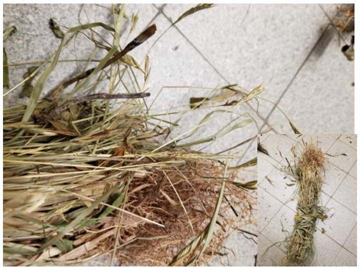

90. *Bromus japonicus*

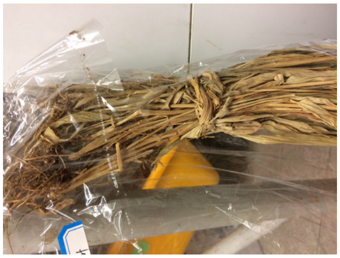

91. *Lophatherum gracile*

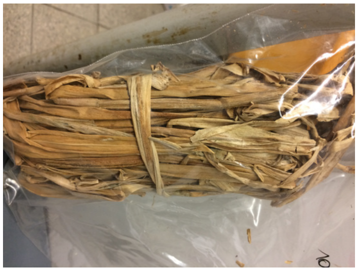

92. *Zingiber officinale*

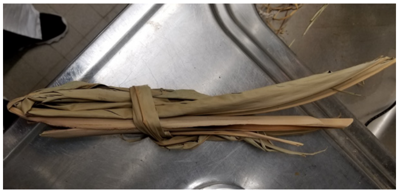

93. *Alpinia zerumbet*

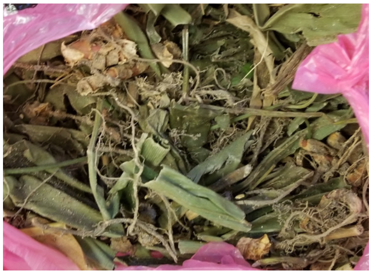

94. Herb of *Alpinia japonica*

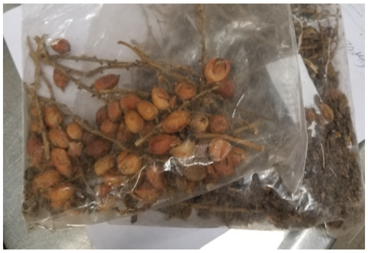

95. Fruit of *Alpinia japonica*

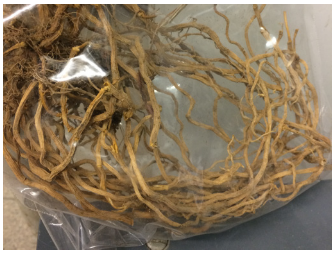

96. *Smilax riparia*

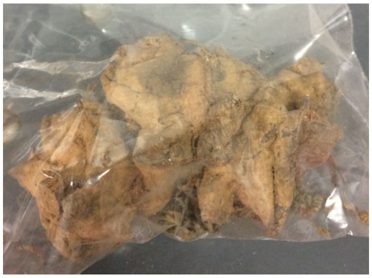

97. *Smilax glabra*
