# Supplementary material for: Ethnobotanical study of Hakka traditional medicine in Ganzhou, China and their antibacterial, antifungal, and cytotoxic assessments
Source: BMC Complement Med Ther. 2022 Sep 19;22:244. doi: 10.1186/s12906-022-03712-z (PMC9484230; doi:10.1186/s12906-022-03712-z)
Supplement: Supplementary file 3 — Additional file 3. [file 12906_2022_3712_MOESM3_ESM.pdf]

Supplementary Material 3 - Growth inhibition (%) of HTMs' extracts against human pathogens

**Plant name**	**Plant types#**	**Herb No.**	**Fungi**					**Gram-nagetive bacteria**									**Gram-positive bacteria**					
			**SC**	**CAU**	**CG**	**CA**	**CP**	**BD**	**SF**	**AB**	**PA**	**SLE**	**AH**	**EA**	**EC**	**SS**	**SE**	**BC**	**SA**	**EF**	**ML**	**LI**
Lycopodium japonicum	Perennial herbs	**1-1**	17	-12	24	50	7	15	0	-14	12	-24	0	-1	-23	-22	25	-10	4	13	55	50
		**1-2**	53	-17	7	54	25	59	23	5	-7	-42	-13	-51	37	1	19	-3	-3	4	99	105
		**1-3**	91	9	5	61	35	92	29	-10	1	-31	17	-40	34	21	23	-35	-49	-31	69	-40
		**1-4**	6	-16	6	-13	18	72	-2	-14	19	-19	32	15	-28	-11	2	-45	-48	16	26	52
Selaginella tamariscina	Perennial herbs	**2-1**	4	11	5	12	10	15	1	6	-4	-30	-41	20	9	-46	13	41	51	-1	30	11
		**2-2**	10	66	12	17	37	64	18	13	6	-29	-39	28	-9	42	1	80	88	9	80	60
		**2-3**	12	47	8	8	85	36	21	-26	-41	-48	-51	19	-8	-1	6	91	100	-38	73	-44
		**2-4**	18	-7	6	11	14	2	-10	-19	-34	-23	-46	9	-8	-2	21	-21	-4	7	19	3
Selaginella moellendorffii	Perennial herbs	**3-1**	11	-28	0	7	6	7	-7	-5	-4	-16	4	7	-24	-23	2	-13	14	-2	12	-11
		**3-2**	1	59	12	15	23	112	21	14	-4	-11	-18	26	19	-19	18	62	98	46	79	75
		**3-3**	14	28	8	7	66	26	11	-4	-24	-50	-48	12	-3	45	5	98	97	8	88	13
		**3-4**	25	12	30	31	48	-50	4	12	-27	12	-49	33	-16	30	43	42	24	34	37	20
Odontosoria chinensis	Perennial herbs	**4-1**	2	4	5	20	4	30	6	-7	2	21	-17	18	17	2	11	-48	21	-48	13	31
		**4-2**	4	3	7	29	15	51	8	-9	1	24	-19	17	28	19	21	-48	18	67	52	47
		**4-3**	2	-5	-1	-19	8	52	15	-13	-17	10	-8	19	28	3	6	-42	-10	-30	32	46
		**4-4**	6	4	8	1	6	91	4	-27	-25	16	-20	-24	1	-9	10	11	1	15	16	46
Adiantum flabellulatum	Perennial herbs	**5-1**	23	-2	4	10	9	-26	20	3	9	1	-5	17	21	-25	1	49	20	56	34	21
		**5-2**	12	61	12	18	88	30	22	19	30	-6	-22	40	12	57	23	96	84	67	84	52
		**5-3**	18	57	8	10	30	27	16	-5	-19	-31	-48	20	-15	19	4	96	-11	25	43	6
		**5-4**	40	17	5	23	35	10	53	-11	-18	-35	-41	14	19	-32	13	-48	69	-2	16	3
Selliguea hastata	Perennial herbs	**6-1**	22	-29	16	11	1	3	1	-2	-10	8	14	23	36	-23	2	-6	26	10	16	-4
		**6-2**	47	7	6	16	21	-4	19	-2	22	-33	3	33	14	20	15	105	94	-1	49	20
		**6-3**	23	5	7	8	48	-6	-5	17	-23	-44	-50	32	17	13	19	102	90	17	64	15
		**6-4**	34	0	25	29	37	-50	-3	7	-6	-14	-3	9	8	-18	17	-10	4	4	33	31
Loxogramme salicifolia	Perennial herbs	**7-1**	41	38	6	9	37	46	4	9	-1	67	-12	70	5	-7	8	-50	10	8	33	27
		**7-2**	70	-6	7	31	13	76	17	-2	8	27	16	26	21	65	5	-11	1	70	94	80
		**7-3**	57	60	5	14	6	61	45	8	26	58	9	33	72	3	30	21	-3	-42	67	11
		**7-4**	7	1	7	-2	4	77	-3	-6	-27	-10	2	-12	-38	-29	4	18	-12	15	16	23
Equisetum ramosissimum	Perennial herbs	**8-1**	30	37	5	37	5	40	16	14	15	53	-25	14	4	17	-7	-12	36	85	78	62
		**8-2**	17	8	11	38	18	59	-2	-1	-1	12	73	12	12	20	14	-11	103	54	115	115
		**8-3**	36	19	8	10	13	48	1	-7	-9	19	52	7	9	-18	9	40	94	-36	84	86
		**8-4**	4	-2	5	1	6	-22	62	21	35	83	99	38	27	46	52	-50	6	-6	16	-4
Fissistigma oldhamii **(root)**	Climbers	**9-1**	50	1	9	52	17	43	33	10	28	16	37	8	30	-25	39	24	22	1	81	83
		**9-2**	5	-2	12	58	28	108	21	18	32	23	47	9	25	61	40	34	49	89	115	97
		**9-3**	24	10	18	70	37	119	79	35	67	56	55	75	71	84	57	2	86	-48	94	-44
		**9-4**	30	35	6	62	25	97	13	29	35	-1	97	16	-10	23	14	4	28	-30	50	-5
Fissistigma oldhamii **(stem)**	Climbers	**10-1**	7	0	5	22	9	51	-1	-12	10	27	-7	15	0	7	10	26	32	17	14	59
		**10-2**	19	11	12	49	29	98	9	-7	25	26	4	15	30	60	20	16	74	10	83	43
		**10-3**	16	10	14	45	44	76	42	47	61	83	48	45	65	42	32	8	76	-7	84	88
		**10-4**	29	23	6	10	41	50	1	0	6	-4	45	-19	0	-1	15	-18	77	15	-7	43
Fissistigma oldhamii **(leaf)**	Climbers	**11-1**	64	-7	6	37	7	30	5	4	31	-10	19	6	25	-49	24	29	5	36	87	110
		**11-2**	21	-17	11	59	20	95	48	16	47	5	57	10	72	-37	14	30	75	42	109	84
		**11-3**	26	30	5	40	51	158	-13	-47	33	5	66	12	33	-6	30	61	51	11	48	96
		**11-4**	21	25	3	16	28	-15	50	23	31	41	74	6	81	36	25	-21	49	-2	113	17
Chimonanthus grammatus	Shrubs or trees	**12-1**	14	2	7	49	18	74	36	-2	24	-12	24	3	5	-4	18	10	35	12	75	0
		**12-2**	10	4	10	31	19	23	27	32	8	3	28	10	3	21	29	-6	8	41	71	33
		**12-3**	11	-12	0	43	14	6	75	39	30	73	26	38	52	29	71	-16	92	-48	50	37
		**12-4**	14	22	4	-29	-3	82	16	29	5	2	1	-22	10	-27	7	-21	39	34	-5	11
Lindera glauca	Shrubs or trees	**13-1**	12	13	9	9	12	35	20	14	43	38	5	15	21	1	27	-37	109	55	85	34
		**13-2**	10	23	17	39	34	123	-6	6	14	7	21	0	3	41	6	-45	95	3	124	140
		**13-3**	14	-3	12	52	30	70	50	-11	37	49	4	28	77	4	43	-40	75	-14	80	-50
		**13-4**	30	11	3	-50	10	57	63	37	32	32	11	7	68	79	54	-51	101	27	40	55
Cinnamomum jensenianum	Small trees	**14-1**	8	38	8	11	12	47	9	3	0	-29	-41	16	9	-40	10	36	13	-5	49	-1
		**14-2**	92	76	11	21	94	103	21	20	22	-22	-29	36	14	64	18	105	96	85	96	6
		**14-3**	48	58	10	15	82	66	-10	23	-4	-32	-47	26	17	38	17	96	89	20	70	-25
		**14-4**	8	-25	9	7	9	19	-7	2	-7	-24	-28	15	-8	-7	21	10	21	2	10	20
Saururus chinensis	Perennial herbs	**15-1**	76	4	12	24	8	48	-3	13	4	14	-29	23	27	-37	27	-21	-21	4	44	3
		**15-2**	9	-4	10	54	18	42	10	7	12	4	19	13	20	-28	12	17	24	31	60	-23
		**15-3**	-4	-7	7	18	12	68	1	-2	9	33	11	15	13	-20	30	21	10	28	32	-25
		**15-4**	33	10	5	16	8	90	5	-3	-6	20	48	5	35	-20	25	-20	32	-20	27	-50
Piper wallichii	Lianas woody	**16-1**	30	-3	6	49	10	53	65	14	47	39	1	41	67	9	43	34	41	43	97	36
		**16-2**	35	4	10	52	23	32	20	3	8	-5	18	2	11	23	18	-35	-30	41	88	28
		**16-3**	9	-4	2	-17	7	-50	-3	-13	-6	2	25	3	4	3	19	1	-35	-42	49	-14
		**16-4**	0	3	7	-48	3	42	-2	-19	2	-30	45	-17	-11	-21	11	-23	-41	27	-5	16
Asarum caudigerum	Perennial herbs	**17-1**	16	-2	6	9	8	-24	22	5	8	17	-23	36	18	-18	14	102	98	99	24	29
		**17-2**	15	42	14	21	78	118	24	21	4	-7	-31	37	21	16	-1	17	96	124	110	72
		**17-3**	4	31	3	8	81	43	17	60	-27	-12	-31	26	15	49	4	82	105	103	74	52
		**17-4**	1	-6	4	8	13	-2	38	-13	-39	-2	-42	18	-13	-1	2	-18	7	10	3	10
Sabia japonica	Lianas woody	**18-1**	26	49	3	22	58	-5	8	-32	-4	26	1	38	-11	14	15	27	62	19	82	42
		**18-2**	6	5	9	50	18	27	13	-10	-16	7	22	7	20	32	22	-10	35	4	88	80
		**18-3**	104	103	100	-7	0	53	-11	-24	-19	8	4	3	-6	-36	4	-15	23	-16	42	30
		**18-4**	100	99	99	-24	8	42	-8	-17	14	4	49	-11	-18	-49	6	42	-22	11	63	10
Liquidambar formosana	Trees	**19-1**	8	-3	5	33	6	10	10	-4	11	14	6	6	-18	1	22	44	-15	10	41	-49
		**19-2**	7	1	22	49	20	-50	14	-3	7	10	23	3	-42	23	23	28	80	88	74	52
		**19-3**	50	53	52	45	38	113	65	45	35	14	92	-6	34	-3	46	67	44	57	68	56
		**19-4**	28	41	-14	29	41	71	24	40	29	7	49	-16	-20	11	11	9	9	-42	19	59
Semiliquidambar cathayensis **(tender stem and leaf)**	Trees	**20-1**	9	-9	8	6	51	11	-18	-19	2	37	25	12	17	12	15	-2	0	21	26	33
		**20-2**	11	48	17	15	70	74	32	14	-9	27	13	17	18	81	39	139	113	-20	36	27
		**20-3**	33	74	20	26	83	86	26	77	10	6	-43	33	5	24	18	84	30	21	36	53
		**20-4**	20	-12	12	5	16	27	37	-5	-12	-17	19	11	15	1	26	-43	45	36	-45	50
Semiliquidambar cathayensis **(root)**	Trees	**21-1**	9	2	6	8	6	46	3	-7	-3	-14	-19	11	-20	-9	11	27	9	4	39	5
		**21-2**	7	59	13	20	51	73	24	26	-15	-23	-3	29	17	-14	22	76	10	127	61	97
		**21-3**	16	61	54	16	79	-14	30	9	-12	-7	2	11	38	9	-13	107	99	99	78	37
		**21-4**	-7	4	6	3	8	19	47	-8	-18	-35	-45	20	7	0	17	-16	63	1	16	14
Daphniphyllum macropodum	Trees or shrubs	**22-1**	48	-17	28	28	7	-8	22	-6	9	-23	-2	2	22	-36	16	-22	-11	-6	49	10
		**22-2**	10	8	11	35	13	26	23	-26	8	-19	44	6	27	30	22	10	-1	-18	59	99
		**22-3**	8	-29	10	17	65	24	19	-17	12	9	20	4	-21	-13	14	-26	-21	-19	48	51
		**22-4**	5	-7	8	14	1	83	62	-31	-7	-6	17	-16	-34	39	9	12	-40	14	-2	34
Ficus pumila	Lianas woody	**23-1**	56	0	11	36	8	22	36	-8	38	22	-2	26	35	48	35	4	-10	-50	86	4
		**23-2**	50	-9	10	26	18	5	20	-23	14	-15	21	11	13	-2	26	14	-29	40	96	98
		**23-3**	26	10	1	-49	7	75	3	-51	4	-15	4	-6	15	-31	33	13	47	39	86	69
		**23-4**	32	42	7	18	8	61	31	-36	-8	0	98	-22	-2	1	5	14	-44	-12	8	10
Ficus formosana f. *shimadai*	Shrubs	**24-1**	31	-2	8	20	4	-1	55	8	34	16	7	10	24	-42	30	16	24	15	64	34
		**24-2**	4	9	10	44	24	31	38	20	17	25	23	15	7	-28	36	38	-14	80	66	0
		**24-3**	18	7	3	-5	29	72	40	-6	19	51	17	39	35	-25	48	-26	81	94	57	-12
		**24-4**	50	11	9	3	15	54	6	-14	-9	6	25	-21	13	31	-10	19	25	123	17	44
Maclura cochinchinensis	Shrubs	**25-1**	9	0	8	13	9	18	43	22	4	-28	20	30	17	9	26	98	75	101	106	102
		**25-2**	3	43	86	19	4	80	62	71	45	14	40	62	54	32	51	112	99	137	147	119
		**25-3**	53	13	23	29	60	109	30	-3	-7	-4	15	4	-28	-15	-20	-16	100	12	96	16
		**25-4**	38	26	16	20	34	-20	38	-4	-34	-32	-47	19	6	10	21	10	70	3	18	-11
Ficus simplicissima	Shrubs	**26-1**	10	2	8	11	11	49	-12	-1	7	-20	-9	16	-1	-23	12	56	40	8	31	46
		**26-2**	21	22	16	9	83	51	19	12	7	6	1	33	13	31	30	98	91	-49	82	23
		**26-3**	8	3	9	5	80	30	4	-13	-45	-5	-23	28	15	30	17	87	43	-49	32	41
		**26-4**	21	8	10	6	16	194	-15	-2	-18	13	-25	-1	-2	23	1	-16	-9	140	119	167
Boehmeria nivea	Subshrubs or shrubs	**27-1**	1	0	34	25	13	33	9	-13	-2	6	-8	-9	16	-21	13	11	-6	60	28	13
		**27-2**	4	15	7	24	17	84	11	3	2	1	-3	7	1	24	19	-37	-6	95	80	94
		**27-3**	1	6	1	12	13	29	5	-12	-15	-3	13	4	13	56	13	-12	-48	89	73	18
		**27-4**	4	16	1	-14	8	-3	14	-7	-7	2	29	-3	12	-22	11	76	-29	53	-1	-10
Elatostema	Perennial herbs	**28-1**	44	34	6	17	45	30	10	3	6	78	-25	16	3	14	12	5	6	8	38	60

involucratum		**28-2**	52	2	8	34	14	43	-6	-4	2	3	35	7	10	14	5	14	-18	4	90	100
		**28-3**	37	10	2	15	7	27	49	3	22	62	-4	36	54	18	26	-4	-43	-49	64	12
		**28-4**	13	2	5	-5	5	-1	-20	-6	12	-14	9	-14	-34	-34	1	-4	-23	17	30	35
Phytolacca americana	Perennial herbs	**29-1**	77	-6	14	32	13	25	14	12	22	-7	-16	11	21	3	20	2	-34	28	47	-13
		**29-2**	5	-18	10	46	86	-35	22	-16	9	3	9	0	9	66	18	-6	17	9	94	92
		**29-3**	17	-21	2	9	11	11	19	8	-13	20	17	8	30	26	14	-19	-17	-18	39	-46
		**29-4**	14	7	11	-15	5	-2	40	112	6	30	48	9	8	35	23	-37	-31	-4	-4	-46
Polygonum chinense	Perennial herbs	**30-1**	8	-6	-1	5	17	49	33	5	21	5	17	18	14	35	32	28	-8	-3	73	64
		**30-2**	7	5	9	6	61	-8	26	-14	19	8	3	3	57	-9	0	-29	-41	-5	91	82
		**30-3**	4	-1	7	-19	60	84	44	2	24	48	37	19	52	41	46	-38	-45	-42	50	47
		**30-4**	9	6	-18	-50	60	91	20	99	19	9	99	-13	-54	2	9	13	40	23	-21	-13
Persicaria chinensis var. *paradoxa*	Perennial herbs	**31-1**	-6	0	5	33	13	69	-13	-17	1	-3	38	-4	9	10	-9	67	-1	18	102	100
		**31-2**	2	-3	11	40	20	26	-14	-37	3	2	-6	-23	-10	-39	-49	27	-10	-16	86	54
		**31-3**	-1	-5	1	30	15	73	-6	11	-11	30	1	33	22	-19	56	13	13	-28	83	22
		**31-4**	15	5	7	33	13	36	4	60	36	21	19	-12	-11	-7	23	-13	50	12	-21	30
Camellia oleifera **(infected leaf)**	Shrubs or trees	**32-1**	62	11	8	59	23	89	16	-4	28	9	40	13	27	45	20	65	107	-14	104	102
		**32-2**	12	-4	11	31	18	100	28	-6	17	-15	48	6	36	49	21	71	102	81	92	91
		**32-3**	101	101	100	52	95	93	12	13	12	-1	39	2	19	-13	25	69	60	-34	77	26
		**32-4**	28	-2	6	-48	-12	16	-19	18	22	5	54	4	-5	-4	-26	-16	23	-16	16	38
Adinandra nitida	Shrubs or trees	**33-1**	4	-3	-2	20	6	36	-4	-5	12	-18	21	-3	-13	-23	19	56	-7	33	99	99
		**33-2**	9	6	1	19	21	65	28	-11	-18	-6	14	-15	31	-1	21	-5	-49	40	68	69
		**33-3**	21	31	-8	14	57	109	65	46	49	45	65	47	86	60	41	0	98	23	-21	26
		**33-4**	32	53	24	10	38	153	43	28	64	0	52	7	17	77	39	-14	18	12	67	73
Eurya acuminatissima	Shrubs or trees	**34-1**	52	-5	2	-8	4	8	14	-4	7	-19	-18	-1	-3	13	23	0	2	74	82	24
		**34-2**	41	33	18	76	65	-51	82	26	23	26	33	19	59	50	62	-18	-4	58	47	69
		**34-3**	8	28	4	38	42	-50	21	-22	8	-9	47	8	-5	26	-16	29	-45	39	16	71
		**34-4**	35	9	9	68	42	29	60	-19	16	9	59	4	11	61	1	-21	-22	148	91	67
Hypericum japonicum	Annual herbs	**35-1**	17	-2	11	12	25	64	7	6	0	-14	-7	5	9	-31	7	76	98	-18	35	3
		**35-2**	1	93	19	17	90	113	11	18	3	9	100	22	16	30	18	50	101	110	109	104
		**35-3**	23	80	3	6	74	99	8	3	8	1	-5	13	19	51	17	94	99	14	52	66
		**35-4**	23	53	19	9	64	50	9	-8	11	24	-11	6	-2	2	10	-2	30	36	12	27
Corchoropsis crenata	Annual herbs	**36-1**	7	18	45	65	56	121	12	28	32	17	36	35	41	20	46	-24	79	26	114	62
		**36-2**	19	26	15	80	67	106	31	24	15	10	92	47	54	2	52	10	82	84	38	-49
		**36-3**	26	47	-19	68	35	149	76	8	3	78	75	33	68	56	38	-27	24	-47	123	29
		**36-4**	14	21	9	14	24	22	36	19	32	54	65	36	27	42	39	43	67	-1	41	18
Urena lobata	Subshrublike herbs	**37-1**	10	-40	7	6	75	78	-13	9	10	7	17	7	-6	-4	8	6	-7	-12	50	72
		**37-2**	13	10	11	13	84	7	13	18	21	5	-5	2	18	13	15	33	-27	-8	-6	40
		**37-3**	-1	26	17	17	73	39	6	16	6	-9	-6	20	19	17	10	97	-36	11	-42	13
		**37-4**	41	55	45	48	139	49	50	-1	6	-10	-9	11	27	10	2	17	5	62	70	92
Pterocarya stenoptera	Trees	**38-1**	9	-25	6	9	5	32	-19	-5	13	-41	-14	11	9	-11	-1	37	-4	-11	8	1
		**38-2**	20	24	15	13	73	26	13	8	-2	5	3	17	2	28	3	-3	-22	-15	39	2
		**38-3**	65	16	12	11	75	87	11	6	7	5	2	15	22	10	-2	58	70	58	87	20
		**38-4**	18	-5	11	10	17	8	-10	3	1	-20	-5	7	21	19	4	13	2	9	-10	28
Lysimachia alfredii	Perennial herbs	**39-1**	21	8	6	27	9	36	7	-19	70	30	23	89	70	2	32	20	-16	34	37	37
		**39-2**	19	28	18	-14	-3	66	-45	-32	16	-42	1	25	-3	-53	7	19	-37	101	171	77
		**39-3**	-5	-2	4	20	8	101	74	72	31	67	31	22	44	4	32	-45	-15	-47	-2	-47
		**39-4**	-8	3	7	-16	3	25	74	61	10	-9	14	14	20	12	35	-5	34	12	-48	-18
Lysimachia fortunei	Perennial herbs	**40-1**	6	2	5	38	15	-1	49	72	77	42	41	42	13	-1	55	58	114	6	73	13
		**40-2**	78	28	100	99	71	34	115	95	106	81	97	78	37	92	83	69	85	-1	81	72
		**40-3**	41	16	4	26	37	76	55	39	45	44	25	26	52	11	67	39	89	-28	110	-4
		**40-4**	23	71	18	-46	42	79	65	61	64	54	93	26	54	107	48	16	121	18	87	-45
Dichroa febrifuga	Shrubs	**41-1**	80	2	2	-16	71	3	13	-9	2	22	-5	26	-20	20	9	14	25	17	25	-3
		**41-2**	4	1	10	38	13	-9	-2	-1	-7	6	16	6	-14	17	12	20	39	-1	61	34
		**41-3**	3	0	4	-14	11	29	1	-5	-13	11	3	10	-45	7	6	71	34	-44	46	22
		**41-4**	5	2	3	17	5	-27	-2	-21	-10	-9	24	-13	-38	-18	-36	51	32	31	13	22
Saxifraga stolonifera	Perennial herbs	**42-1**	62	-12	6	29	11	18	12	12	1	22	-28	11	2	-1	7	-2	8	-12	80	37
		**42-2**	11	4	9	38	14	55	-2	2	-1	15	31	8	-1	-32	3	-7	1	-10	82	91
		**42-3**	6	-3	3	-9	7	73	13	10	9	33	73	37	39	-24	31	-49	53	-31	79	77
		**42-4**	19	58	8	-1	25	101	7	28	11	12	102	0	-7	-20	-21	-35	-36	42	23	44
Agrimonia pilosa	Perennial herbs	**43-1**	0	7	35	31	16	1	42	31	87	37	55	31	77	66	45	19	120	13	105	106
		**43-2**	22	28	23	-4	32	77	77	97	5	20	94	-36	38	69	62	-50	90	54	106	123
		**43-3**	25	-4	15	-46	27	42	-20	0	1	-1	13	4	32	-10	25	-49	82	-33	83	79
		**43-4**	28	65	11	-18	27	114	11	48	45	10	76	2	48	106	23	38	105	4	66	-45
Callerya dielsiana	Lianas woody	**44-1**	-3	2	6	8	8	107	43	9	20	-5	9	11	37	-49	-15	37	-18	51	110	133
		**44-2**	0	1	12	53	21	48	10	-16	1	12	12	-1	-17	-20	11	32	-38	121	71	35
		**44-3**	43	38	5	40	15	99	24	-16	6	3	22	-1	-2	-49	16	53	10	-27	68	81
		**44-4**	23	19	7	25	11	56	21	2	27	18	31	-7	31	-13	8	14	34	38	-10	30
Melilotus officinalis	Biennial herbs	**45-1**	1	12	5	9	7	22	6	12	-2	-17	-16	21	-13	-49	4	40	34	10	19	26
		**45-2**	42	31	12	18	86	79	21	47	4	-18	-33	41	20	25	12	91	100	78	70	19
		**45-3**	17	-2	5	8	12	23	7	4	-36	-37	-45	25	-6	-17	8	100	41	-31	21	18
		**45-4**	15	-11	7	7	14	42	-14	-5	-21	1	-24	9	-12	0	14	-10	-2	12	10	-5
Dalbergia hupeana	Trees	**46-1**	42	29	33	33	15	18	12	7	5	1	-8	13	25	12	15	-14	11	31	77	13
		**46-2**	17	-1	8	50	23	69	14	4	7	24	21	-7	28	-18	25	-23	17	95	49	75
		**46-3**	37	34	-1	-47	45	-49	100	5	57	39	62	-20	22	-8	56	5	49	22	77	-48
		**46-4**	10	9	4	4	6	99	36	-12	25	28	93	7	-1	50	15	-6	22	48	71	-50
Haloragis micrantha	Perennial herbs	**47-1**	9	3	5	12	14	-4	-4	11	5	-41	-43	20	-12	-30	16	82	100	97	23	23
		**47-2**	23	69	97	25	88	104	31	40	-14	5	-4	52	3	52	17	66	92	108	103	47
		**47-3**	38	49	28	26	67	39	43	23	36	-41	-14	41	8	-43	16	85	97	-46	76	11
		**47-4**	32	29	33	22	58	21	19	18	-16	-17	23	15	23	15	9	-44	75	-3	20	7
Melastoma dodecandrum	Perennial herbs	**48-1**	7	-12	7	6	15	-28	11	6	2	-15	-2	5	2	-17	-4	9	11	-3	3	36
		**48-2**	76	18	45	13	46	58	-23	-13	-49	-12	-1	13	-10	26	-6	70	-1	43	79	57
		**48-3**	49	84	98	16	79	73	32	24	36	16	35	18	33	53	10	100	22	22	58	37
		**48-4**	6	-17	6	2	21	47	19	-15	2	-7	-6	5	15	-9	10	-49	34	7	2	33
Scurrula parasitica	Shrubs	**49-1**	4	0	5	28	9	38	3	-7	-4	-6	20	8	-4	-24	1	31	10	-2	60	44
		**49-2**	8	1	12	13	29	-10	-8	-40	-13	-4	38	6	-45	67	12	59	28	25	105	123
		**49-3**	14	-1	9	28	48	-2	3	-1	-2	2	2	5	14	-12	12	76	67	17	3	23
		**49-4**	33	29	17	29	40	45	24	28	25	20	14	-48	-2	-1	2	26	71	77	56	-51
Buxus sinica	Shrubs or small trees	**50-1**	-7	4	17	19	6	32	7	-18	-2	19	-32	4	14	19	0	30	25	4	30	46
		**50-2**	19	2	11	60	24	-20	7	17	-14	14	22	0	48	32	56	7	-13	-17	58	44
		**50-3**	31	17	-8	-39	23	58	54	10	45	58	59	23	58	31	8	-6	67	29	83	28
		**50-4**	46	25	41	42	36	122	21	96	86	-9	125	96	15	39	42	-13	90	33	55	119
Phyllanthus glaucus	Shrubs	**51-1**	11	-14	4	6	76	26	-22	4	2	28	-17	4	6	-25	-2	95	2	-24	13	23
		**51-2**	8	20	6	10	80	31	12	10	15	2	2	28	14	21	9	97	100	-24	6	28
		**51-3**	12	27	15	9	85	88	30	97	4	20	23	16	26	57	36	94	106	93	81	38
		**51-4**	-2	20	8	1	9	35	47	3	-2	-15	33	13	20	5	5	14	56	3	24	34
Nekemias grossedentata	Lianas woody	**52-1**	19	7	7	34	16	8	20	0	4	-13	6	12	7	-7	22	18	-22	13	4	54
		**52-2**	63	-3	10	30	21	4	21	-2	14	2	49	-12	-26	-30	27	-4	42	44	60	69
		**52-3**	33	19	64	11	28	24	15	-5	20	0	74	0	-37	37	30	-19	75	18	-3	-49
		**52-4**	26	37	11	33	22	104	21	-17	22	-7	52	-23	-2	-23	29	-49	66	40	67	36
Picrasma quassioides **(stem)**	Trees	**53-1**	3	-15	5	-3	10	21	27	-1	9	-27	-4	6	-6	0	15	24	-22	5	0	24
		**53-2**	8	2	9	45	16	70	36	-17	12	8	17	7	7	15	21	26	26	14	38	43
		**53-3**	21	32	11	9	28	32	58	5	42	43	12	24	-50	40	30	94	70	75	77	6
		**53-4**	24	15	10	55	25	93	25	-3	17	26	75	8	14	30	31	21	16	46	71	-9
Picrasma quassioides **(leaf)**	Trees	**54-1**	16	-1	7	23	12	34	25	0	7	-10	-7	5	21	-26	4	16	-6	-42	23	-46
		**54-2**	25	13	28	56	44	34	70	18	26	30	27	-9	42	42	74	-21	-22	116	71	59
		**54-3**	44	17	-4	4	29	80	17	-10	-11	0	39	-33	2	32	28	34	35	24	99	117
		**54-4**	10	5	8	17	13	-50	-5	98	6	34	100	-7	-1	39	13	3	-28	38	14	-50
Polygala japonica	Perennial herbs	**55-1**	0	-19	6	9	8	21	1	10	-5	-31	2	16	12	12	7	6	12	-2	25	1
		**55-2**	24	37	11	17	30	65	1	19	29	-42	-42	30	15	-37	25	90	100	-15	52	15
		**55-3**	32	10	-1	8	42	27	6	2	3	-26	-45	34	11	-17	12	96	77	2	48	12
		**55-4**	18	-14	6	10	15	30	5	-1	-10	-38	-43	17	18	-6	14	-17	-1	1	10	-2
Polygala angustifolia	Perennial herbs	**56-1**	7	-20	5	8	6	-8	-14	6	-4	-37	2	7	-24	-41	16	11	24	2	16	15
		**56-2**	1	44	13	17	33	14	15	18	10	-18	-33	24	3	5	1	90	99	100	58	9
		**56-3**	1	29	2	10	12	1	11	4	-14	-21	-47	22	-5	-43	15	101	99	-32	33	-10
		**56-4**	6	-12	4	9	13	-5	18	-16	-24	-36	-45	20	24	-1	6	1	9	4	11	18

Turpinia arguta	Shrubs	**57-1**	51	8	7	51	13	46	32	25	27	2	-7	18	13	-6	36	11	124	38	88	39
		**57-2**	25	31	15	58	26	63	-12	-47	-28	-13	9	-11	31	-31	10	-16	74	-19	122	124
		**57-3**	33	15	90	45	55	78	96	112	28	25	90	12	47	-10	44	-40	25	-45	84	53
		**57-4**	30	14	57	41	53	76	82	68	39	10	97	-26	-2	48	24	-44	40	-15	97	53
Zanthoxylum simulans	Shrubs or small trees.	**58-1**	23	-8	3	-17	9	15	0	9	-2	-1	-31	5	-36	-45	-26	-7	26	-30	54	37
		**58-2**	0	-10	7	21	19	65	5	-11	-5	5	-2	-2	-12	5	-13	-19	55	-46	65	79
		**58-3**	-2	-10	7	5	14	71	34	25	-4	36	-3	12	40	31	17	-9	-47	-47	53	46
		**58-4**	26	41	-4	23	39	105	15	-5	-6	16	44	-13	-13	-15	-14	-1	42	-22	76	-42
Aralia elata	Shrubs or small trees.	**59-1**	5	11	6	25	21	58	14	1	-2	36	-10	25	16	14	23	31	26	-8	33	34
		**59-2**	-7	13	10	9	17	61	20	-11	0	17	21	5	16	22	14	22	14	-44	54	55
		**59-3**	9	5	4	21	21	73	7	-5	-10	6	20	11	10	12	9	-7	2	-8	5	36
		**59-4**	12	0	8	-18	3	32	3	-11	-5	19	23	-3	-40	29	-11	-19	2	15	-16	35
Heptapleurum heptaphyllum	Trees	**60-1**	6	17	6	7	10	59	13	2	9	-1	3	13	8	10	23	61	50	-21	20	32
		**60-2**	9	27	14	19	43	96	11	7	14	-18	-9	22	14	38	-3	86	100	-37	86	27
		**60-3**	25	15	7	7	39	29	0	-17	4	-5	8	26	5	3	11	39	6	22	14	21
		**60-4**	21	36	11	7	17	44	43	14	5	11	17	8	25	41	18	21	10	-44	28	26
Fatsia japonica	Shrubs	**61-1**	34	-12	13	9	16	74	22	0	20	46	-23	26	10	57	41	61	25	108	79	-2
		**61-2**	18	-24	10	7	18	75	70	25	20	41	-3	28	10	-13	39	-27	12	58	70	-10
		**61-3**	26	-6	4	14	7	74	36	-9	3	36	-24	5	35	-7	12	-27	-26	81	47	-43
		**61-4**	6	5	8	20	5	77	14	98	-3	-2	54	-20	-7	-8	-10	-31	-39	-52	8	-49
Trachelospermum jasminoides	Lianas woody	**62-1**	10	-5	12	12	13	72	7	3	3	-11	-6	8	9	-23	8	19	22	-11	18	30
		**62-2**	37	28	12	14	86	67	9	7	12	1	-7	30	11	26	20	99	98	-19	51	9
		**62-3**	12	7	5	4	65	30	-3	-22	-10	-11	-14	22	17	13	12	3	-54	22	-7	23
		**62-4**	21	4	8	7	13	35	-5	-25	-16	16	-11	2	-22	5	19	-12	-15	10	1	27
Cynanchum stauntonii	Perennial herbs	**63-1**	11	-30	3	8	-4	28	-4	-2	10	-32	-6	12	0	-43	4	37	9	-5	23	15
		**63-2**	6	33	10	10	81	33	17	27	5	23	17	39	8	19	18	102	94	-46	70	28
		**63-3**	9	6	2	5	82	15	2	4	-9	7	-9	23	14	75	9	30	-15	-18	12	24
		**63-4**	8	11	7	6	20	27	35	-6	-19	-10	5	5	-7	-1	-3	-12	3	-11	-1	27
Physalis angulata	Annual herbs	**64-1**	4	-3	5	32	13	77	-4	12	8	-9	-7	16	8	-21	9	-3	52	4	61	52
		**64-2**	15	6	12	33	25	103	-1	19	8	5	33	17	7	80	18	-25	108	113	97	88
		**64-3**	102	102	6	9	41	93	-8	-9	-14	10	38	12	-1	14	8	-4	-5	42	67	9
		**64-4**	100	98	99	69	100	-46	53	55	48	60	99	29	62	8	32	50	16	-8	6	-44
Dichondra micrantha	Perennial herbs	**65-1**	2	-9	6	10	8	24	3	3	20	-38	-50	21	25	-49	-2	9	2	0	56	4
		**65-2**	65	-6	12	50	77	64	16	17	50	-22	-32	41	17	55	18	91	24	80	95	52
		**65-3**	44	-10	5	9	86	22	8	11	27	-23	-52	34	14	17	13	45	-11	-9	63	14
		**65-4**	17	0	10	9	13	45	22	1	-3	-33	0	12	-5	-6	26	-3	-1	6	14	18
Evolvulus alsinoides	Perennial herbs	**66-1**	40	5	4	28	5	-16	8	-4	1	34	-26	26	-21	6	-17	-15	-3	74	69	35
		**66-2**	9	-2	9	23	11	18	6	16	-10	4	13	16	-15	60	11	-24	3	37	82	58
		**66-3**	12	-9	10	15	16	83	18	13	-11	20	5	12	27	79	9	5	61	-9	90	63
		**66-4**	3	5	7	19	5	57	16	10	-15	17	79	1	1	21	-5	-4	-33	7	-8	-25
Verbena officinalis	Herbs, annual or weakly perennial	**67-1**	20	-14	10	11	10	59	13	-4	14	-6	-9	20	25	-15	19	96	26	-12	35	28
		**67-2**	19	33	15	15	89	84	0	-13	-46	-5	-13	15	9	81	22	97	89	43	78	46
		**67-3**	22	8	7	8	32	66	-3	-10	-19	-31	-20	22	15	-15	23	97	29	23	17	27
		**67-4**	17	-9	11	10	14	-1	12	-40	-49	28	-23	2	6	4	30	-7	-6	48	-4	39
Vitex negundo var. *cannabifolia*	Shrubs or small trees	**68-1**	-4	-3	8	10	9	101	17	-8	18	-1	3	14	19	-24	11	87	44	-3	77	72
		**68-2**	6	76	17	21	19	76	32	11	19	-2	-5	23	21	45	15	94	87	82	75	24
		**68-3**	13	15	5	5	19	57	3	6	9	-4	1	24	11	34	28	103	79	72	-3	17
		**68-4**	15	0	9	4	17	28	-10	3	-3	4	23	-3	-8	21	-1	-1	-7	-4	-13	26
Origanum vulgare	Perennial herbs	**69-1**	2	-1	4	32	9	-12	-21	8	7	-7	-25	43	-39	-36	17	76	-4	11	78	75
		**69-2**	15	-22	9	51	25	-47	37	1	-19	-6	44	13	-43	25	4	102	15	99	96	94
		**69-3**	-11	-14	5	21	12	-8	14	-19	-8	9	-1	5	12	-37	21	42	-14	-48	56	16
		**69-4**	17	9	8	-7	0	-8	-2	-17	-14	17	46	-5	13	-15	12	-1	-21	23	-19	39
Salvia prionitis	Perennial herbs	**70-1**	11	-11	8	26	12	100	22	-7	51	27	3	39	58	99	37	1	99	61	116	100
		**70-2**	14	49	43	79	52	94	14	-11	43	47	20	49	66	59	63	-27	7	69	120	99
		**70-3**	3	5	2	5	10	23	44	-14	21	48	-8	29	45	101	30	-49	17	-5	64	3
		**70-4**	40	23	5	36	8	-8	17	2	-25	20	97	-24	20	23	4	-45	36	-3	28	-47
Caryopteris incana	Perennial herbs	**71-1**	13	-13	5	9	79	68	1	3	7	-24	21	2	9	-20	13	27	16	-10	12	36
		**71-2**	0	44	13	13	78	108	19	19	6	17	73	24	20	54	25	85	102	36	64	38
		**71-3**	-4	6	-3	6	4	44	1	-9	2	5	0	27	18	0	20	34	-50	-10	-3	40
		**71-4**	10	16	12	7	11	55	-15	-5	-13	-15	1	6	2	13	13	-9	11	-6	8	46
Mosla scabra	Annual herbs	**72-1**	10	-17	6	8	43	16	41	6	8	-35	19	13	18	-20	4	7	4	3	8	29
		**72-2**	30	34	11	15	67	86	39	25	7	23	21	27	30	82	22	98	114	100	89	73
		**72-3**	25	20	34	24	83	88	47	55	18	25	29	7	36	71	35	99	108	23	54	72
		**72-4**	-12	-23	10	5	8	100	-13	-18	-20	-23	-25	8	-13	5	12	-35	27	92	77	98
Buddleja lindleyana	Shrubs	**73-1**	30	-24	3	18	8	-9	7	5	6	-14	-11	9	-38	21	30	63	-8	17	92	51
		**73-2**	68	-4	9	65	23	65	22	-25	2	-3	44	4	0	49	17	-1	55	-4	95	96
		**73-3**	56	75	90	4	13	53	0	-4	28	-10	34	5	-10	-25	27	-44	-47	-5	66	1
		**73-4**	48	27	0	-15	-7	62	16	18	1	-28	46	-7	15	-10	26	-12	-47	-3	30	-47
Siphonostegia chinensis	Annual herbs	**74-1**	84	0	7	26	77	19	-10	-15	9	-4	-17	23	6	47	14	11	79	43	31	22
		**74-2**	70	25	17	16	75	96	12	23	-8	27	13	33	12	46	22	108	100	57	57	30
		**74-3**	71	8	4	7	74	76	83	-12	-14	76	99	5	0	37	18	46	-37	14	46	-13
		**74-4**	21	-4	14	6	19	24	30	-20	-12	6	-17	-1	2	9	19	-18	-8	14	0	34
Strobilanthes cusia	Perennial herbs	**75-1**	61	-10	8	48	11	90	12	4	9	-5	-1	-3	-14	-8	24	-7	6	100	34	32
		**75-2**	12	2	16	60	87	99	20	-18	-4	0	40	-1	20	52	16	-12	-49	95	75	42
		**75-3**	7	-3	8	31	14	89	36	-15	5	22	-7	14	63	-5	25	-1	-28	-43	53	31
		**75-4**	6	6	5	-7	6	68	14	-11	5	-16	19	-3	-7	-12	19	-45	-14	1	-23	28
Mussaenda pubescens	Lianas woody	**76-1**	5	2	8	14	10	31	-2	26	10	-35	4	-2	5	-29	20	22	-14	0	57	46
		**76-2**	11	-2	8	33	20	22	5	-15	12	-14	-12	2	18	58	19	-5	-35	8	59	7
		**76-3**	18	-20	6	-25	15	41	3	-26	-2	-3	4	-5	9	-13	15	-12	-1	-20	23	-3
		**76-4**	11	7	9	-52	-11	-11	-14	-8	14	-2	25	-25	14	-18	-7	36	45	-42	24	45
Paederia foetida	Lianas woody	**77-1**	10	-22	5	38	6	-28	16	8	4	-26	-3	7	-28	-20	-16	-8	-39	117	49	36
		**77-2**	40	9	9	30	18	15	19	-33	-11	-30	13	-5	-49	33	26	-34	-31	-1	75	39
		**77-3**	-3	-5	5	14	9	76	9	0	-5	-16	12	-2	0	-18	26	-22	-53	99	9	9
		**77-4**	35	3	5	24	1	70	36	99	-6	1	66	-20	-16	3	20	-30	-51	-11	5	-2
Hedyotis mellii	Perennial herbs	**78-1**	1	-1	5	5	9	21	-12	-35	4	7	-25	3	-3	3	3	-3	18	-6	69	60
		**78-2**	15	8	19	20	36	91	-34	-25	-3	-24	20	9	14	39	24	-19	2	100	100	64
		**78-3**	-8	0	3	-64	17	51	24	-44	-10	18	-18	7	1	-22	9	1	8	-30	70	28
		**78-4**	28	13	0	-73	8	49	18	-24	7	-5	17	9	18	-16	2	11	-19	84	9	34
Uncaria rhynchophylla	Lianas woody	**79-1**	17	-5	5	33	9	-6	-2	2	-6	-23	-23	10	-5	32	8	29	21	6	56	29
		**79-2**	16	6	17	-66	17	-32	-11	10	11	10	17	17	26	62	27	10	22	2	38	48
		**79-3**	52	34	59	-39	71	75	118	43	48	41	75	-23	45	62	23	-5	100	8	19	-49
		**79-4**	23	27	7	-1	40	-11	14	-7	18	-24	36	-4	-1	18	-37	21	100	0	17	-46
Serissa japonica	Small shrubs	**80-1**	13	-15	8	7	50	16	-9	-3	5	-13	25	6	15	10	14	27	0	1	2	23
		**80-2**	7	34	12	15	83	42	-49	35	3	-42	-5	22	20	10	11	88	78	47	58	81
		**80-3**	9	8	11	8	87	27	-4	-48	-9	-12	-11	21	12	-14	23	16	-25	18	6	24
		**80-4**	5	-4	12	6	7	101	4	-6	-24	18	17	19	15	21	56	44	32	101	105	97
Lonicera japonica	Lianas woody	**81-1**	32	-23	30	26	14	-43	-11	12	-10	-11	-27	-12	11	-14	3	-11	-5	7	71	32
		**81-2**	-3	6	6	45	18	51	31	3	28	36	19	16	33	69	5	-37	59	24	79	13
		**81-3**	-10	-13	3	10	13	66	5	-15	-2	16	21	9	14	-45	12	-50	-16	-29	54	-44
		**81-4**	22	4	5	7	11	42	47	-21	9	19	49	2	52	32	-2	-2	8	21	7	-51
Ixeris polycephala	Annual herbs	**82-1**	10	-33	6	7	6	41	-6	-12	9	-34	-9	5	-3	-8	4	93	8	-2	32	35
		**82-2**	4	30	10	14	82	60	18	12	0	17	7	28	14	21	16	86	97	-47	38	23
		**82-3**	2	4	3	5	74	19	0	4	-5	4	-3	21	16	-12	10	15	-24	-29	-2	29
		**82-4**	53	34	48	47	83	25	-7	0	-4	-11	20	3	5	-5	-2	5	0	1	-8	5
Eclipta prostrata	Annual herbs	**83-1**	14	-40	11	9	53	-11	2	9	12	-10	5	15	17	-30	12	32	100	-8	19	27
		**83-2**	2	31	15	16	73	66	-10	-3	3	-38	-25	23	22	59	6	75	101	56	90	47
		**83-3**	3	13	8	11	78	68	9	7	21	5	4	23	17	54	16	95	61	31	46	29
		**83-4**	10	-7	7	8	3	14	-11	0	6	-13	3	8	-1	11	13	10	8	1	-10	36
Solidago decurrens	Perennial herbs	**84-1**	18	-3	-1	33	9	4	-3	-7	-5	-12	0	1	13	-15	8	17	37	62	89	41
		**84-2**	1	-5	6	49	23	65	-3	9	-3	-5	22	5	20	-11	10	-45	102	88	80	66
		**84-3**	-15	2	2	7	12	-5	6	-21	-7	-5	3	4	33	18	10	-13	31	9	72	10
		**84-4**	-11	-21	12	0	12	82	-5	99	-7	-1	40	-7	9	29	20	-51	-22	-9	59	37
Aster pekinensis	Perennial herbs	**85-1**	3	-14	1	-7	10	-50	15	7	8	-12	5	9	-13	8	7	-20	7	54	100	85
		**85-2**	2	-2	8	21	14	45	-14	-19	3	-8	30	9	-27	28	14	-34	-9	-48	81	84
		**85-3**	5	-2	3	0	11	71	31	-3	-4	34	1	22	35	20	22	34	37	78	68	70

		**85-4**	11	-3	7	-26	5	-45	2	-12	3	-11	33	-2	4	-5	1	-38	-29	-29	-24	13
Crassocephalum crepidioides	Annual herbs	**86-1**	5	-19	11	41	16	66	12	8	17	28	2	4	34	-4	20	15	22	1	96	41
		**86-2**	10	0	10	10	40	89	17	-6	11	6	25	1	4	75	22	-32	105	11	112	109
		**86-3**	12	1	2	10	82	66	43	2	23	42	15	13	35	19	24	-31	19	1	97	100
		**86-4**	10	4	5	-48	7	54	68	33	37	65	57	36	47	65	36	-5	22	74	95	62
Bidens pilosa	Annual herbs	**87-1**	14	1	7	29	11	19	0	4	3	-4	-12	7	11	5	11	33	24	14	49	52
		**87-2**	-2	-4	4	6	14	15	13	3	-10	3	-3	22	21	11	10	-13	0	22	56	25
		**87-3**	5	-4	3	-16	13	-33	10	12	-7	16	20	8	25	-16	8	-22	12	-42	65	39
		**87-4**	8	5	7	-47	7	33	-11	-11	2	3	14	-4	9	-13	2	23	-8	-14	-6	30
Duhaldea cappa	Shrubs	**88-1**	7	-10	5	27	10	61	-6	5	15	-5	0	8	-28	-19	-5	-13	16	101	85	47
		**88-2**	0	0	11	55	24	61	6	7	2	-6	-5	8	-5	32	2	76	-55	34	73	41
		**88-3**	11	-1	4	39	14	-21	30	-19	-3	-8	6	6	11	-6	-3	65	-42	94	42	23
		**88-4**	15	5	6	-41	12	20	15	-8	0	-5	41	-19	-4	-11	-4	65	-24	-2	-18	48
Acorus gramineus	Perennial herbs	**89-1**	10	-7	7	11	14	40	-4	1	1	-53	13	11	0	-23	0	97	74	8	32	5
		**89-2**	34	66	17	19	95	100	19	7	24	-25	-11	27	12	32	5	82	91	84	81	11
		**89-3**	-8	27	10	10	82	10	13	19	-11	-22	-34	33	12	15	17	92	93	-5	33	12
		**89-4**	7	-5	10	9	13	-2	-6	1	-6	-37	3	14	-7	3	20	3	3	16	16	13
Bromus japonicus	Annual herbs	**90-1**	2	-4	1	12	6	12	13	7	2	-3	-4	13	-28	16	14	75	2	120	46	-34
		**90-2**	-4	-5	13	28	25	-36	1	7	-8	-22	-22	3	23	60	17	11	-16	98	58	8
		**90-3**	-4	6	4	0	15	-46	-6	-4	-13	-6	-24	-2	27	26	-47	-47	-38	12	23	-28
		**90-4**	29	11	5	-36	8	91	-3	-30	-32	-15	-49	2	4	2	14	11	98	-6	-36	29
Lophatherum gracile	Perennial herbs	**91-1**	16	-22	6	9	9	-41	-15	4	-1	-51	-35	15	8	-20	-13	0	11	12	10	2
		**91-2**	55	21	9	19	84	78	11	13	13	-24	-39	34	-1	40	1	93	91	35	7	7
		**91-3**	7	33	4	10	84	-46	17	-7	-46	-18	-16	38	13	44	20	100	48	31	74	53
		**91-4**	0	-5	7	8	12	-47	74	7	-25	9	-25	36	29	37	52	-49	22	1	43	60
Zingiber officinale	Perennial herbs	**92-1**	6	11	5	9	11	-48	-19	-1	9	-18	-3	9	-17	21	1	-21	3	-18	-9	6
		**92-2**	6	34	11	15	78	37	18	19	5	-3	-3	20	1	36	2	37	-1	-36	43	25
		**92-3**	5	14	7	8	77	12	7	-8	6	-5	3	18	13	20	5	-49	-14	-13	7	6
		**92-4**	16	-21	8	8	13	-5	-10	3	-3	13	75	5	-2	27	11	-8	-29	4	-6	5
Alpinia zerumbet	Perennial herbs	**93-1**	1	4	-2	45	22	100	51	17	48	18	38	32	51	32	37	58	48	99	100	97
		**93-2**	10	10	16	69	43	70	-2	-7	19	-18	101	8	18	13	-3	1	75	8	90	-48
		**93-3**	4	-3	8	24	29	55	51	12	29	50	-1	32	56	34	32	53	27	-19	61	-37
		**93-4**	24	33	-11	-42	37	78	25	23	6	21	62	-5	20	44	23	-10	70	11	61	56
Alpinia japonica **(herb)**	Perennial herbs	**94-1**	2	-14	12	44	7	37	49	9	34	12	39	26	20	-19	22	28	51	20	85	60
		**94-2**	3	-21	13	60	14	-50	9	-9	20	11	29	13	-46	-43	23	46	16	72	86	72
		**94-3**	-1	-14	10	18	48	-50	42	-2	16	19	13	15	-32	-6	25	2	12	32	43	21
		**94-4**	30	-32	7	-16	44	49	54	-22	-9	-36	14	-11	-5	-33	2	-45	-5	29	-37	30
Alpinia japonica **(friut)**	Perennial herbs	**95-1**	49	54	38	7	13	18	27	22	16	33	11	14	47	36	23	-43	18	57	72	-16
		**95-2**	67	-8	9	56	25	-3	15	1	10	12	0	11	77	-20	17	67	25	42	79	-51
		**95-3**	33	-4	5	-13	15	13	7	-26	-1	14	34	9	16	4	16	76	-15	44	69	-24
		**95-4**	24	17	10	9	10	-24	26	-18	-19	23	103	16	13	107	9	23	57	-48	34	-5
Smilax riparia	Vines annual or sometimes perennial	**96-1**	19	-25	10	11	11	15	0	4	-6	-23	7	9	6	-18	0	9	7	1	21	14
		**96-2**	22	9	11	15	79	-47	13	0	4	-37	-49	39	9	16	16	40	27	35	61	19
		**96-3**	40	-2	4	10	78	-19	1	-31	-48	-44	-50	30	8	30	11	38	21	46	50	20
		**96-4**	13	-19	8	9	8	-3	-4	-11	-27	-53	-21	3	17	0	-2	-23	7	14	10	3
Smilax glabra	Vines climbing, unarmed.	**97-1**	18	-41	7	7	75	18	-2	-1	2	27	21	14	2	-18	18	31	4	-11	13	28
		**97-2**	40	1	2	9	79	89	15	21	3	-2	5	30	15	69	32	101	75	-19	55	27
		**97-3**	61	27	12	31	77	100	0	12	12	14	15	-18	23	-21	12	52	38	58	94	91
		**97-4**	36	46	6	26	78	83	41	22	-5	10	-5	24	15	7	7	31	46	17	52	30
**Total number of active extracts (IV>50%)**			**39**	**30**	**15**	**35**	**87**	**168**	**37**	**22**	**12**	**20**	**49**	**7**	**30**	**50**	**18**	**95**	**117**	**83**	**206**	**110**

(1) -1 Hexane; -2 Ethyl acetate; -3 Methanol; -4 Water

(2) Plant types# is from the records in Flora of China.

(3) Fungal strain:*Saccharomyces cerevisiae* (SC), *Candida auris* (CAU), *Candida glabrata* (CG), *Candida albicans* (CA) and *Candida parapsilosis* (CP);

Gram-negative bacteria: *Brevundimonas diminuta* BD), *Shigella flexneri* (SF), *Acinetobacter baumannii* (AB), *Pseudomonas aeruginosa* (PA), *Salmonella enterica* subsp. *enterica* (SLE), *Aeromonas hydrophila* (AH), *Enterobacter aerogenes* (EA), *Escherichia coli* (EC) and *Shigella sonnei* (SS);

Gram-positive bacteria: *Staphylococcus epidermidis* (SE), *Bacillus cereus* (BC), *Staphylococcus aureus* (SA), *Enterococcus faecalis* (EF), *Micrococcus luteus* (ML) and *Listeria innocua* (LI).
