# Supplementary material for: Ethnobotanical study of Hakka traditional medicine in Ganzhou, China and their antibacterial, antifungal, and cytotoxic assessments
Source: BMC Complement Med Ther. 2022 Sep 19;22:244. doi: 10.1186/s12906-022-03712-z (PMC9484230; doi:10.1186/s12906-022-03712-z)
Supplement: Supplementary file 4 — Additional file 4. [file 12906_2022_3712_MOESM4_ESM.pdf]

Supplementary Material 4 - IC_{50} of HTMs' extracts against human pathogens

Plant name	Herb No.	Fungi					Gram-nagetive bacteria									Gram-positive bacteria					
		SC	CAU	CG	CA	CP	BD	SF	AB	PA	SLE	AH	EA	EC	SS	SE	BC	SA	EF	ML	LI
L. japonicum	1-1	–	–	–	992	–	–	–	–	–	–	–	–	–	–	–	–	–	–	992	–
	1-2	937	–	–	830	–	766	–	–	–	–	–	–	–	–	–	–	–	–	453	365
	1-3	345	–	–	875	–	302	–	–	–	–	–	–	–	–	–	–	–	–	818	–
	1-4	–	–	–	–	–	663	–	–	–	–	–	–	–	–	–	–	–	–	–	978
S. tamariscina	2-1	–	–	–	–	–	–	–	–	–	–	–	–	–	–	–	–	805	–	–	–
	2-2	–	853	–	–	–	843	–	–	–	–	–	–	–	–	–	939	**88**	–	413	476
	2-3	–	–	–	–	735	–	–	–	–	–	–	–	–	–	–	706	364	–	461	–
S. moellendorffii	3-2	–	797	–	–	–	597	–	–	–	–	–	–	–	–	–	992	374	–	**30**	**73**
	3-3	–	–	–	–	875	–	–	–	–	–	–	–	–	–	–	640	351	–	241	–
O. chinensis	4-2	–	–	–	–	–	983	–	–	–	–	–	–	–	–	–	–	–	986	833	–
	4-3	–	–	–	–	–	793	–	–	–	–	–	–	–	–	–	–	–	–	–	–
	4-4	–	–	–	–	–	476	–	–	–	–	–	–	–	–	–	–	–	–	–	–
A. flabellulatum	5-1	–	–	–	–	–	–	–	–	–	–	–	–	–	–	–	–	–	954	–	–
	5-2	–	840	–	–	873	–	–	–	–	–	–	–	–	983	–	671	530	808	221	932
	5-3	–	926	–	–	–	–	–	–	–	–	–	–	–	–	–	647	–	–	–	–
	5-4	–	–	–	–	–	–	826	–	–	–	–	–	–	–	–	–	**38**	–	–	–
S. hastata	6-2	–	–	–	–	–	–	–	–	–	–	–	–	–	–	–	733	**135**	–	–	–
	6-3	–	–	–	–	–	–	–	–	–	–	–	–	–	–	–	844	295	–	499	–
L. salicifolia	7-1	–	–	–	–	–	–	–	–	–	679	–	400	–	–	–	–	–	–	–	–
	7-2	949	–	–	–	–	643	–	–	–	–	–	–	–	642	–	–	–	769	510	974
	7-3	843	873	–	–	–	453	–	–	–	953	–	–	874	–	–	–	–	–	955	–
	7-4	–	–	–	–	–	239	–	–	–	–	–	–	–	–	–	–	–	–	–	–
E. ramosissimum	8-1	–	–	–	–	–	–	–	–	–	543	–	–	–	–	–	–	–	540	983	653
	8-2	–	–	–	–	–	978	–	–	–	–	929	–	–	–	–	–	244	974	**106**	431
	8-3	–	–	–	–	–	–	–	–	–	–	853	–	–	–	–	–	**182**	–	345	395
	8-4	–	–	–	–	–	–	305	–	–	493	842	–	–	–	974	–	–	–	–	–
F. oldhamii's root	9-1	953	–	–	956	–	–	–	–	–	–	–	–	–	–	–	–	–	–	923	529
	9-2	–	–	–	992	–	378	–	–	–	–	–	–	–	752	–	–	–	239	230	390
	9-3	–	–	–	864	–	**143**	309	–	492	763	826	398	530	299	975	–	982	–	**138**	–
	9-4	–	–	–	975	–	577	–	–	–	–	655	–	–	–	–	–	–	–	916	–
F. oldhamii's stem	10-1	–	–	–	–	–	925	–	–	–	–	–	–	–	–	–	–	–	–	–	758
	10-2	–	–	–	–	–	298	–	–	–	–	–	–	–	756	–	–	987	–	453	–
	10-3	–	–	–	–	–	375	–	–	372	739	–	–	433	–	–	–	349	–	232	874
	10-4	–	–	–	–	–	–	–	–	–	–	–	–	–	–	–	–	675	–	–	–
F. oldhamii's leaf	11-1	852	–	–	–	–	–	–	–	–	–	–	–	–	–	–	–	–	–	593	395
	11-2	–	–	–	952	–	492	–	–	–	–	583	–	582	–	–	–	306	–	295	473
	11-3	–	–	–	–	846	286	–	–	–	–	865	–	–	–	–	869	398	–	–	860
	11-4	–	–	–	–	–	–	925	–	–	–	483	–	295	–	–	–	–	–	925	–
C. grammatus	12-1	–	–	–	–	–	993	–	–	–	–	–	–	–	–	–	–	–	–	957	–
	12-2	–	–	–	–	–	–	–	–	–	–	–	–	–	–	–	–	–	–	494	–
	12-3	–	–	–	–	–	–	907	–	–	964	–	–	863	–	926	–	498	–	–	–
	12-4	–	–	–	–	–	935	–	–	–	–	–	–	–	–	–	–	–	–	–	–
L. glauca	13-1	–	–	–	–	–	–	–	–	–	–	–	–	–	–	–	–	382	926	843	–
	13-2	–	–	–	–	–	296	–	–	–	–	–	–	–	–	–	–	206	–	383	386
	13-3	–	–	–	894	–	264	–	–	–	–	–	–	383	–	–	–	**144**	–	984	–
	13-4	–	–	–	–	–	640	840	–	–	–	–	–	760	393	864	–	284	–	–	995
C. jensenianum	14-2	308	865	–	–	797	995	–	–	–	–	–	–	–	950	–	892	**150**	242	284	–
	14-3	–	975	–	–	860	947	–	–	–	–	–	–	–	–	–	904	362	–	265	–
S. chinensis	15-1	364	–	–	–	–	–	–	–	–	–	–	–	–	–	–	–	–	–	–	–
	15-2	–	–	–	926	–	–	–	–	–	–	–	–	–	–	–	–	–	–	938	–
	15-3	–	–	–	–	–	973	–	–	–	–	–	–	–	–	–	–	–	–	–	–
	15-4	–	–	–	–	–	760	–	–	–	–	–	–	–	–	–	–	–	–	–	–
P.wallichii	16-1	–	–	–	–	–	909	994	–	–	–	–	–	895	–	–	–	–	–	552	–
	16-2	–	–	–	928	–	–	–	–	–	–	–	–	–	–	–	–	–	–	973	–
A. caudigerum	17-1	–	–	–	–	–	–	–	–	–	–	–	–	–	–	–	872	820	997	–	–
	17-2	–	–	–	–	975	997	–	–	–	–	–	–	–	–	–	–	747	239	209	456
	17-3	–	–	–	–	924	–	–	864	–	–	–	–	–	–	–	636	699	355	209	997
S. japonica	18-1	–	–	–	–	843	–	–	–	–	–	–	–	–	–	–	–	995	–	782	–
	18-2	–	–	–	–	–	–	–	–	–	–	–	–	–	–	–	–	–	–	529	859
	18-3	874	976	864	–	–	840	–	–	–	–	–	–	–	–	–	–	–	–	–	–
	18-4	834	884	986	–	–	–	–	–	–	–	–	–	–	–	–	–	–	–	375	–
L. formosana	19-2	–	–	–	–	–	–	–	–	–	–	–	–	–	–	–	–	278	**191**	310	847
	19-3	–	839	986	–	–	986	566	–	–	–	995	–	–	–	–	569	–	485	774	854
	19-4	–	–	–	–	–	928	–	–	–	–	–	–	–	–	–	–	–	–	–	987
S. cathayensis's tender stem and leaf	20-1	–	–	–	–	864	–	–	–	–	–	–	–	–	–	–	–	–	–	–	–
	20-2	–	–	–	–	929	319	–	–	–	–	–	–	–	983	–	855	974	–	–	–
	20-3	–	937	–	–	964	985	–	824	–	–	–	–	–	–	–	930	–	–	–	993
S. cathayensis's root	21-2	–	406	–	–	305	306	–	–	–	–	–	–	–	–	–	972	–	404	893	204
	21-3	–	504	603	–	927	–	–	–	–	–	–	–	–	–	–	403	403	**104**	**103**	–
	21-4	–	–	–	–	–	–	–	–	–	–	–	–	–	–	–	–	385	–	–	–
D. macropodum	22-2	–	–	–	–	–	–	–	–	–	–	–	–	–	–	–	–	–	–	928	403
	22-3	–	–	–	–	967	–	–	–	–	–	–	–	–	–	–	–	–	–	–	609
	22-4	–	–	–	–	–	205	505	–	–	–	–	–	–	–	–	–	–	–	–	–
F. pumila	23-1	507	–	–	–	–	–	–	–	–	–	–	–	–	–	–	–	–	–	604	–
	23-2	732	–	–	–	–	–	–	–	–	–	–	–	–	–	–	–	–	–	205	302
	23-3	–	–	–	–	–	306	–	–	–	–	–	–	–	–	–	–	–	–	506	944
	23-4	–	–	–	–	–	984	–	–	–	–	406	–	–	–	–	–	–	–	–	–
F. formosana f. shimadai	24-1	–	–	–	–	–	–	755	–	–	–	–	–	–	–	–	–	–	–	401	–
	24-2	–	–	–	–	–	–	–	–	–	–	–	–	–	–	–	–	–	303	740	–
	24-3	–	–	–	–	–	396	–	–	–	894	–	–	–	–	–	–	486	207	850	–
	24-4	–	–	–	–	–	973	–	–	–	–	–	–	–	–	–	–	–	976	–	–
M. cochinchinensis	25-1	–	–	–	–	–	–	–	–	–	–	–	–	–	–	–	435	**166**	**138**	975	927
	25-2	–	–	972	–	–	**144**	595	967	–	–	–	879	241	–	634	**67**	**45**	**11**	947	980
	25-3	924	–	–	–	947	976	–	–	–	–	–	–	–	–	–	–	**38**	–	964	–
	25-4	–	–	–	–	–	–	–	–	–	–	–	–	–	–	–	–	**12**	–	–	–
F. simplicissima	26-1	–	–	–	–	–	–	–	–	–	–	–	–	–	–	–	993	–	–	–	–
	26-2	–	–	–	–	847	980	–	–	–	–	–	–	–	–	–	671	534	–	980	–
	26-3	–	–	–	–	969	–	–	–	–	–	–	–	–	–	–	991	–	–	–	–
	26-4	–	–	–	–	–	982	–	–	–	–	–	–	–	–	–	–	–	**190**	974	946
B. nivea	27-1	–	–	–	–	–	–	–	–	–	–	–	–	–	–	–	–	–	977	–	–
	27-2	–	–	–	–	–	307	–	–	–	–	–	–	–	–	–	–	–	205	395	396
	27-3	–	–	–	–	–	–	–	–	–	–	–	–	–	305	–	–	–	935	206	–
	27-4	–	–	–	–	–	–	–	–	–	–	–	–	–	–	–	496	–	936	–	–
E. involucratum	28-1	–	–	–	–	–	–	–	–	–	205	–	–	–	–	–	–	–	–	–	921
	28-2	875	–	–	–	–	–	–	–	–	–	–	–	–	–	–	–	–	–	206	496
	28-3	–	–	–	–	–	–	–	–	–	924	–	–	935	–	–	–	–	–	929	–
P. americana	29-2	–	–	–	–	758	–	–	–	–	–	–	–	–	982	–	–	–	–	741	406
	29-4	–	–	–	–	–	–	–	849	–	–	–	–	–	–	–	–	–	–	–	–
P. chinense	30-1	–	–	–	–	–	–	–	–	–	–	–	–	–	–	–	–	–	–	495	596
	30-2	–	–	–	–	956	–	–	–	–	–	–	–	936	–	–	–	–	–	207	744
	30-3	–	–	–	–	659	650	–	–	–	–	–	–	848	–	–	–	–	–	–	–
	30-4	–	–	–	–	875	302	–	396	–	–	395	–	–	–	–	–	–	–	–	–
P. chinensis var. *paradoxa*	31-1	–	–	–	–	–	865	–	–	–	–	–	–	–	–	–	395	–	–	684	769
	31-2	–	–	–	–	–	–	–	–	–	–	–	–	–	–	–	–	–	–	**105**	835
	31-3	–	–	–	–	–	439	–	–	–	–	–	–	–	–	740	–	–	–	306	–
	31-4	–	–	–	–	–	–	–	605	–	–	–	–	–	–	–	–	947	–	–	–
infected leaf of *C.oleifera*	32-1	993	–	–	936	–	929	–	–	–	–	–	–	–	–	–	727	292	–	957	967
	32-2	–	–	–	–	–	803	–	–	–	–	–	–	–	–	–	256	**71**	569	299	471
	32-3	985	995	488	966	938	910	–	–	–	–	–	–	–	–	–	833	656	–	924	–
	32-4	–	–	–	–	–	–	–	–	–	–	947	–	–	–	–	–	–	–	–	–
A. nitida	33-1	–	–	–	–	–	–	–	–	–	–	–	–	–	–	–	933	–	–	396	406
	33-2	–	–	–	–	–	873	–	–	–	–	–	–	–	–	–	–	–	–	**106**	695
	33-3	–	–	–	–	876	201	306	–	–	–	205	–	307	874	–	–	**110**	–	–	–
	33-4	–	535	–	–	–	433	–	–	694	–	946	–	–	597	–	–	–	–	395	497
E. acuminatissima	34-1	396	–	–	–	–	–	–	–	–	–	–	–	–	–	–	–	–	853	286	–
	34-2	–	–	–	986	784	–	395	–	–	–	–	–	756	875	295	–	–	986	–	838
	34-3	–	–	–	–	–	–	–	–	–	–	–	–	–	–	–	–	–	–	–	675
	34-4	–	–	–	998	–	–	880	–	–	–	975	–	–	460	–	–	–	**148**	870	786
H. japonicum	35-1	–	–	–	–	–	985	–	–	–	–	–	–	–	–	–	825	634	–	–	–
	35-2	**121**	985	–	–	995	424	–	–	–	–	925	–	–	–	–	–	**92**	958	**191**	841
	35-3	–	435	–	–	963	983	–	–	–	–	–	–	–	999	–	669	881	–	497	974
	35-4	–	938	–	–	904	977	–	–	–	–	–	–	–	–	–	–	–	–	–	–
C. crenata	36-1	–	–	–	904	930	875	–	–	–	–	–	–	–	–	–	–	762	–	769	678
	36-2	–	–	–	760	896	688	–	–	–	–	395	–	953	–	985	–	396	535	–	–
	36-3	–	–	–	936	–	395	425	–	–	562	588	–	769	826	–	–	–	–	896	–
	36-4	–	–	–	–	–	–	–	–	–	739	973	–	–	–	–	–	988	–	–	–
U. lobata	37-1	–	–	–	–	946	924	–	–	–	–	–	–	–	–	–	–	–	–	955	994
	37-2	–	–	–	–	837	–	–	–	–	–	–	–	–	–	–	–	–	–	–	–
	37-3	–	–	–	–	998	–	–	–	–	–	–	–	–	–	–	942	–	–	–	–
	37-4	–	880	–	–	974	–	968	–	–	–	–	–	–	–	–	–	–	955	935	936
P. stenoptera	38-2	–	–	–	–	979	–	–	–	–	–	–	–	–	–	–	–	–	–	–	–
	38-3	964	–	–	–	936	947	–	–	–	–	–	–	–	–	–	977	846	736	244	–
L. alfredii	39-1	–	–	–	–	–	–	–	–	874	–	–	496	675	–	–	–	–	–	–	–
	39-2	–	–	–	–	–	355	–	–	–	–	–	–	–	–	–	–	–	240	**171**	396
	39-3	–	–	–	–	–	276	345	548	–	284	–	–	–	–	–	–	–	–	–	–
	39-4	–	–	–	–	–	–	942	994	–	–	–	–	–	–	–	–	–	–	–	–

L. fortunei	**40-1**	–	–	–	–	–	–	–	928	864	–	–	–	–	–	865	890	904	–	969	–
	**40-2**	597	–	925	850	735	–	692	784	486	872	673	576	–	375	585	349	754	–	348	465
	**40-3**	–	–	–	–	–	638	598	–	–	–	–	–	825	–	329	–	295	–	986	–
	**40-4**	–	990	–	–	–	747	813	894	672	899	956	–	784	814	–	–	433	–	264	–
D. febrifuga	**41-1**	519	–	–	–	984	–	–	–	–	–	–	–	–	–	–	–	–	–	–	–
	**41-2**	–	–	–	–	–	–	–	–	–	–	–	–	–	–	–	–	–	–	543	–
	**41-3**	–	–	–	–	–	–	–	–	–	–	–	–	–	–	–	284	–	–	–	–
	**41-4**	–	–	–	–	–	–	–	–	–	–	–	–	–	–	–	653	–	–	–	–
S. stolonifera	**42-1**	839	–	–	–	–	–	–	–	–	–	–	–	–	–	–	–	–	–	698	–
	**42-2**	–	–	–	–	–	539	–	–	–	–	–	–	–	–	–	–	–	–	452	386
	**42-3**	–	–	–	–	–	675	–	–	–	–	883	–	–	–	–	–	690	–	758	285
	**42-4**	–	874	–	–	–	322	–	–	–	–	599	–	–	–	–	–	–	–	–	–
A. pilosa	**43-1**	–	–	–	–	–	–	–	–	693	–	936	–	840	386	–	–	865	–	920	921
	**43-2**	–	–	–	–	–	264	375	376	–	–	496	–	–	274	840	–	385	563	592	893
	**43-3**	–	–	–	–	–	–	–	–	–	–	–	–	–	–	–	–	296	–	740	748
	**43-4**	–	873	–	–	–	433	–	–	–	–	673	–	–	590	–	–	946	–	736	–
C. dielsiana	**44-1**	–	–	–	–	–	673	–	–	–	–	–	–	–	–	–	–	–	561	948	758
	**44-2**	–	–	–	927	–	–	–	–	–	–	–	–	–	–	–	–	–	354	633	–
	**44-3**	–	–	–	–	–	275	–	–	–	–	–	–	–	–	–	874	–	–	385	419
	**44-4**	–	–	–	–	–	994	–	–	–	–	–	–	–	–	–	–	–	–	–	–
M. officinalis	**45-2**	–	–	–	–	943	965	–	–	–	–	–	–	–	–	–	757	**136**	862	665	–
	**45-3**	–	–	–	–	–	–	–	–	–	–	–	–	–	–	–	984	–	–	–	–
D. hupeana	**46-1**	–	–	–	–	–	–	–	–	–	–	–	–	–	–	–	–	–	–	654	–
	**46-2**	–	–	–	929	–	348	–	–	–	–	–	–	–	–	–	–	–	206	–	349
	**46-3**	–	–	–	–	–	–	388	–	872	–	236	–	–	–	875	–	–	–	438	–
	**46-4**	–	–	–	–	–	286	–	–	–	–	497	–	–	–	–	–	–	–	623	–
H. micrantha	**47-1**	–	–	–	–	–	–	–	–	–	–	–	–	–	–	–	618	487	869	–	–
	**47-2**	–	954	935	–	964	953	–	–	–	–	–	998	–	309	–	642	243	280	348	–
	**47-3**	–	–	–	–	983	–	–	–	–	–	–	–	–	–	–	567	779	–	213	–
	**47-4**	–	–	–	–	968	–	–	–	–	–	–	–	–	–	–	–	305	–	–	–
M. dodecandrum	**48-2**	932	–	–	–	–	985	–	–	–	–	–	–	–	–	–	960	–	–	322	955
	**48-3**	–	947	960	–	932	925	–	–	–	–	–	–	–	548	–	594	–	–	498	–
S. parasitica	**49-1**	–	–	–	–	–	–	–	–	–	–	–	–	–	–	–	–	–	–	993	–
	**49-2**	–	–	–	–	–	–	–	–	–	–	–	–	–	869	–	420	–	–	783	396
	**49-3**	–	–	–	–	–	–	–	–	–	–	–	–	–	–	–	838	484	–	–	–
	**49-4**	–	–	–	–	–	–	–	–	–	–	–	–	–	–	–	–	986	730	826	–
B. sinica	**50-2**	–	–	–	827	–	–	–	–	–	–	–	–	–	–	487	–	–	–	737	–
	**50-3**	–	–	–	–	–	275	497	–	–	274	893	–	796	–	–	–	**185**	–	264	–
	**50-4**	–	–	–	–	–	487	–	295	639	–	226	592	–	–	–	–	498	–	397	496
P. glaucus	**51-1**	–	–	–	–	986	–	–	–	–	–	–	–	–	–	–	321	–	–	–	–
	**51-2**	–	–	–	–	996	–	–	–	–	–	–	–	–	–	–	878	847	–	–	–
	**51-3**	–	–	–	–	874	574	–	910	–	–	–	–	–	985	–	481	990	958	805	–
	**51-4**	–	–	–	–	–	–	–	–	–	–	–	–	–	–	–	–	947	–	–	–
N. grossedentata	**52-1**	–	–	–	–	–	–	–	–	–	–	–	–	–	–	–	–	–	–	–	934
	**52-2**	563	–	–	–	–	–	–	–	–	–	–	–	–	–	–	–	–	–	747	629
	**52-3**	–	–	873	–	–	–	–	–	–	–	486	–	–	–	–	–	275	–	–	–
	**52-4**	–	–	–	–	–	**104**	–	–	–	–	296	–	–	–	–	–	982	–	598	–
P. quassioides's stem	**53-2**	–	–	–	–	–	863	–	–	–	–	–	–	–	–	–	–	–	–	–	–
	**53-3**	–	–	–	–	–	–	726	–	–	–	–	–	–	–	–	964	275	999	854	–
	**53-4**	–	–	–	973	–	972	–	–	–	–	865	–	–	–	–	–	–	–	905	–
P. quassioides's leaf	**54-2**	–	–	–	945	–	–	825	–	–	–	–	–	–	–	864	–	–	375	893	903
	**54-3**	–	–	–	–	–	928	–	–	–	–	–	–	–	–	–	–	–	–	694	872
	**54-4**	–	–	–	–	–	–	–	640	–	–	929	–	–	–	–	–	–	–	–	–
P. japonica	**55-2**	–	–	–	–	–	994	–	–	–	–	–	–	–	–	–	801	421	–	475	–
	**55-3**	–	–	–	–	–	–	–	–	–	–	–	–	–	–	–	589	643	–	–	–
P. angustifolia	**56-2**	–	–	–	–	–	–	–	–	–	–	–	–	–	–	–	797	310	248	493	–
	**56-3**	–	–	–	–	–	–	–	–	–	–	–	–	–	–	–	930	872	–	–	–
T. arguta	**57-1**	683	–	–	983	–	–	–	–	–	–	–	–	–	–	–	–	265	–	597	–
	**57-2**	–	–	–	985	–	590	–	–	–	–	–	–	–	–	–	–	564	–	**164**	295
	**57-3**	–	–	499	–	764	387	297	296	–	–	296	–	–	–	–	–	–	–	286	**185**
	**57-4**	–	–	896	–	985	386	286	499	–	–	396	–	–	–	–	–	–	–	510	497
Z. simulans	**58-1**	–	–	–	–	–	–	–	–	–	–	–	–	–	–	–	–	–	–	984	–
	**58-2**	–	–	–	–	–	848	–	–	–	–	–	–	–	–	–	–	895	–	399	298
	**58-3**	–	–	–	–	–	529	–	–	–	–	–	–	–	–	–	–	–	–	874	–
	**58-4**	–	–	–	–	–	268	–	–	–	–	–	–	–	–	–	–	–	–	468	–
A. elata	**59-1**	–	–	–	–	–	906	–	–	–	–	–	–	–	–	–	–	–	–	–	–
	**59-2**	–	–	–	–	–	463	–	–	–	–	–	–	–	–	–	–	–	–	995	974
	**59-3**	–	–	–	–	–	630	–	–	–	–	–	–	–	–	–	–	–	–	–	–
H. heptaphyllum	**60-1**	–	–	–	–	–	875	–	–	–	–	–	–	–	–	–	974	–	–	–	–
	**60-2**	–	–	–	–	–	360	–	–	–	–	–	–	–	–	–	527	748	–	439	–
F. japonica	**61-1**	–	–	–	–	–	892	–	–	–	–	–	–	–	597	–	372	–	808	850	–
	**61-2**	–	–	–	–	–	936	921	–	–	–	–	–	–	–	–	–	–	874	724	–
	**61-3**	–	–	–	–	–	659	–	–	–	–	–	–	–	–	–	–	–	640	–	–
	**61-4**	–	–	–	–	–	763	–	638	–	–	625	–	–	–	–	–	–	–	–	–
T. jasminoides	**62-1**	–	–	–	–	–	962	–	–	–	–	–	–	–	–	–	–	–	–	–	–
	**62-2**	–	–	–	–	928	994	–	–	–	–	–	–	–	–	–	749	465	–	501	–
	**62-3**	–	–	–	–	937	–	–	–	–	–	–	–	–	–	–	–	–	–	–	–
C. stauntonii	**63-2**	–	–	–	–	953	–	–	–	–	–	–	–	–	–	–	797	984	–	372	–
	**63-3**	–	–	–	–	954	–	–	–	–	–	–	–	–	951	–	–	–	–	–	–
P. angulata	**64-1**	–	–	–	–	–	874	–	–	–	–	–	–	–	–	–	–	895	–	763	942
	**64-2**	–	–	–	–	–	264	–	–	–	–	–	–	–	564	–	–	343	275	275	376
	**64-3**	**102**	975	–	–	–	473	–	–	–	–	–	–	–	–	–	–	–	–	955	–
	**64-4**	926	997	873	939	865	–	730	936	–	872	784	–	777	–	–	763	–	–	–	–
D. micrantha	**65-1**	–	–	–	–	–	–	–	–	–	–	–	–	–	–	–	–	–	–	497	–
	**65-2**	959	–	–	–	985	765	–	–	864	–	–	–	–	985	–	906	–	770	490	893
	**65-3**	–	–	–	–	975	–	–	–	–	–	–	–	–	–	–	–	–	–	486	–
E. alsinoides	**66-1**	–	–	–	–	–	–	–	–	–	–	–	–	–	–	–	–	–	874	874	–
	**66-2**	–	–	–	–	–	–	–	–	–	–	–	–	–	857	–	–	–	–	499	974
	**66-3**	–	–	–	–	–	468	–	–	–	–	–	–	–	663	–	–	875	–	274	968
	**66-4**	–	–	–	–	–	839	–	–	–	–	636	–	–	–	–	–	–	–	–	–
V. officinalis	**67-1**	–	–	–	–	–	938	–	–	–	–	–	–	–	–	–	**162**	–	–	–	–
	**67-2**	–	–	–	–	928	954	–	–	–	–	–	–	–	644	–	999	764	–	346	–
	**67-3**	–	–	–	–	–	764	–	–	–	–	–	–	–	–	–	749	–	–	–	–
V. negundo var. *cannabifolia*	**68-1**	–	–	–	–	–	694	–	–	–	–	–	–	–	–	–	851	–	–	495	994
	**68-2**	–	863	–	–	–	875	–	–	–	–	–	–	–	–	–	492	420	218	468	–
	**68-3**	–	–	–	–	–	928	–	–	–	–	–	–	–	–	–	871	941	705	–	–
O. vulgare	**69-1**	–	–	–	–	–	–	–	–	–	–	–	–	–	–	–	510	–	–	375	673
	**69-2**	–	–	–	895	–	–	–	–	–	–	–	–	–	–	–	397	–	**109**	234	**136**
	**69-3**	–	–	–	–	–	–	–	–	–	–	–	–	–	–	–	–	–	–	587	–
S. prionitis	**70-1**	–	–	–	–	–	276	–	–	928	–	–	–	873	287	–	–	276	537	287	211
	**70-2**	–	–	–	736	985	**199**	–	–	–	–	–	–	467	498	369	–	–	499	**176**	**117**
	**70-3**	–	–	–	–	–	–	–	–	–	–	–	–	–	347	–	–	–	–	538	–
	**70-4**	–	–	–	–	–	–	–	–	–	–	487	–	–	–	–	–	–	–	–	–
C. incana	**71-1**	–	–	–	–	746	636	–	–	–	–	–	–	–	–	–	–	–	–	–	–
	**71-2**	–	–	–	–	985	460	–	–	–	–	938	–	–	864	–	917	516	–	390	–
	**71-4**	–	–	–	–	–	874	–	–	–	–	–	–	–	–	–	–	–	–	–	–
M. scabra	**72-2**	–	–	–	–	983	973	–	–	–	–	–	–	–	745	–	569	510	907	342	979
	**72-3**	–	–	–	–	683	830	–	991	–	–	–	–	–	920	–	952	684	–	406	958
	**72-4**	–	–	–	–	–	355	–	–	–	–	–	–	–	–	–	–	–	**127**	422	565
B. lindleyana	**73-1**	–	–	–	–	–	–	–	–	–	–	–	–	–	–	–	965	–	–	376	984
	**73-2**	973	–	–	885	–	974	–	–	–	–	–	–	–	–	–	–	985	–	**194**	399
	**73-3**	888	988	983	–	–	966	–	–	–	–	–	–	–	–	–	–	–	–	984	–
	**73-4**	–	–	–	–	–	987	–	–	–	–	–	–	–	–	–	–	–	–	–	–
S. chinensis	**74-1**	873	–	–	–	892	–	–	–	–	–	–	–	–	–	–	–	330	–	–	–
	**74-2**	979	–	–	–	927	319	–	–	–	–	–	–	–	–	–	241	481	882	509	–
	**74-3**	970	–	–	–	887	731	983	–	–	980	967	–	–	–	–	–	–	–	–	–
S. cusia	**75-1**	875	–	–	–	–	239	–	–	–	–	–	–	–	–	–	–	–	388	–	–
	**75-2**	–	–	–	895	987	**158**	–	–	–	–	–	–	–	764	–	–	–	298	376	–
	**75-3**	–	–	–	–	–	349	–	–	–	–	–	–	904	–	–	–	–	–	966	–
	**75-4**	–	–	–	–	–	973	–	–	–	–	–	–	–	–	–	–	–	–	–	–
M. pubescens	**76-1**	–	–	–	–	–	–	–	–	–	–	–	–	–	–	–	–	–	–	937	–
	**76-2**	–	–	–	–	–	–	–	–	–	–	–	–	–	909	–	–	–	–	984	–
P. foetida	**77-1**	–	–	–	–	–	–	–	–	–	–	–	–	–	–	–	–	–	298	–	–
	**77-2**	–	–	–	–	–	–	–	–	–	–	–	–	–	–	–	–	–	–	388	–
	**77-3**	–	–	–	–	–	540	–	–	–	–	–	–	–	–	–	–	–	219	–	–
	**77-4**	–	–	–	–	–	358	–	498	–	–	910	–	–	–	–	–	–	–	–	–
H. mellii	**78-1**	–	–	–	–	–	–	–	–	–	–	–	–	–	–	–	–	–	–	984	998
	**78-2**	–	–	–	–	–	388	–	–	–	–	–	–	–	–	–	–	–	453	287	965
	**78-3**	–	–	–	–	–	530	–	–	–	–	–	–	–	–	–	–	–	–	798	–
	**78-4**	–	–	–	–	–	–	–	–	–	–	–	–	–	–	–	–	–	938	–	–
U. rhynchophylla	**79-1**	–	–	–	–	–	–	–	–	–	–	–	–	–	–	–	–	–	–	993	–
	**79-2**	–	–	–	–	–	–	–	–	–	–	–	–	–	832	–	–	–	–	–	–
	**79-3**	894	–	909	–	983	598	329	–	–	–	539	–	–	909	–	–	538	–	–	–
	**79-4**	–	–	–	–	–	–	–	–	–	–	–	–	–	–	–	–	275	–	–	–
S. japonica	**80-2**	–	–	–	–	649	–	–	–	–	–	–	–	–	–	–	879	993	–	477	992
	**80-3**	–	–	–	–	592	–	–	–	–	–	–	–	–	–	–	–	–	–	–	–
	**80-4**	–	–	–	–	–	596	–	–	–	–	–	–	–	–	847	–	–	271	957	923
L. japonica	**81-1**	–	–	–	–	–	–	–	–	–	–	–	–	–	–	–	–	–	–	429	–
	**81-2**	–	–	–	–	–	739	–	–	–	–	–	–	–	988	–	–	984	–	530	–
	**81-3**	–	–	–	–	–	640	–	–	–	–	–	–	–	–	–	–	–	–	908	–
	**81-4**	–	–	–	–	–	–	–	–	–	–	–	–	674	–	–	–	–	–	–	–
I. polycephala	**82-1**	–	–	–	–	–	–	–	–	–	–	–	–	–	–	–	703	–	–	–	–

[illegible]	**82-2**	–	–	–	–	785	974	–	–	–	–	–	–	–	–	–	678	528	–	–	–
	**82-3**	–	–	–	–	596	–	–	–	–	–	–	–	–	–	–	–	–	–	–	–
	**82-4**	890	–	–	–	833	–	–	–	–	–	–	–	–	–	–	–	–	–	–	–
E. prostrata	**83-1**	–	–	–	–	925	–	–	–	–	–	–	–	–	–	–	–	527	–	–	–
	**83-2**	–	–	–	–	584	873	–	–	–	–	–	–	–	985	–	871	348	882	993	–
	**83-3**	–	–	–	–	738	464	–	–	–	–	–	–	–	999	–	866	956	–	–	–
S. decurrens	**84-1**	–	–	–	–	–	–	–	–	–	–	–	–	–	–	–	–	–	538	928	–
	**84-2**	–	–	–	–	–	982	–	–	–	–	–	–	–	–	–	–	631	321	592	395
	**84-3**	–	–	–	–	–	–	–	–	–	–	–	–	–	–	–	–	–	–	499	–
	**84-4**	–	–	–	–	–	540	–	429	–	–	–	–	–	–	–	–	–	–	905	–
A. pekinensis	**85-1**	–	–	–	–	–	–	–	–	–	–	–	–	–	–	–	–	–	993	624	872
	**85-2**	–	–	–	–	–	–	–	–	–	–	–	–	–	–	–	–	–	–	309	398
	**85-3**	–	–	–	–	–	738	–	–	–	–	–	–	–	–	–	–	–	536	845	467
C. crepidioides	**86-1**	–	–	–	–	–	792	–	–	–	–	–	–	–	–	–	–	–	–	834	–
	**86-2**	–	–	–	–	–	637	–	–	–	–	–	–	–	898	–	–	493	–	205	209
	**86-3**	–	–	–	–	995	982	–	–	–	–	–	–	–	–	–	–	–	–	264	**182**
	**86-4**	–	–	–	–	–	786	786	–	–	509	992	–	–	753	–	–	–	539	**184**	530
B. pilosa	**87-1**	–	–	–	–	–	–	–	–	–	–	–	–	–	–	–	–	–	–	–	994
	**87-2**	–	–	–	–	–	–	–	–	–	–	–	–	–	–	–	–	–	–	994	–
	**87-3**	–	–	–	–	–	–	–	–	–	–	–	–	–	–	–	–	–	–	983	–
D. cappa	**88-1**	–	–	–	–	–	928	–	–	–	–	–	–	–	–	–	–	–	395	492	–
	**88-2**	–	–	–	927	–	592	–	–	–	–	–	–	–	–	–	620	–	–	209	–
	**88-3**	–	–	–	–	–	–	–	–	–	–	–	–	–	–	–	304	–	318	–	–
	**88-4**	–	–	–	–	–	–	–	–	–	–	–	–	–	–	–	953	–	–	–	–
A. gramineus	**89-1**	–	–	–	–	–	–	–	–	–	–	–	–	–	–	–	**185**	251	–	–	–
	**89-2**	–	840	–	–	875	937	–	–	–	–	–	–	–	–	–	939	334	239	489	–
	**89-3**	–	–	–	–	808	–	–	–	–	–	–	–	–	–	–	710	702	–	–	–
B. japonicus	**90-1**	–	–	–	–	–	–	–	–	–	–	–	–	–	–	–	824	–	982	–	–
	**90-2**	–	–	–	–	–	–	–	–	–	–	–	–	–	835	–	–	–	539	829	–
	**90-4**	–	–	–	–	–	827	–	–	–	–	–	–	–	–	–	–	724	–	–	–
L. gracile	**91-2**	898	–	–	–	847	983	–	–	–	–	–	–	–	–	–	957	414	–	475	–
	**91-3**	–	–	–	–	983	–	–	–	–	–	–	–	–	–	–	575	–	–	471	793
	**91-4**	–	–	–	–	–	–	970	–	–	–	–	–	–	–	957	–	–	–	–	878
Z. officinale	**92-2**	–	–	–	–	883	–	–	–	–	–	–	–	–	–	–	–	–	–	–	–
	**92-3**	–	–	–	–	983	–	–	–	–	–	–	–	–	–	–	–	–	–	–	–
	**92-4**	–	–	–	–	–	–	–	–	–	–	687	–	–	–	–	–	–	–	–	–
A. zerumbet	**93-1**	–	–	–	–	–	238	259	–	–	–	–	–	425	–	–	427	–	598	523	295
	**93-2**	–	–	–	894	–	264	–	–	–	–	693	–	–	–	–	–	387	–	299	–
	**93-3**	–	–	–	–	–	874	774	–	–	757	–	–	783	–	–	898	–	–	983	–
	**93-4**	–	–	–	–	–	910	–	–	–	–	640	–	–	–	–	–	634	–	884	984
A. japonica's herb	**94-1**	–	–	–	–	–	–	–	–	–	–	–	–	–	–	–	–	935	–	436	760
	**94-2**	–	–	–	874	–	–	–	–	–	–	–	–	–	–	–	–	–	490	238	376
	**94-4**	–	–	–	–	–	–	794	–	–	–	–	–	–	–	–	–	–	–	–	–
A. japonica's fruit	**95-1**	–	829	–	–	–	–	–	–	–	–	–	–	–	–	–	–	–	639	**136**	–
	**95-2**	733	–	–	874	–	–	–	–	–	–	–	–	284	–	–	635	–	–	297	–
	**95-3**	–	–	–	–	–	–	–	–	–	–	–	–	–	–	–	456	–	–	437	–
	**95-4**	–	–	–	–	–	–	–	–	–	–	983	–	–	872	–	–	792	–	–	–
S. riparia	**96-2**	–	–	–	–	833	–	–	–	–	–	–	–	–	–	–	–	–	–	863	–
	**96-3**	–	–	–	–	995	–	–	–	–	–	–	–	–	–	–	–	–	–	782	–
S. glabra	**97-1**	–	–	–	–	984	–	–	–	–	–	–	–	–	–	–	–	–	–	–	–
	**97-2**	–	–	–	–	898	984	–	–	–	–	–	–	–	926	–	805	635	–	201	–
	**97-3**	798	–	–	–	786	764	–	–	–	–	–	–	–	–	–	997	–	540	**181**	932
	**97-4**	–	–	–	–	623	596	–	–	–	–	–	–	–	–	–	–	–	–	383	–
**sum***	**331**	**39**	**30**	**15**	**35**	**87**	**168**	**37**	**22**	**12**	**20**	**49**	**7**	**30**	**50**	**18**	**95**	**117**	**83**	**207**	**110**

No.1 to97: Hakka herbs listed in Table 1; Solvent (-1 to -4): Hexane, Ethyl acetate, Methanol, Water; * The total number of active extracts against each microorganism;

IC_{50} values less than 200 µg/mL were marked as bold;

Fungal strains:*Saccharomyces cerevisiae* (SC), *Candida auris* (CAU), *Candida glabrata* (CG), *Candida albicans* (CA) and *Candida parapsilosis* (CP);

Gram-negative bacteria: *Brevundimonas diminuta* (BD), *Shigella flexneri* (SF), *Acinetobacter baumannii* (AB), *Pseudomonas aeruginosa* (PA), *Salmonella enterica* subsp. *enterica* (SLE), *Aeromonas hydrophila* (AH), *Enterobacter aerogenes* (EA), *Escherichia coli* (EC) and *Shigella sonnei* (SS);

Gram-positive bacteria: *Staphylococcus epidermidis* (SE), *Bacillus cereus* (BC), *Staphylococcus aureus* (SA), *Enterococcus faecalis* (EF), *Micrococcus luteus* (ML), and *Listeria innocua* (LI).
