# Supplementary material for: Ethnobotanical study of Hakka traditional medicine in Ganzhou, China and their antibacterial, antifungal, and cytotoxic assessments
Source: BMC Complement Med Ther. 2022 Sep 19;22:244. doi: 10.1186/s12906-022-03712-z (PMC9484230; doi:10.1186/s12906-022-03712-z)
Supplement: Supplementary file 5 — Additional file 5. [file 12906_2022_3712_MOESM5_ESM.pdf]

Supplementary Material 5 - Viability inhibition (%) of 97 HTMs' extracts against cancerous and noncancerous cell lines

Herb No.	Plant name	A549				WI26-VA4			
		**Hexane**	**Ethyl acetate**	**Methanol**	**Water**	**Hexane**	**Ethyl acetate**	**Methanol**	**Water**
1	*L. japonicum*	0	60	40	-7	14	17	16	-9
2	*S. tamariscina*	-12	-22	-37	-20	-4	-4	-12	4
3	*S. moellendorffii*	-15	-13	-39	-3	-5	-4	1	1
4	*O. chinensis*	26	43	40	4	-11	-1	-12	-10
5	*A. flabellulatum*	-8	24	12	1	-7	14	21	23
6	*S. hastata*	-27	-23	24	-16	-41	7	-5	-1
7	*L. salicifolia*	-23	-39	-33	-33	-21	-22	-18	-5
8	*E. ramosissimum*	76	78	77	53	-40	1	-26	-7
9	*F. oldhamii* **(root)**	93	86	48	63	5	4	3	18
10	*F. oldhamii* **(stem)**	-31	21	-37	-26	-22	-13	-7	-14
11	*F. oldhamii* **(leaf)**	59	58	25	53	5	18	23	28
12	*C. grammatus*	78	83	76	14	-13	9	4	11
13	*L. glauca*	68	65	66	-25	-37	-35	2	10
14	*C. jensenianum*	-4	17	54	-13	20	43	34	-21
15	*S. chinensis*	78	63	72	61	-15	9	-4	-7
16	*P. wallichii*	0	13	16	-7	-14	1	-2	5
17	*A. caudigerum*	-16	-28	-34	-31	-24	-6	12	-2
18	*S. japonica*	33	72	77	77	-46	-11	62	90
19	*L. formosana*	55	29	91	-11	2	-1	14	-25
20	*S. cathayensis* **(tender stem and leaf)**	15	23	31	28	-28	39	7	5
21	*S. cathayensis* **(root)**	-16	32	-21	-1	-7	20	6	17
22	*D. macropodum*	-32	75	35	-35	11	12	17	28
23	*F. pumila*	86	49	39	27	-23	4	9	12
24	*F. formosana* f. *shimadai*	13	4	8	-12	5	17	92	27
25	*M. cochinchinensis*	67	100	89	-17	30	89	76	12
26	*F. simplicissima*	3	-14	8	5	-32	-1	-13	9
27	*B. nivea*	-34	39	37	-13	-38	-8	6	3
28	*E. involucratum*	31	64	56	-33	-22	-22	-8	-9
29	*P. americana*	13	49	21	43	-19	-5	12	13
30	*P. chinense*	19	44	77	78	8	18	1	-4
31	*P. chinensis* var. *paradoxa*	-37	69	45	55	-3	-2	-3	1
32	infected leaf of *C. oleifera*	-35	-3	-17	-4	-14	10	50	22
33	*A. nitida*	23	90	23	49	18	3	2	17
34	*E. acuminatissima*	15	73	-1	-7	7	10	8	-38
35	*H. japonicum*	-3	49	9	4	-1	20	8	0
36	*C. crenata*	71	39	74	-34	5	0	-15	-8
37	*U. lobata*	14	3	29	2	-15	3	37	-25
38	*P. stenoptera*	6	-1	64	5	-33	-22	37	15
39	*L. alfredii*	46	80	80	43	-2	-6	-15	-5
40	*L. fortunei*	-1	75	72	73	-48	76	10	42
41	*D. febrifuga*	44	70	59	-40	-12	-8	-49	-4
42	*S. stolonifera*	-34	21	39	-10	3	6	-3	0
43	*A. pilosa*	73	-18	-33	49	-9	-31	15	19
44	*C. dielsiana*	46	56	63	11	9	12	4	7
45	*M. officinalis*	-9	-24	-15	-9	10	14	-32	-19
46	*D. hupeana*	-1	56	70	72	-50	-33	-36	-20
47	*G. micranthus*	-11	19	51	7	3	64	38	41
48	*M. dodecandrum*	1	-5	15	10	-7	-34	10	8
49	*S. parasitica*	-31	79	77	9	-6	12	9	18
50	*B. sinica*	41	74	64	76	-8	-12	-6	-15
51	*P. glaucus*	11	5	55	6	-4	20	70	-8
52	*N. grossedentata*	7	5	35	8	-4	4	0	-4
53	*P. quassioides* **(stem)**	23	10	95	38	-11	-5	6	6
54	*P. quassioides* **(leaf)**	33	73	67	30	-34	-40	0	15
55	*P. japonica*	-26	-10	50	-11	7	14	63	-9
56	*P. angustifolia*	-12	-17	35	-2	-4	-25	31	11
57	*T. arguta*	35	91	92	92	-22	-37	-5	23
58	*Z. simulans*	-37	77	79	20	-10	3	3	-5
59	*A. elata*	-40	-30	21	27	-28	-19	-46	-35
60	*H. heptaphyllum*	-2	92	93	52	-19	70	93	-12
61	*F. japonica*	37	78	31	41	-42	-33	-1	-9
62	*T. jasminoides*	0	7	16	5	11	-37	9	16
63	*C. stauntonii*	12	-3	0	3	-12	18	16	3

64	*P. angulata*	78	73	81	78	-10	-13	-25	2
65	*D. micrantha*	-1	-1	-9	-8	15	-21	-23	-19
66	*E. alsinoides*	70	49	79	-18	-14	-1	-4	-1
67	*V. officinalis*	6	94	22	6	2	38	26	-1
68	*V. negundo* var. *cannabifolia*	3	90	22	6	-23	91	27	7
69	*O. vulgare*	23	70	75	-28	-6	-8	-35	-26
70	*S. prionitis*	68	64	80	79	-3	-9	-3	-18
71	*C. incana*	9	11	12	4	7	43	27	-9
72	*M. scabra*	12	38	52	9	-3	71	52	4
73	*B. lindleyana*	40	92	94	94	15	9	-4	-7
74	*S. chinensis*	5	5	11	10	11	7	7	19
75	*S. cusia*	-15	93	61	7	-15	3	1	10
76	*M. pubescens*	25	32	20	6	2	-20	-12	10
77	*P. foetida*	17	13	10	1	16	14	20	19
78	*H. mellii*	68	64	61	-34	-8	-10	-29	-18
79	*U. rhynchophylla*	-32	21	76	-31	-17	-13	-7	16
80	*S. japonica*	14	6	3	7	-9	23	14	-2
81	*L. japonica*	-39	-33	-38	-31	-38	-38	-47	-25
82	*I. polycephala*	5	-8	-4	5	-29	16	-1	3
83	*E. prostrata*	8	45	47	2	-22	33	54	-41
84	*S. decurrens*	10	47	-15	13	-18	-3	15	14
85	*A. pekinensis*	-35	74	74	16	-16	-11	4	-38
86	*C. crepidioides*	45	77	49	-7	76	-35	-9	3
87	*B. pilosa*	25	55	61	-36	-5	-12	-14	-19
88	*D. cappa*	52	43	43	-3	13	10	15	0
89	*A. gramineus*	-11	62	-3	0	-12	68	51	18
90	*A. japonicus*	-39	-26	3	-2	-32	-30	13	18
91	*L. gracile*	-22	1	-34	-6	-12	6	-25	9
92	*Z. officinale*	1	94	-2	4	-25	93	-4	-4
93	*A. zerumbet*	78	79	77	36	-40	-30	20	14
94	*A. japonica* **(herb)**	81	81	-12	79	20	23	19	19
95	*A. japonica* **(friut)**	60	65	20	46	-40	-29	-40	17
96	*S. riparia*	-17	-18	-17	-17	-1	-6	12	-32
97	*S. glabra*	11	8	51	5	-23	-34	71	10
